# Single-cell RNA-seq uncovers dynamic processes orchestrated by RNA-binding protein DDX43 in chromatin remodeling during spermiogenesis

**DOI:** 10.1038/s41467-023-38199-w

**Published:** 2023-04-29

**Authors:** Huanhuan Tan, Weixu Wang, Congjin Zhou, Yanfeng Wang, Shu Zhang, Pinglan Yang, Rui Guo, Wei Chen, Jinwen Zhang, Lan Ye, Yiqiang Cui, Ting Ni, Ke Zheng

**Affiliations:** 1 State Key Laboratory of Reproductive Medicine, Nanjing Medical University, 211166 Nanjing, China; 2 State Key Laboratory of Genetic Engineering, Collaborative Innovation Center of Genetics and Development, Human Phenome Institute, Shanghai Engineering Research Center of Industrial Microorganisms, School of Life Sciences and Huashan Hospital, Fudan University, 200438 Shanghai, China; 3 Present Address: Reproductive Medicine Center, The First Affiliated Hospital of Chongqing Medical University, No. 1 Youyi Road, 400016 Chongqing, Yuzhong District China; 4 Present Address: Institute of Computational Biology, Helmholtz Center Munich, Munich, Germany

**Keywords:** Spermatogenesis, Gene regulatory networks, Transcriptomics

## Abstract

Mammalian spermatogenesis shows prominent chromatin and transcriptomic switches in germ cells, but it is unclear how such dynamics are controlled. Here we identify RNA helicase DDX43 as an essential regulator of the chromatin remodeling process during spermiogenesis. Testis-specific *Ddx43* knockout mice show male infertility with defective histone-to-protamine replacement and post-meiotic chromatin condensation defects. The loss of its ATP hydrolysis activity by a missense mutation replicates the infertility phenotype in global *Ddx43* knockout mice. Single-cell RNA sequencing analyses of germ cells depleted of *Ddx43* or expressing the *Ddx43* ATPase-dead mutant reveals that DDX43 regulates dynamic RNA regulatory processes that underlie spermatid chromatin remodeling and differentiation. Transcriptomic profiling focusing on early-stage spermatids combined with enhanced crosslinking immunoprecipitation and sequencing further identifies *Elfn2* as DDX43-targeted hub gene. These findings illustrate an essential role for DDX43 in spermiogenesis and highlight the single-cell-based strategy to dissect cell-state-specific regulation of male germline development.

## Abstract (web summary)

Germ cells undergo dramatic chromatin and transcriptomic changes during spermatogenesis, though how this is controlled is not well established. Here they show that RNA-binding protein DDX43 directs the spermatid differentiation trajectory and regulates chromatin remodeling in spermiogenesis, which is partially mediated by its target gene *Elfn2*.

## Introduction

After meiosis, haploid spermatids undergo spermiogenesis, which is characterized by a cascade of chromatin remodeling events that culminate in the replacement of most histones by protamines^1–4^. This stepwise process entails massive histone modifications as well as transient formation of DNA strand breaks^5–8^. Genomic DNA is compacted to high orders, giving rise to a firm sperm head with greatly condensed nucleus that ensures stable intergenerational transmission^5,9^. While causality is well-established linking histone-to-protamine transition to male fertility^3,10,11^, recent studies give mechanistic insights into how this transition is target-specifically controlled by several important proteins^5,6,12–16^. Despite much progress, it remains obscure whether chromatin remodeling events are orchestrated at transcriptome-wide level.

Round spermatids differentiate through eight typical steps till they become elongating and then elongated, during which transcription is converted gradually from being active to inert^17,18^. Previous studies often selected postnatal time points to enrich or even purify certain types of germ cells for either dynamics characterization, or for an inter-genotypic comparison. As total RNA-seq only reveals an ensemble average level of a mixed cell population at a fixed time point, such traditional strategies pose a difficulty in disentangling profound cellular heterogeneity. To put it differently, the static snapshots of the morphology-based, but not intrinsic, molecular alterations do not allow for tracing the footprint of contiguous cell states. In recent years, single-cell RNA-seq (scRNA-seq) has provided an effective avenue for discriminating a vast number of individual germ cells based on their differential transcriptomic patterns^19–25^. For example, scRNA-seq has depicted the functional distinction between round spermatid subpopulations in their potential for embryo development, reflecting intrinsic subtypes^19^. To achieve developmental lineage tracing of single cells, some computational tools, such as ICAnet^26^ and scVelo^27^, are built effective for the inference of differentiation hierarchy and trajectory. By virtue of single-cell-based methodologies, it is therefore promising to elucidate how inter-subtype transitions of varying transcriptomic states of spermatids are regulated, presumably by an RNA-binding protein^28,29^.

RNA helicases are RNA-binding proteins with versatile functionality^30,31^. While the majority of RNA helicases are expressed across multiple tissues, some play specific roles in the germline^32–35^. We notice that DEAD-box helicase protein DDX43 is highly expressed in testis and many tumors. Whereas in vitro biochemical studies have demonstrated DDX43 serves as a bona fide helicase^36^, its function in the testis remains unknown.

In this work, we integrated multiple approaches to query the biological and molecular roles of DDX43 in spermatogenesis. Genetic mutation of *Ddx43* leads to deficiency in a cascade of chromatin remodeling events. Using scVelo to infer germ cell developmental trajectory and velocity-directed Markov chain simulation to predict the differentiation terminal, we found that spermatids have a pronounced alteration in the differentiation trajectory, in concert with their histochemical defects, in *Ddx43* mutant mice. Coupling with eCLIP-seq, we anchored an early subpopulation of post-meiotic spermatids as initial targets of DDX43 and further identified *Elfn2* as a hub gene downstream of DDX43. These findings together reveal DDX43 operates an early gene expression network to maintain the homeostasis of subsequent chromatin dynamics in spermatids.

## Results

### DDX43 expression dominates around meiotic exit

**Fig. 1:**
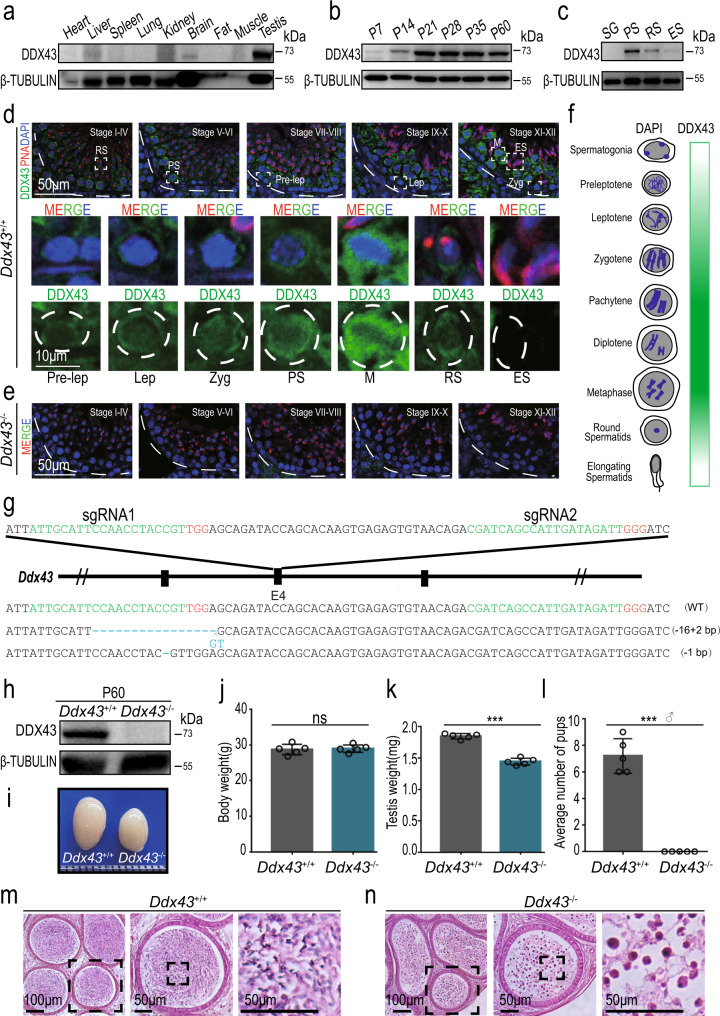
DDX43 is enriched in mouse testis and indispensable for male fertility. **a**–**c** Western blot analyses of DDX43 protein in extracts from P60 mouse tissues (**a**), testes collected from mice at different ages (**b**), and purified spermatogenic cells (**c**) including spermatogonia (SG), pachytene spermatocytes (PS), round spermatids (RS), and elongating spermatids (ES). β-TUBULIN serves as a loading control. **d**, **e** Immunofluorescence staining of DDX43 (green) and PNA (red) on testis sections from adult wild-type (**d**) and *Ddx43*^–/–^ (**e**) mice. DNA was counterstained with DAPI. Lower panels show magnification of the boxed areas in the upper panels. Pre-lep, pre-leptotene spermatocyte; Lep, leptotene spermatocyte; Zyg, zygotene spermatocyte; PS, pachytene spermatocyte; M, metaphase cell; RS, round spermatid; ES, elongating spermatid. Stages of seminiferous tubule are denoted by Roman numerals. Scale bars are indicated for all panels of each group. **f** Graphic representation of developmental expression pattern of DDX43 in male germ cells. **g** Schematic diagram of CRISPR-Cas9 gene editing to create the *Ddx43* knockout mice. Green: sgRNA targeting sequences; blue: mutated sequences; red: PAM nucleotides. **h** Western blot confirms absence of DDX43 protein in P60 *Ddx43*^–/–^ testis. β-TUBULIN serves as a loading control. **i** Representative image of an atrophied testis from P60 *Ddx43*^–/–^ mice compared with that from *Ddx43*^+/+^. **j**–**l** Comparative analyses of body weight (**j**), testis weight (**k**), and male fertility (**l**) between adult *Ddx43*^+/+^ and *Ddx43*^–/–^ mice. Male mice were naturally crossed with age-matched wild-type female mice. *N* = 5 for each genotype. ns, not significant; *P* = 0.8033 (**j**), *** *P* = 0.0003 (**k**), *** *P* = 0.0002 (**l**); two-tailed paired Student’s *t* test. Each bar represents the mean ± SD from biological replicates. **m**, **n** Hematoxylin and Eosin (H&E) staining on epididymis sections from adult *Ddx43*^+/+^ (**m**) and *Ddx43*^–/–^ (**n**) mice. Scale bars are indicated. Each experiment was repeated three times with similar results.

To begin characterizing DDX43 distribution, Western blot analysis of mouse tissues showed that DDX43 was not ubiquitously expressed, but testis-enriched (Fig. 1a), consistent with the results detected by quantitative reverse transcription followed by polymerase chain reaction (qRT-PCR) (Supplementary Fig. 1a). In the testis, *Ddx43* transcript levels increased sharply at postnatal day 14 (P14) (Supplementary Fig. 1b). In parallel, DDX43 protein abundance increased sharply at P21, a time point when late-stage spermatocytes differentiate into round spermatids, and maintained at higher levels thereafter (Fig. 1b). Specifically, it was abundant predominantly in pachytene spermatocytes and secondly in round spermatids (Fig. 1c and Supplementary Fig. 1c), shown from both protein and mRNA levels in these two types of isolated germ cells (Supplementary Fig. 1d–f).

Immunofluorescence in adult testis sections, as well as Western blot in subcellular fractions, revealed that DDX43 protein distributed over nucleus and cytoplasm (Fig. 1d and Supplementary Fig. 1g). Co-immunofluorescence of Lectin-PNA, an acrosome marker, allowed us to visualize DDX43 spatiotemporally dynamic from stages I to XII of seminiferous tubules and accordingly across different steps of germ cells, e.g., step 1–16 spermatids (Fig. 1d). Overall, DDX43 protein was predominantly cytoplasmic and also present in nucleus, whereas its levels increased with meiotic progression till exit (Fig. 1d). The immunofluorescence specificity of DDX43 was proved by comparing with *Ddx43* knockout (KO) mice (*Ddx43*^–/–^; see next paragraph) as negative control (Fig. 1e). In detail, DDX43 was weakly detected from preleptotene (stages VII–VIII) to zygotene (stages XI–XII), at much higher levels in pachytene spermatocytes, continuously rising until the expression peaked at meiotic metaphase, and still present in early round spermatids, but decreased after step 8 (stage VIII) (Fig. 1d, f). Taken together, DDX43 is nucleocytoplasmic and mostly expressed before and after meiotic exit, implicating its major function around this timing.

### Spermiogenesis is defective in *Ddx43* mutant mice

To elucidate the biological function of *Ddx43*, we first generated two *Ddx43* KO lines by CRISPR-Cas9 gene-editing approach. Two small guide RNAs (sgRNAs) were used to target exon 4 of *Ddx43* gene (Fig. 1g), producing two mutant founder lines (Fig. 1g and Supplementary Fig. 2a). KO Line #1 has an insertion of 2-bp (base pair) accompanied by a 16-bp deletion; KO Line #2 has a deletion of a single bp. Both alleles result in reading frameshifts and premature termination codons. Genotyping PCR and RT-PCR confirmed the absence of full-length transcripts in mice homozygous for the KO allele (hereafter referred to as *Ddx43*^−/−^), indicating successful targeting of *Ddx43* gene (Supplementary Fig. 2b–d). Western blot and immunostaining analyses demonstrated absence of DDX43 protein in testes from adult *Ddx43*^−/−^ mice (Fig. 1e, h).

Mice of all *Ddx43* genotypes were matured to adulthood normally without apparent developmental defects. Compared with age-matched wild-type mice, the body weight was similar (Fig. 1j), but *Ddx43*^−/−^ testes looked smaller with a lower weight (Fig. 1i, k). Strikingly, no pups were produced from adult *Ddx43*^−/−^ males in mating tests (Fig. 1l). In contrast to wild-type epididymis teemed with spermatozoa (Fig. 1m), germ cell numbers in *Ddx43*^–/–^ mice were drastically reduced and no normal spermatozoa were present; instead, sloughed irregular spermatids and cell debris were observed (Fig. 1n).

**Fig. 2:**
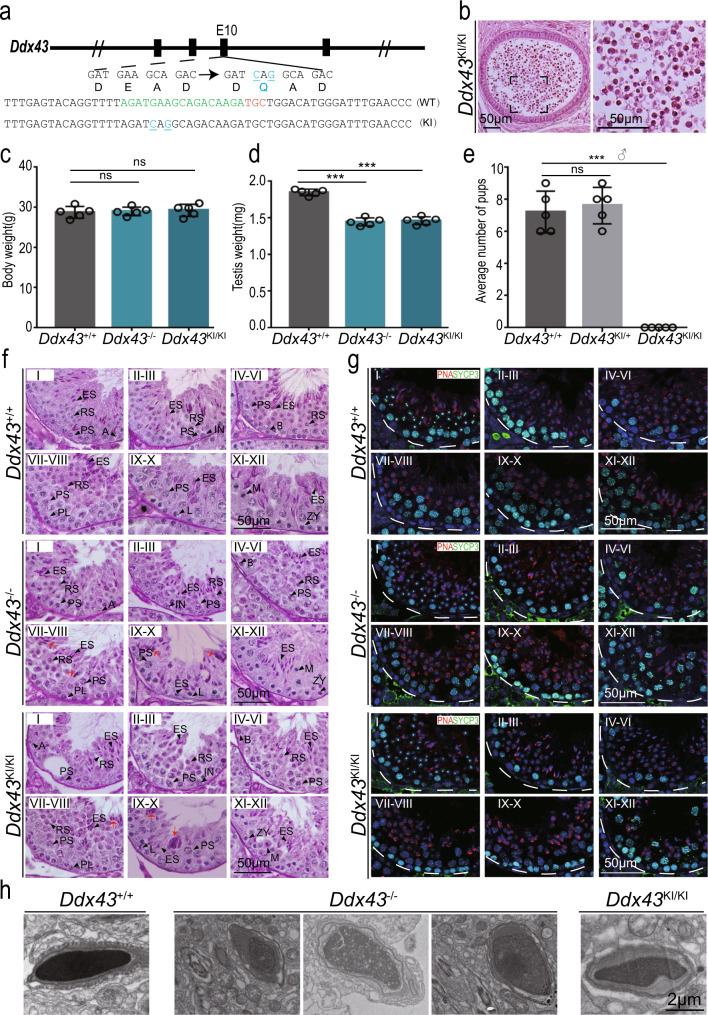
DDX43 mutant mice exhibit defects in spermatids. **a** Using CRISPR-Cas9 to create the ATPase-dead *Ddx43* knock-in mice that carry a missense mutation (DEAD to DQAD) in the ATPase motif. Green: sgRNA targeting sequences; blue: mutated sequences; red: PAM nucleotides. **b** H&E staining on epididymis sections from adult *Ddx43*^KI/KI^ mice. *Ddx43*^KI/KI^ epididymis is void of normal spermatozoa that abounds in *Ddx43*^+/+^ (Fig.1m), similar to *Ddx43*^–/–^ (Fig.1n). Scale bars are indicated. **c**, **d** Comparative analyses of body weight (**c**) and testis weight (**d**) between adult *Ddx43*^+/+^, *Ddx43*^-/-^, and *Ddx43*^KI/KI^ mice. *N* = 5 for each genotype. *P* values were calculated by oneway ANOVA. ns, not significant; *P* = 0.9621 (**c**; *Ddx43*^+/+^ vs *Ddx43*^–/–^), *P* = 0.6938 (**c**; *Ddx43*^+/+^ vs *Ddx43*^KI/KI^), ***P = 0.0007 (**d**; *Ddx43*^+/+^ vs *Ddx43*^–/–^), ***P = 0.0003 (**d**; *Ddx43*^+/+^ vs *Ddx43*^KI/KI^). Each bar represents the mean ± SD from biological replicates. **e** Fertility test on adult *Ddx43*^+/+^, *Ddx43*^KI/+^, and *Ddx43*^KI/KI^ mice naturally crossed with age-matched wild-type female mice. *N* = 5 for each genotype. *P* values were calculated by oneway ANOVA.ns, not significant; *P* = 0.8594 (*Ddx43*^+/+^ vs *Ddx43*^KI/+^), ***P = 0.0006 (*Ddx43*^+/+^ vs *Ddx43*^KI/KI^). Each bar represents the mean ± SD from biological replicates. **f** PAS-hematoxylin staining of testis sections from adult *Ddx43*^+/+^, *Ddx43*^KI/KI^ and *Ddx43*^–/–^ mice. Stages of seminiferous tubule are denoted by Roman numerals. A, type A spermatogonia; IN, intermediate spermatogonia; B, type B spermatogonia; PL, preleptotene spermatocyte; L, leptotene spermatocyte; ZY, zygotene spermatocyte; PS, pachytene spermatocyte; M, metaphase cell; RS, round spermatid; ES, elongating spermatid. Red arrows point to abnormal spermatids at step 16. Scale bars are indicated. **g** Immunofluorescence staining of SYCP3 (green) and PNA (red) for observation of abnormal spermatids at specific stages of spermatogenesis, parallel to (**f**). DNA was counterstained with DAPI. Scale bars are indicated. **h** Representative images of transmission electron microscopy of sperm heads from adult wild-type and mutant mice. Scale bars are indicated. Each experiment was repeated three times with similar results.

DDX43 is an explicit ATP-driven RNA helicase in possession of RNA binding and unwinding activities^36–38^, and therefore a potential mediator of dynamic protein-RNA interactions^31^. That means a missense mutation (DQAD) within DEAD-box will inactivate DDX43 ATP hydrolysis and lock its bound RNA targets to prevent their turnover. Hence, an in vivo ATP hydrolysis-dependent mechanism is worth probing for DDX43, like the case of MVH^39,40^. To this end, we generated *Ddx43* knock-in (KI) mice (referred to as *Ddx43*^KI/KI^) (Fig. 2a and Supplementary Fig. 3a). Adult *Ddx43*^KI/KI^ male mice phenocopied *Ddx43*^−/−^, manifested with smaller testis (Fig. 2d), male sterility (Fig. 2e), and no normal mature sperm (Fig. 2b), yet, with comparable body weight (Fig. 2c).

While the RNA abundance of *Ddx43* in *Ddx43*^KI/KI^ testes was still comparable to wild type (Supplementary Fig. 3b, c), DDX43 protein was undetectable not only in total lysates (Supplementary Fig. 3d), unless after immunoprecipitation (IP) enrichment (Supplementary Fig. 3e), but also in cellular subfractions (Supplementary Fig. 3f, g). As the helicase-specific role of DDX43 was masked by the unexpected ablation of the ATPase-dead DDX43 protein per se, KI males exhibited KO-similar defects in sperm production. We then subjected both or either of the two mutant models to subsequent studies.

Germ cells in both mutant testes displayed comparable behavior at P21, differentiation differences at P28, a time point coinciding with the appearance of elongating spermatids, and pronounced flaws at P35 (Supplementary Fig. 2e). To define defects in each step of germ cells, we performed staining using Periodical-Aid-Schiff (PAS) (Fig. 2f) and co-immunostaining for SYCP3 and PNA in adult testis sections (Fig. 2g). *Ddx43*^−/−^ and *Ddx43*^KI/KI^ seminiferous tubules presented fewer elongated spermatids relative to wild type, and lacked normal spermatozoa (Fig. 2f, g). At stage VIII, most *Ddx43* mutant step 16 spermatids showed a failure of normal spermiation with irregular head shape and excess cytoplasm (Fig. 2f; red arrows). These condensed rod- or round-like abnormal elongated spermatids frequently appeared in stages IX–XI (Fig. 2f; red arrows). In *Ddx43* mutant epididymis, although there were only an extremely low number of sperm, we still enriched a few and observed that their nuclei were more efficiently stained with acidic aniline (Supplementary Fig. 2f), indicative of less condensed chromatin in these mutant sperm. Transmission electron microscopy (TEM) further revealed that sperm heads in *Ddx43* mutant mice were less condensed with uneven density and malformed structure (Fig. 2h). Taking account all these observations, we conclude that DDX43 deficiency leads to defects in spermiogenesis and spermatid chromatin.

### DDX43 deficiency disturbs chromatin remodeling

**Fig. 3:**
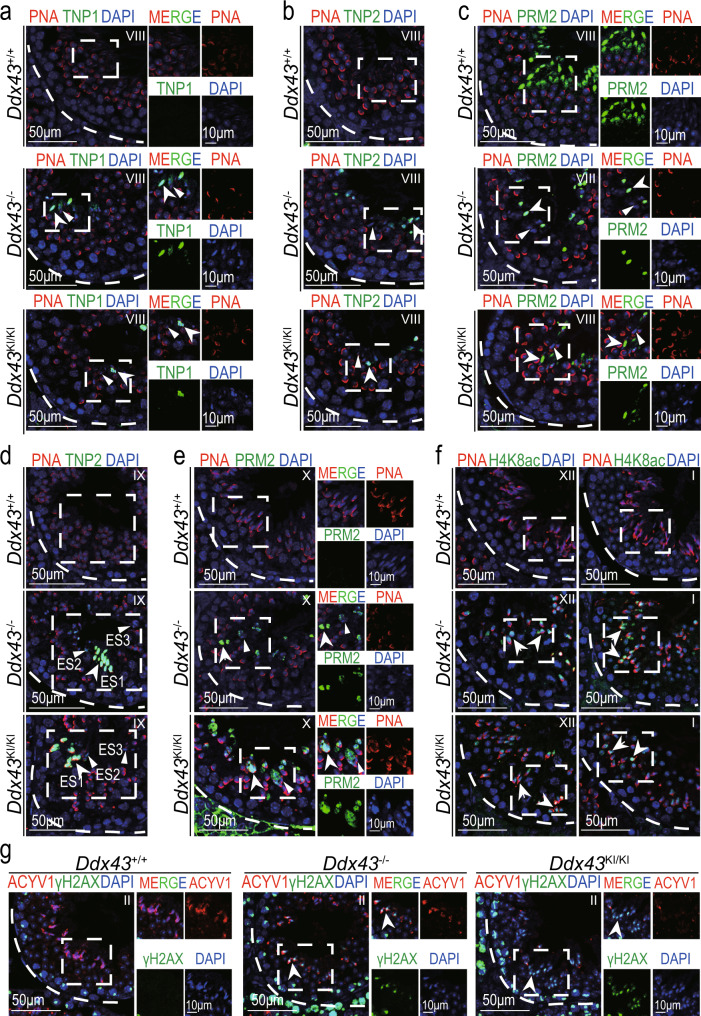
DDX43 deficiency impairs chromatin remodeling during spermiogenesis. **a**–**g** Immunofluorescence analyses on adult *Ddx43*^+/+^, *Ddx43*^KI/KI^ and *Ddx43*^–/–^ mouse testes with the following combinations. **a** TNP1 (green) and PNA (red), see also Supplementary Fig. 4a; (**b**, **d**) TNP2 (green) and PNA (red), see also Supplementary Fig. 4b; (**c**, **e**) PRM2 (green) and PNA (red); (**f**) H4K8ac (green) and PNA (red), see also Supplementary Fig. 5a, b; (**g**) γH2AX (green) and PNA (red). Right panels show monochromatic images of the boxed area in the left panels (**a**, **c**, **e**, **g**). Stages of seminiferous tubule are denoted by Roman numerals. White arrows indicate positive signals; White arrowheads indicate negative signals. DNA was counterstained with DAPI. Scale bars are indicated. A minimum of three animal samples were used for each genotype and each experiment was repeated three times with similar results.

During spermiogenesis, transition proteins (TNPs) replace canonical histones and are subsequently substituted by protamines (PRMs)^2,14^, resulting in a packaged chromatin structure^9,41^. To address the potential cause whereby genetic mutation of *Ddx43* compromises chromatin during spermiogenesis, we embarked on inspection of chromatin remodeling events by co-immunolabeling TNPs and PNA. At stages XII–I, aside from the difference in the morphology of some elongating spermatids, TNPs in testes of all genotypes displayed similar fluorescent localization and intensity on their nuclear heads (Supplementary Fig. 4a, b). At stage VIII, whereas TNPs were normally missing in wild-type step 16 elongated spermatids, both transition protein 1 (TNP1) and 2 (TNP2) were present in partial *Ddx43*^–/–^ and *Ddx43*^KI/KI^ seminiferous lumens (Fig. 3a, b and Supplementary Fig. 6a, b). And, the majority of TNP-positive cells presented irregular rod- or round-like heads (Fig. 3a, b; white arrows), in contrast to TNP-negative cells (Fig. 3a, b; white arrowheads). At the same stage VIII, although protamine 2 (PRM2) in some *Ddx43* mutant elongated spermatids was as intensively visible as wild type (Fig. 3c; white arrows), it was often absent or fragmentary on many others (Fig. 3c; white arrowheads), not as uniformly visible as in nearly all wild-type elongated spermatids (Fig. 3c and Supplementary Fig. 6c). More importantly, some condensed spermatids were retained at stage IX, when the TNP2 signals on the less condensed chromatin were conspicuous (Fig. 3d; white arrows) and those on the more condensed were weak or absent (Fig. 3d; white arrowheads) (Fig. 3d and Supplementary Fig. 6d, e). In parallel, at stage X, the retained spermatids were mainly accompanied with PRM2-positive signals (Fig. 3e, white arrows), and partially negative (Fig. 3e; white arrowheads) (Fig. 3e and Supplementary Fig. 6f). These special phenomena at stages IX–X match well with our aforementioned shot in Fig. 2f. In addition, TNP2 retention was found in the sporadic premature sperm sloughed to *Ddx43*^–/–^ epididymis (Supplementary Fig. 4c). Taken together, these data indicate that DDX43 deficiency impairs TNP-to-PRM substitution, though this transition is not absolutely blocked.

Hyperacetylation of histone H4 takes place just prior to TNP replacement of canonical histones and is thought to be a crucial step for the subsequent events^17,42,43^. In accordance with previous report^5,42^, in wild-type testis, a strong labeling for H4K8ac was confined to step 9-11 elongated spermatids at stages IX–XI (Fig. 3f and Supplementary Fig. 5a). In *Ddx43* mutant testes, while signal intensity of H4K8ac was comparable at stages IX–X (Supplementary Fig. 5b), H4K8ac-positive cells remained there at stages XII–I (Fig. 3f; white arrows and Supplementary Fig. 6g). The partial eviction of H4K8ac in mutant late elongating spermatids prompted us to further examine the phosphorylated H2AX (γH2AX), a marker for transient physiological DNA strand breaks, which is pivotal for the process of nucleosomal DNA supercoils elimination and DNA damage response^7^. In wild-type testis, γH2AX was cleared in spermatids from step 13 (stage I) onward (Fig. 3g). In *Ddx43* mutant testes, γH2AX was still present in step 14 (stage II) spermatids (Fig. 3g; white arrows and Supplementary Fig. 6h). In order to directly assess DNA strand breaks, the sensitive TUNEL assay was conducted^8,44^. As a result, there was a significant increase in TUNEL-positive signals at step 13-16 in *Ddx43*^–/–^ compared with wild-type testis (Supplementary Fig. 5c), implying the retention of γH2AX is likely attributed to uncleared DNA strand breaks. These results suggest DDX43 is required for hyperacetylated H4 eviction and DNA repair during spermiogenesis.

### DDX43 loss does not frustrate meiosis and piRNA biogenesis

Considering the onset of DDX43 expression from spermatocytes as well as the potential defects in DSB repair in *Ddx43* mutant spermatids, DDX43 deficiency might as well impact meiotic events such as DNA double-strand breaks and chromosome synapsis^45^. Hence, we conducted co-immunofluorescence analysis of SYCP3 (a lateral element of the SC complex) and γH2AX on the chromosome spreads of spermatocyte nuclei. To our expectation, γH2AX staining patterns are indistinguishable for each type of spermatocyte between wild-type and mutant testes (Supplementary Fig. 7a). Nor obvious defects were observed via stage-specific comparison of double immunofluorescence for SYCP1 (a central element of the SC complex) and SYCP3 (Supplementary Fig. 7b), combined patterns of which are well-adopted to define each stage of the meiotic prophase I. In concert with the robust production of post-meiotic round spermatids, these results suggest that DDX43 has no major influence on meiotic progression.

DDX43 was recently reported to function as a Vasa-like protein to facilitate amplification of PIWI-interacting RNAs (piRNAs) in an ovary-derived cell line of silkworms^46^. In mammalian testis, pachytene piRNAs, as one predominant class of piRNAs, are generated in pachytene spermatocytes and round spermatids^47,48^. Based on its expression pattern, DDX43 might regulate pachytene piRNA biogenesis in mouse testis. Hence, we radiolabeled total small RNAs and nevertheless showed comparable abundance of pachytene piRNAs in wild-type versus mutant testes (Supplementary Fig. 8a). As expected, immunofluorescence did not show derepression of two major piRNA-associated retrotransposons, long interspersed nuclear element-1 (LINE1) and intracisternal A-particle (IAP) in mutant testes (Supplementary Fig. 8b). MIWI and MILI, two classical piRNA pathway proteins, normally localize to the inter-mitochondrial cement (IMC) of spermatocytes and the chromatoid body (CB) of round spermatids^49,50^. As expected, neither their protein levels (Supplementary Fig. 8c) nor localization patterns (Supplementary Fig. 8d) were affected. Thus, unlike in silkworms, DDX43 seems dispensable for piRNA biogenesis in mice.

### Misled differentiation trajectory induced by *Ddx43* mutation

Next, we performed RNA-seq using wild-type and *Ddx43* mutant testes (three per genotype) collected at P21 and P28, two time points separately enriched for spermatocytes and spermatids. Counting up those expressed in either genotype, there were total 14,972 and 15,812 transcripts at P21 and P28 in *Ddx43*^–/–^, as well as 14,965 and 15,856 in *Ddx43*^KI/KI^, respectively. However, only 3 and 109 expressed genes in *Ddx43*^–/–^ testes, as well as 2 and 115 in *Ddx43*^KI/KI^ testes, were significantly altered when compared with wild type (FDR adjusted *P* value <0.05 and absolute log 2 (fold change) > 1) (Supplementary Fig. 9a). *Ddx43* mutant transcripts were hereby validated by mapping RNA-seq reads to the gene locus as referenced to the wild type (Supplementary Fig. 9b). The limited number of differentially expressed genes above upon deletion of an RNA helicase raised a puzzle what is indeed responsible for DDX43-mediated chromatin remodeling during spermiogenesis. We speculate that because total RNA-seq data reflect rather an ensemble average of different cell types, it may limit a deep deciphering of the highly dynamical regulation in the process of chromatin remodeling.

**Fig. 4:**
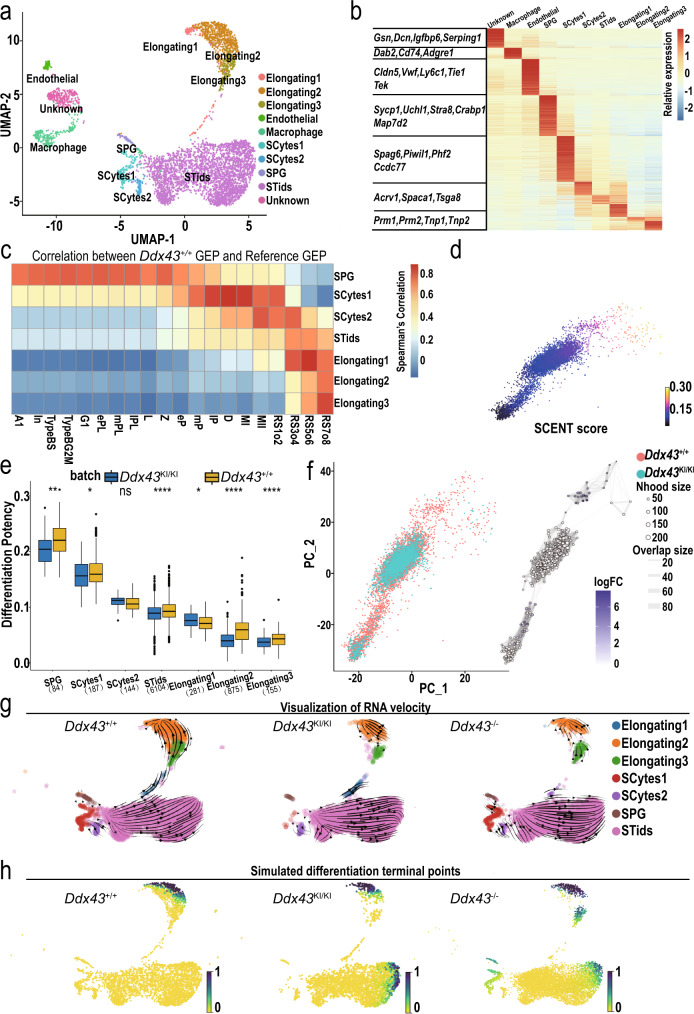
Overview of single-cell transcriptome profiling on adult *Ddx43*^+/+^ and *Ddx43* mutant whole testes. **a** UMAP and clustering analysis of combined single-cell transcriptome from mice testes (*n* = 17133). Each dot represents a single cell and is colored according to its cluster identity as indicated on the figure key. The 10 cluster identities were assigned based on marker gene expression. UMAP: Uniform Manifold Approximation and Projection. SPG, spermatogonia；SCytes, meiotic spermatocytes; STids, post-meiotic haploid round spermatids; ES, elongating spermatids. **b** The heatmap for expression of selected marker genes for the 10 cell types. The color bar denotes the scaled gene expression value. **c** The heatmap of cell type gene expression Spearman correlation between our annotated single cell reference expression profile and flow-sorted single cell gene expression profile. The color bar denotes the correlation coefficients between each annotated cell type in our dataset and reference dataset. A1, type A1 spermatogonia; In, intermediate spermatogonia; BS, S phase type B spermatogonia; BG2, G2/M phase type B spermatogonia; G1, G1 phase preleptotene; ePL, early S phase preleptotene; mPL, middle S phase preleptotene; lPL, late S phase preleptotene; L, leptotene; Z, zygotene; eP, early pachytene; mP, middle pachytene; lP, late pachytene; D, diplotene; MI, metaphase I; MII, metaphase II; RS2, steps 1–2 spermatids; RS4, steps 3–4 spermatids; RS6, steps 5–6 spermatids; RS8, steps 7–8 spermatids. **d** PCA plot of single cell transcriptome data with cells colored based on their cell differentiation potency (network entropy) calculated by SCENT. **e** The boxplot of cell differentiation potency for each differentiation stages during spermatogenesis of both *Ddx43*^+/+^ and *Ddx43*^KI/KI^ mice. Asterisks indicate statistical significance: * denotes *P* value $\le$ 0.05, ** denotes *P* value $\le$ 0.01, **** denotes *P* value $\le$ 0.0001; ns, not significant. Wilcoxon rank-sum two-sided test. In the boxplots, the center line, box limits and whiskers denote the median, upper and lower quartiles and 1.5 × interquartile range, respectively. **f** A neighborhood graph of the results from Milo differential abundance testing (right panel). Nodes are neighborhoods, colored by their log fold change across genotypes. Non-differential abundance neighborhoods (*P* value > 0.05) are colored white, and sizes correspond to the number of cells in each neighborhood. Graph edges depict the number of cells shared between neighborhoods. The layout of nodes is determined by the position of the neighborhood index cell in the PCA (left panel). **g**, **h** Visualization of the RNA velocity analysis results on the UMAP plot (**g**), and predicted terminal points based on velocity directed trajectory (**h**). Cells are colored according to their differentiation terminal probability.

To explore the impact of DDX43 on gene expression at cell-type resolution, we performed 10x Genomics single-cell RNA-seq (scRNA-seq) throughout the complete spermatogenic lineage in testicular cells derived from adult *Ddx43*^+/+^, *Ddx43*^KI/KI^ and *Ddx43*^–/–^ testes and obtained 17,133 quality-controlled single cells (Methods). To assess both the cell typic compositional changes and transcriptional regulation differences, we built a high confidential transcriptome reference manifold based on our wild-type samples (Fig. 4a), and then projected the *Ddx43*^KI/KI^ and *Ddx43*^–/–^ samples on this reference (Supplementary Fig. 10a). Using our recently developed single cell clustering method named as ICAnet^26^, combined with previously described cell-type specific markers^24^, we identified all major germ cell populations covering whole development process, including spermatogonia (SPG), meiotic spermatocytes (SCytes), post-meiotic haploid round spermatids (STids), and elongating spermatids (ES) (Fig. 4a, b).

We also detected three somatic cell compartments including endothelial, macrophage, and an unknown somatic cell type (Fig. 4a, b) wherein GO enrichment analysis (Supplementary Fig. 10b) of highly expressed marker genes (Supplementary Fig. 10c) showed consistency with a previous study^24^. To examine our cell type identification, we compared our results with previous fluorescence-activated cell sorting (FACS) based smart-seq2 scRNA-seq data^19^, and the correlation heatmap supported the accuracy of our annotations (Fig. 4c). We also noticed that SCytes2 acted as the transition state between SCytes and STids. To further examine our annotation, we quantified the metagene expression of differential expression gene sets between MII and the early stage of STids (up-regulated genes in MII and up-regulated genes in RS1o2) in the cells of SCytes2 and STids using AUCell^51^. As expected, in general, the MII metagene has higher expression value in SCytes2 (Supplementary Fig. 10d).

On this basis, we further calculated each cell type’s differentiation entropy^52^, which is regarded as one of the best characterization metrics for cell differentiation potency, and noticed that the differentiation entropy showed perfectly gradient descent along the differentiation trajectory (Fig. 4d; SPG → SCytes → STids → ES). Collectively, above in-depth computational analyses support the accuracy of our cell type annotation and differentiation trajectory, which means we have an ideal latent space to capture the majority transcriptional regulation information during the spermatogenesis so that we could address the perturbation effects posed by *Ddx43* mutation at cell-type and/or -state resolution.

After building the wild-type reference of mouse spermatogenesis process, we mapped the query cells from *Ddx43*^KI/KI^ and *Ddx43*^–/–^ samples on the reference by an anchor-based integration methods^53^, and transferred the reference label to each query cell (Methods). Both correlation patterns between global gene expression and flow-sorted experimental data supported the accuracy of our annotation on query cells (Supplementary Fig.10e, f). We also applied the metagene analysis in query cells to support the correct annotation of transition cell type SCytes2 (Supplementary Fig. 10g, h).

One of the critical tasks of scRNA-seq is to shed light on the abnormal trajectory events in perturbated sample, which makes the cell states deviate from normal. We calculated the differentiation potency of mapped mutant cells, and noticed that there exists clearly reduced differentiation capacity starting from the stage of SCytes to ES in *Ddx43*^KI/KI^ (Fig. 4e) and *Ddx43*^–/–^ testes (Supplementary Fig. 10i). This deficiency was identified through evaluating expression pattern of each cell, and may also reflect on the manifolds as the population density of cell type changes during the conversion of each type. We next used a recently developed differential abundance testing method^54^ to visualize the cell population density perturbation on the sampled local region of cell k-nearest neighbored (kNN) graph, and identified differential abundant neighborhoods from the stage SCytes2 to STids, and the stage STids to ES (Fig. 4f and Supplementary Fig. 10j), indicating that STids was the crucial cell type/stage relevant to the abnormal cell differentiation in *Ddx43* mutant testes.

A further question is whether *Ddx43* deficiency changes the differentiation trajectory of spermatogenic cells. We applied scVelo, a model that evaluates RNA splicing velocity by fitting the chemical master equation function through Expectation-Maximization (EM) algorithm to derive a highly dimensional vector predictive of future transcriptional state of individual cells^27^, to infer developmental trajectory of spermatogenesis based on scRNA-seq data^55^. Notably, *Ddx43* deficiency changed the direction of the velocity vector within the STids. Unlike wild-type, *Ddx43* mutant STids showed weak progression towards ES (pointed by most arrows) (Fig. 4g). To make a clearer visualization of the perturbation effects, we built a Markov chain and used velocity vector information as the orientation of transition matrix, so that we could predict the terminal differentiation points on the manifold that is reflected by a terminal probability of each cell. Interestingly, in wild-type testes, the cells could safely differentiate to the final stage, ES, while in *Ddx43*^KI/KI^ and *Ddx43*^–/–^ testes, some cells were trapped in the stage of STids (Fig. 4h), in line with our phenotypic observations (Fig. 2f, g). Taken together, these analyses imply that *Ddx43* deficiency misled the differentiation path of STids.

### DDX43 orchestrates RNA regulatory dynamics in early-stage STids

**Fig. 5:**
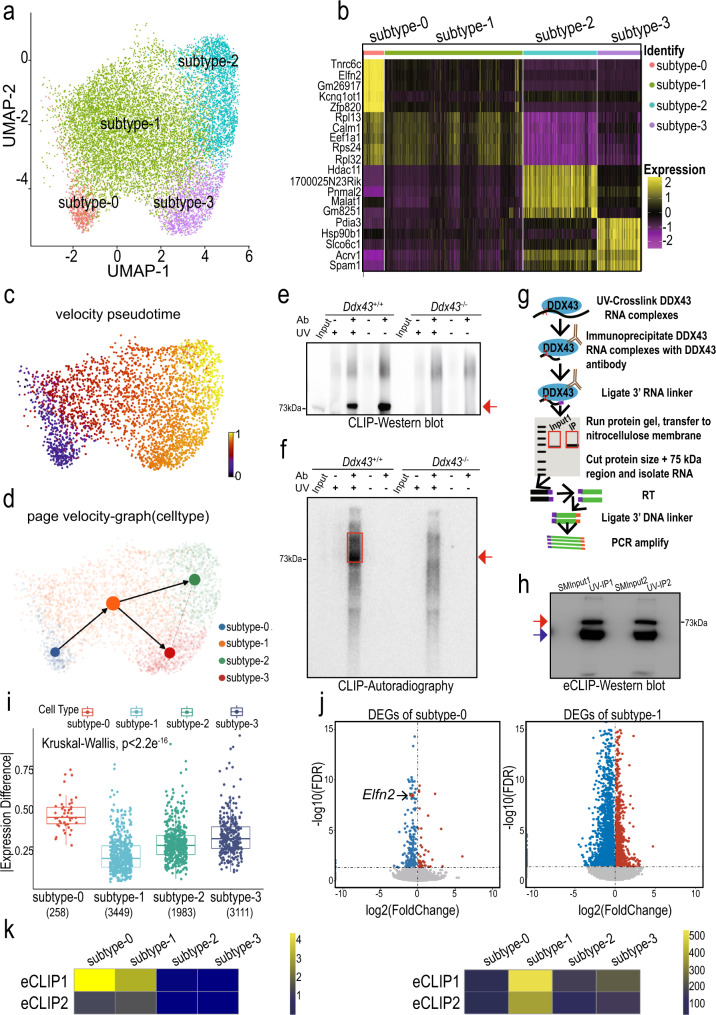
Identification of four transcriptional states for STids with scRNA-seq and eCLIP-seq. **a** Zoom-in analysis of STids reveals four cellular states (subtype-0 to subtype-3) during spermatid development. **b** Top 5 highly expressed genes for each subtype in STids. **c** Velocity pseudotime analysis on STids cells. Each cell is colored according to its velocity time. **d** PAGE directed graph of four states. **e**, **f** Western blot (**e**) and Autoradiography (**f**) analyses of DDX43-RNA complexes prepared by P^32^-radioactive conventional CLIP in P24 testes. Red arrow and red box indicate the DDX43 protein and DDX43-bound RNA signals, respectively. Both non-crosslinked and *Ddx43*^–/–^ samples served as negative controls. These experiments were repeated three times with similar results. **g** Schematic of enhanced CLIP (eCLIP) protocol. See the Methods section for details. **h** Western blot validation of DDX43-RNA complexes prepared by eCLIP in P24 wild-type testes. SMInput1 and SMInput2 served as negative controls in two biological replicates, respectively. Red and blue arrows point to DDX43 protein and heavy chain, respectively. This experiment was repeated three times with similar results. **i** The boxplot for absolute expression difference value of DDX43 differential expression targets (DETs) in four cellular states. **j** Volcano plot showing gene differential expression (*Ddx43*^+/+^ versus *Ddx43*^KI/KI^) of subtype-0 and subtype-1. **k** The heatmap of DDX43 targets enrichments in down-regulated genes of two eCLIP-seq replicates. The heatmap is colored according to the -log10 (*P* value). We used one-sided Fisher-exact test to calculate statistical significance of enrichments (left panel). The heatmap of the number of DETs in two eCLIP-seq replicates (right panel).

Encouraged from the above analyses, we zoomed in the STids for re-clustering after using two cell clustering metrics aid to optimize our clustering procedure (Methods, Supplementary Fig. 11a), and found four subtypes (Fig. 5a). To gain insights into the gene expression dynamics along these four clusters, we analyzed the gene ontology (GO) of subtype-enriched expression genes (Fig. 5b and Supplementary Fig. 11b). These GO terms were in accordance with previous studies about the serial differentiation in mouse male germ cells^19,24^. To connect the four subtypes, we ordered cells in a pseudo-temporal manner using PAGA^56^ and RNA velocity^27^, and noticed the differentiation direction starts from subtype-0 towards subtype-2 (Fig. 5c, d).

To determine the key perturbated phase in STids, we conducted differential expression gene (DEG) analysis between mutant and wild-type samples across four sub-clusters. Using correlation of Fold Changes as the metric to evaluate the DEG consistence of two replicates (Methods), we found the two replicates of *Ddx43*^KI/KI^ showed better consistency compared with *Ddx43*^–/–^ (Supplementary Fig. 12a). Therefore, we zoomed the perturbation effects of *Ddx43*^KI/KI^ samples. DEGs (FDR adjusted *P* value <0.05) in *Ddx43*^KI/KI^ versus wild type showed varying GO enrichments in the four subtypes (Supplementary Fig. 12b).

Next, we asked which subtype may play a crucial role in regulating the differentiation process of STids. To answer this question, we attempted to detect RNA directly bound by DDX43 in vivo. We radiolabeled DDX43-bound RNA after UV-crosslinking immunoprecipitation (CLIP) in P24 testis lysates. Western blot analysis and autoradiography clearly showed CLIP enrichment of DDX43 protein and in parallel its size-matched RNA in wild-type rather than *Ddx43*^–/–^ testes (Fig. 5e, f), supporting that our antibody could efficiently capture crosslinked DDX43-RNA ribonucleoproteins after stringent washes. To achieve a robust profiling of DDX43-bound RNAs at a transcriptome-wide level, we applied enhanced CLIP (eCLIP) as before^57,58^(Fig. 5g). Western blot analysis confirmed eCLIP enrichment of DDX43 protein (Fig. 5h). ‘Size-matched input’ (SMInput) served as control libraries to reduce nonspecific background from eCLIP libraries.

We processed DDX43 eCLIP reads and mapped them using an established pipeline^57^ (Supplementary Fig. 13a), exhibiting high correlation between two biological replicates (Supplementary Fig. 13b). High reproducibility of our eCLIP experiments was manifest with the considerable overlap of significantly enriched peaks from the two replicates (Supplementary Fig. 13c). After filtering those clusters according to their binding affinity to realize quality control, we noticed DDX43 displayed higher enrichments on the untranslated regions (5′ UTR and 3′ UTR) (Supplementary Fig. 13d, e).

Finally, we overlapped DDX43-bound targets with the DEGs (FDR adjusted *P* value < 0.05) for each subtype of *Ddx43*^KI/KI^ sample, and denoted those genes as differential expression targets (DETs). Interestingly, we found the DETs in subtype-0 showed much higher expression difference (abs(avg(knock-in sample)—avg(wildtype))) than other subtypes (Fig. 5i). Consistently, we also found that the DETs in subtype-0 of *Ddx43*^–/–^ sample showed relatively higher expression difference (Supplementary Fig. 14a). Meanwhile, subtype-1 owned the vastest number of DETs (Fig. 5i, k). Furthermore, we discovered that most of DEGs in the subtype-0 and subtype-1 were down-regulated (Fig. 5j, 82.8% in subtype-0 and 68.7% in subtype-1). Consistently, most of DEGs in subtype-1 of *Ddx43*^–/–^ sample were also downregulated (Supplementary Fig. 14b). In addition, the binding targets of DDX43 showed significant enrichments (*P* value <0.001, Fisher exact test) in those downregulated genes of subtype-0 and −1 (Fig. 5k). These results point to a central role of DDX43 at the early stage of STids.

### *Elfn2* is identified as a hub gene regulated by DDX43

**Fig. 6:**
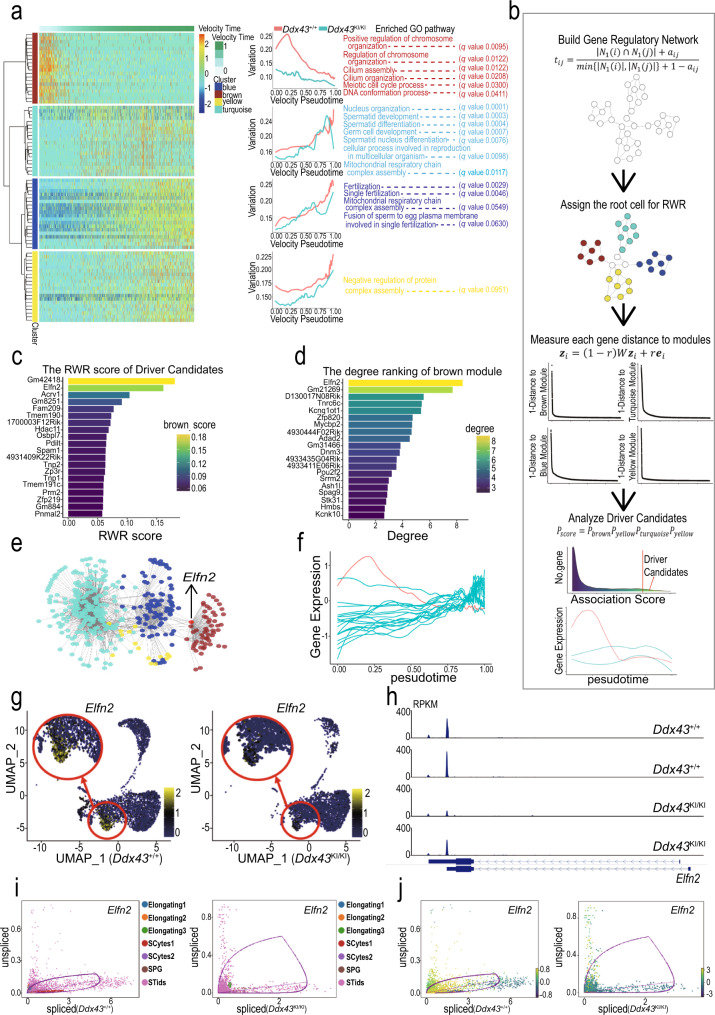
Dynamic network analyses for identifying DDX43-regulated driver genes. **a** WGCNA clustering of genes exhibiting down-regulated expression in subtype-0 and subtype-1. Each row represents a gene, and each column represents a single cell, with columns/cells placed in velocity pseudotime order and depicted by a thick colored line (top). Gene expression levels utilize a Z score transformation. **b** A workflow of page ranking algorithm to identify regulation driver candidates. In step 1, we converted the topological overlap matrix to Markov transition matrix through row normalization. In step 2, we assigned the diffusion root through regarding those cells belong to gene co-expression module as the seed cells. In step 3, we performed message passing and got four rank score, each measure the proximity of each gene to the module. In final step, we aggregated those scores and identified the overall highly ranked genes as the driver candidates. **c** Barplot of association rank of driver candidates to the brown module. Each bar is colored according to each gene topological associate score to brown module. The score is calculated from random walk with restart algorithm in (**b**). **d** Barplot of the network connectivity of driver candidates. Each bar is colored according to each gene connectivity in the network, which reflects the gene’s importance to the biological systems. **e** The gene regulatory network related to the dysregulation in subtype-0 and subtype-1. Each node represents a gene colored according to the module it belongs to. **f** Expression levels of driver candidate genes during spermatid development. The *x*-axis represents velocity pseudotime, and the y-axis represents gene expression levels. The expression curve of *Elfn2* is marked in red. **g** Expression patterns of *Elfn2* in *Ddx43*^+/+^ and *Ddx43*^KI/KI^ spermatogenic cells, with their expression projected onto the UMAP plot. **h** The track plot of *Elfn2* in *Ddx43*^+/+^ and *Ddx43*^KI/KI^ subtype-0 cells, in two paired replicates. **i**, **j** The gene life cycle of *Elfn2* in *Ddx43*^+/+^ and *Ddx43*^KI/KI^ cells. Each dot represents a cell colored according to the cell types (**i**) or velocity values (**j**). The *x*-axis represents the spliced RNA expression level; the *y*-axis represents the unspliced RNA expression level.

We proceeded to elucidate the molecular dynamical network related to the DDX43 regulation in the early stage of STids. We analyzed the gene expression covariation of the down-regulated DEGs in subtype-0 and −1 from *Ddx43*^KI/KI^ samples through WGCNA (weighted gene co-expression network analysis)^59,60^, and observed four major categories of transcriptional gene modules in characterized patterns (Fig. 6a). We also quantified those modules activity by AUCell and fitted a continuous curve alongside the velocity pseudo-time with local polynomial regression. We noticed most of the modules were gradually upregulated in activity and were largely involved the biological processes corresponding to the functions at the later stage of STids, such as nucleus organization, fertilization, and mitochondrial respiratory chain complex assembly (Fig. 6a), indicating DDX43 regulates genes highly expressed in later-stage modules at early stage.

Notably, there existed a co-expression module (denoted as brown module) strongly altered at early stage, with the activity consistently inhibited in *Ddx43*^KI/KI^ cells (Fig. 6a). The genes in this module were largely involved with chromosome organization and DNA conformation processes. Consistently, the WGCNA results using the down-regulated genes of subtype-1 in *Ddx43*^–/–^ samples also showed a co-expression module (denoted as green module) strongly altered at early stage, with the activity also inhibited in *Ddx43*^–/–^ cells (Supplementary Fig. 14c, d). This module also enriched with the pathways like DNA conformation, histone modification, and chromatin remodeling (Supplementary Fig. 14e). Combining the multiple dysfunctions in H4K8ac eviction (Fig. 3f), DNA repair (Fig. 3g and Supplementary Fig. 5c), and TNP-to-PRM transition (Fig. 3a–e) and activation of the epigenetic regulation-associated modules in the early-stage STids (Fig. 6a and Supplementary Fig. 14c–e), we hypothesized regulation of the co-expression modules may act as the origin events during spermatid differentiation.

We thus attempted to verify this assumption. To investigate the core genes related to the dysfunction of the brown module in *Ddx43*^KI/KI^ sample, we used page ranking algorithms on weighted gene-gene co-expression network, coupled with their topological distance to the four modules, to identify the top genes that proximal to the four modules (Fig. 6b). We noticed some genes of the top-gene list are associated with spermiogenesis (Fig. 6c, d), such as *Acrv1* (an acrosomal matrix protein) and three core genes *Tnp1*, *Tnp2* and *Prm2* engaged in TNP-to-PRM transition. As these genes are not likely the regulatory origin of *Ddx43*^KI/KI^ defects, we paid attention to another one, *Elfn2*, known as an epigenetic regulator highly expressed in the nervous system and testis^61^.

*Elfn2* is not only ranked as the top 2nd driver candidate belonging to the ‘brown module’ (Fig. 6c), but also identified as one hub gene which owns the highest connectivity compared with other partner genes in the ‘brown module’ (Fig. 6d, e). *Elfn2* is also identified as the top hub gene in the green module in *Ddx43*^–/–^ sample (Supplementary Fig. 14f). Moreover, *Elfn2* is expressed at the early stage of spermatids just later than *Ddx43* (Fig. 6f and Supplementary Fig. 15a), and is the top down-regulated DET in DDX43 mutants (Supplementary Fig. 15b). The gene expression analysis also determined the dysregulation of *Elfn2* in the early-stage STids in *Ddx43*^KI/KI^ and *Ddx43*^–/–^ testes (Fig. 5j, Fig. 6g, and Supplementary Fig. 14g). In addition, the track plot further confirmed the existing of *Elfn2* transcript in subtype-0 STids (Fig. 6h). Gene spliced-unspliced life cycle analysis also showed that *Elfn2* tends to have higher ratio of unspliced transcripts in *Ddx43*^KI/KI^ testis (Fig. 6i, j and Supplementary Fig. 15c, d). Our integrated data analyses suggest that the downregulation of the hub gene *Elfn2* may lead to the imbalance of the ‘brown module’ network.

### *Elfn2* knockdown recapitulates deficiencies in *Ddx43* mutants

**Fig. 7:**
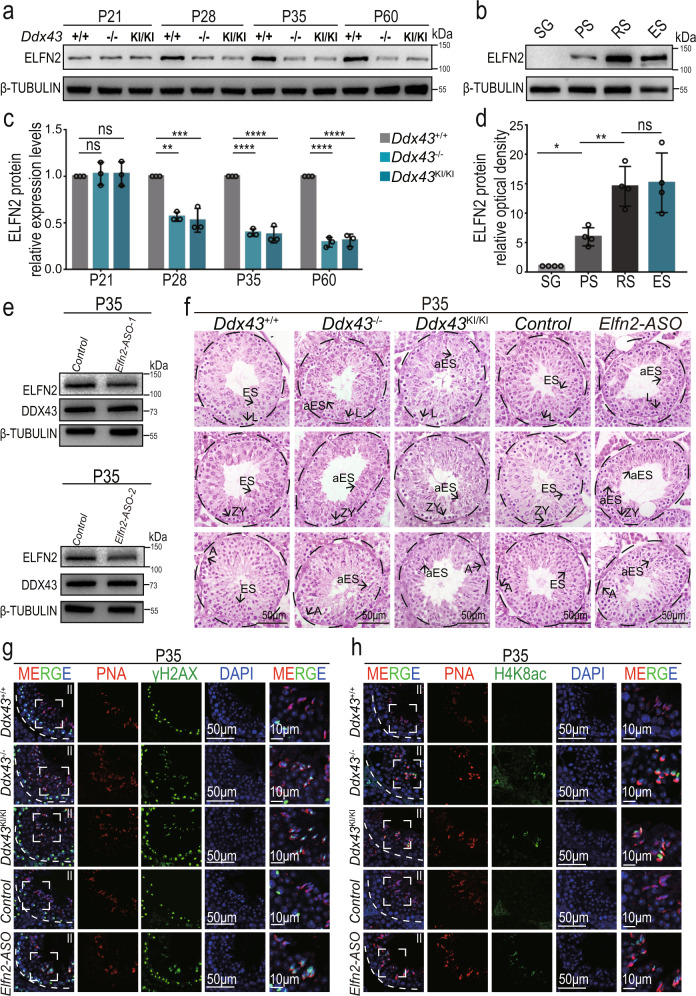
*Elfn2* knockdown in mouse testis reproduces similar spermatid defects in *Ddx43* mutants. **a**, **b** Western blot analyses of ELFN2 protein in extracts from wild-type and two *Ddx43* mutant testes at indicated postnatal time points (**a**) and spermatogenic cells purified from wild-type mice (**b**), including spermatogonia (SG), pachytene spermatocytes (PS), round spermatids (RS), and elongating spermatids (ES). β-TUBULIN serves as a loading control. **c**, **d** Quantification of the ELFN2 protein levels in (**a**) and (**b**). The expression levels of ELFN2 are normalized to β-TUBULIN. The relative ELFN2 expression levels in *Ddx43*^+/+^ testes (**a**) and SG (**b**) are set at 1.0. *P* values were calculated by oneway ANOVA. ns, not significant; *P* = 0.9438 (*Ddx43*^+/+^ vs *Ddx43*^–/–^ at P21), *P* = 0.9474 (*Ddx43*^+/+^ vs *Ddx43*^KI/KI^ at P21), **P = 0.0013 (*Ddx43*^+/+^ vs *Ddx43*^–/–^ at P28), ***P = 0.0008 (*Ddx43*^+/+^ vs *Ddx43*^KI/KI^ at P28), **** *P* < 0.0001, *P = 0.0226 (SG vs PS), ** *P* = 0.008 (PS vs RS), *P* = 0.8982 (RS vs ES). Data presented are mean ± SD from three independent experiments. **e** Western blot analyses confirmed that ELFN2 protein was efficiently knocked down in *Elfn2* knockdown mouse lysates. β-TUBULIN serves as a loading control. **f** H&E staining analyses using P35 testis sections prepared from *Ddx43*^+/+^, *Ddx43*^KI/KI^, *Ddx43*^–/–^, *Elfn2* control and ASO-transduced P35 testes. Scale bars are indicated. A, type A spermatogonia; L, leptotene spermatocyte; ZY, zygotene spermatocyte; ES, elongating spermatid; aES, abnormal elongating spermatid. Results shown are representative of a minimum of three animal samples used for each genotype. **g**, **h** Immunofluorescence analyses using P35 testis sections of indicated genotypes with two combinations: γH2AX (green) and PNA (red) (**g**); H4K8ac (green) and PNA (red) (**h**). The rightmost panels show magnified images of the boxed area in the left panels. DNA was counterstained with DAPI. Stage numbers and scale bars are indicated. Each experiment was repeated three times with similar results.

To directly assess whether endogenous *Elfn2* contributes to chromatin remodeling during spermiogenesis, we first examined its testicular protein levels at four postnatal time points. Early at P21, concomitant with the appearance of round spermatids, ELFN2 protein abundance was equally low in wild-type versus *Ddx43*^KI/KI^ and *Ddx43*^–/–^ mutant testes (Fig. 7a, c). At P28, P35 and P60, when round spermatids accumulated, ELFN2 protein levels increased but were significantly decreased in mutant testes, compared with wild type (Fig. 7a, c). These results are in concert with our scRNA-seq analytical data showing downregulation of *Elfn2* mRNA in *Ddx43* mutants, as well as our observation of the phenotypic defects emerging from P28 (Supplementary Fig. 2e). In tune with their different timings for attaining peak mRNA levels (Supplementary Fig. 15a), ELFN2 protein is most abundant in spermatids (Fig. 7b, d), later than DDX43 (Fig. 1c).

To elucidate the in vivo function of *Elfn2*, we knocked down *Elfn2* through testis microinjection of antisense oligonucleotides (ASOs)^62^. We validated knockdown efficiency in P35 testes 11 days after ASO transduction to P24 testes by Western blot analysis. ELFN2 protein levels declined in ASO knockdown versus control (Fig. 7e), proving the validity of our *Elfn2* knockdown model. Histological analysis of P35 testes revealed phenotypic defects in *Elfn2* knockdown seminiferous tubules, similar to *Ddx43* mutants (Fig. 7f). Meanwhile, we co-immunolabeled γH2AX and PNA, and observed strong γH2AX localization on step 14 spermatids in both *Ddx43* mutant and *Elfn2* knockdown seminiferous tubules at stage II, but not in *Ddx43*^+/+^ and *Elfn2* controls (Fig. 7g). In parallel, co-immunolabeling H4K8ac and PNA showed the presence of H4K8ac-positive step 14 spermatids only in *Ddx43* mutant and *Elfn2* knockdown testes (Fig. 7h). These deficiencies displaying abnormally remained γH2AX and H4K8ac in P35 testes resemble those described in P60 testes (Fig. 3f, g), suggesting *Elfn2* is at least partly responsible for DDX43-orchestrated chromatin remodeling in spermiogenesis.

### DDX43 acts on *Elfn2* 5′ UTR and binds RNA G-quadruplex

To address how DDX43 regulates *Elfn2* expression, after affirming there is no endogenous expression of DDX43 in HEK293 cells (Supplementary Fig. 16a), we employed dual luciferase reporter assay in HEK293 cells, where we introduced *Elfn2* 5′ UTR into the reporter vector and overexpressed exogenous DDX43 proteins (-WT, -KO, -KI) fused with FLAG tag (Supplementary Fig. 16b). As a result, FLAG-DDX43-WT and psi-*Elfn2*−5′ UTR co-transfected cells possess a higher relative luciferase activity than those transfected with FLAG-DDX43-KO or -KI (Supplementary Fig. 16c), suggesting that DDX43 acts positively upon *Elfn2* 5′ UTR facilitating the reporter gene translation. Compared with DDX43-KO protein being undetected in HEK293 cells, both DDX43-WT and -KI proteins were overexpressed well (Supplementary Fig. 16d, e), and enriched with *Elfn2* 5' UTR transcript by immunoprecipitation (Supplementary Fig. 16f, g).

To probe the potential sequence-binding preference of DDX43, we first calculated the motif enrichments within the top 50% (ranking the binding cite according to the FDR adjusted *P* value) of the DDX43-binding sites from all eCLIP peaks. We found that DDX43 has a remarkable propensity of binding G-rich sequences (Supplementary Fig. 17a). Analysis of G-quadruplex (G4) score further showed that DDX43-bound regions have a significantly higher G4 propensity than DDX43-bound flanking regions (Supplementary Fig. 17b). For validation, we purified DDX43 and DDX43-KI proteins (Supplementary Fig. 17c, d), and performed EMSA (electrophoretic mobility shift assay) to test their binding affinity on one generic sequence of RNA G4 (RG4) as well as a single-stranded RNA (ssRNA)^63^, followed by using two different G-rich sequences identified within *Elfn2* 5′ UTR that are in compliance with the general RG4 pattern^64^. Our results show both DDX43 and DDX43-KI proteins bind these RG4 sequences rather than the ssRNA (Supplementary Fig. 17e–g), suggesting that DDX43 may interact with the RG4 loci in *Elfn2* 5′ UTR.

## Discussion

It is well documented that post-meiotic germ cells undergo chromatin remodeling involving multiple intricated events that are chronologically coordinated, accompanied by progressive compaction of sperm nucleus. scRNA-seq has provided delicate new insights into how spermatid development experiences a highly dynamic process of transcriptome transformations. Thereupon, it is tempting to ask whether there exists a factor that bridges the regulation of developmentally dynamic transcriptome and shaping of chromatin architecture during spermiogenesis. Here, we show that genetic mutation of *Ddx43* leads to defects in developing spermatids, failure to produce mature spermatozoa, and male infertility. Importantly, *Ddx43*-deficient spermatids exhibit aberrant occurrence of stage-dependent events of chromatin remodeling. The reasons that these phenotypic and molecular outcomes stem from an unprecise control of gene expression are two-fold. First, despite that partial mutant spermatids may be developmentally retarded or not purged from prior stages, the overall heterogeneity in molecular defects present at same steps precludes a fully uniform arrest unreachable to specific cell types with target gene expression. Second, scRNA-seq unravels the dysregulation of a group of enriched and related genes in mutant testes, and therein identified *Elfn2* as a targeted hub gene downstream of DDX43 in early-step spermatids. Therefore, our work demonstrates an important role of DDX43 and highlights its stage-specific transcriptomic control of chromatin remodeling.

To seek the molecular function of DDX43 in vivo, we explored three possibilities before employing scRNA-seq. First, we created gene-edited mice bearing a point mutation in the ATP hydrolysis site of DDX43. This site is highly conserved and validated for functional importance^39,40,65^. However, DDX43 protein itself is almost absent in this KI testis, rendering it improbable to use the KI model for helicase-directed mechanistic exploration. Similar point mutation-elicited self-exhaustion is  reported for other proteins^66,67^. Second, we tested whether loss of DDX43 affects the piRNA pathway, but found no evident clue. We previously demonstrated another testis-specific RNA helicase MOV10L1 governs piRNA biogenesis using KO mouse models^34,50^. As such, two different RNA helicases herein with overlapping expression are not committed to a joint pathway. Third, to study DDX43 as an RNA regulator, we performed RNA-seq analysis using postnatal testes enriched for certain types of germ cells. Unexpectedly, *Ddx43* deficiency does not cause a major change in gene expression. Some other studies for chromatin remodelers in spermatids also show limited power of the bulk RNA-seq in revealing an overall transcriptomic alteration^5,15^. The challenges posed by all above negative results represent opportunities to envision an unexpected mode of action of DDX43 in testis. As a scenario, within a certain narrow developmental window may DDX43 control the abundance of determinant genes, whose expression fluctuations are nevertheless unable to be manifested into significant values for RNA-seq.

We thus proceeded to subject *Ddx43* mutant models to scRNA-seq to deeply analyze transcriptome pattern-specified populations of wild-type versus mutant germ cells. We fed ICAnet into *Ddx43* mutant models and generated an atlas of DDX43-regulated transcriptomes with full coverage of spermatogenic cell types. To infer intrinsic developmental trajectory of dissected germline subpopulations, we adapted scVelo into scRNA-seq analytic pipeline. Using velocity-directed Markov chain simulation to predict the differentiation terminal probability along the spermatogenesis, we observed that RNA velocity of wild-type germ cells showed a strong directional flow towards the termini. However, mutant germ cells exhibited a new differentiation terminal point, prompting an in-depth analysis of the transcriptomic perturbation in spermatids. Our study has exemplified a paradigm where RNA velocity can be used to depict gene-regulated dynamic processes in spermatogenesis, and will facilitate trajectory inference of germ cells under various genetical contexts.

Although scRNA-seq can help identify key subtypes of cells and dysregulated genes in any given subtype, it is inadequate to determine the initially affected subtype and the changed genes that are likely to be “causative”. Remarkably, the eCLIP targets of DDX43 were significantly enriched in those downregulated genes in the subtype-0 and -1, supporting our prediction that the early spermatids may be the initial populations controlled by DDX43. The algorithm WGCNA enables us to screen gene network for identifying candidate hub gene from clusters constituted by highly correlated genes^60^. We found that the co-expression module strongly altered at the early stages, and the genes function in this module happened to be echoed with the observed dysfunctions of chromatin remodeling in *Ddx43* mutants. We further applied page ranking algorithms on weighted gene-gene co-expression network and inferred that *Elfn2* may be the key target of DDX43 and function in spermiogenesis. This inference was validated by the follow-up experiments showing that the *Elfn2* knockdown spermatids exhibit mutant-comparable defects. These combined analyses have therefore enabled effective identification of novel gene expression modules, and more importantly, key target genes that are regulated by an RNA-binding protein. Coupling eCLIP-seq that is specialized for RNA-protein interactions in testis with scRNA-seq that unveils regulation of target modules would be an impetus towards ultimately bringing them together at single-cell scales.

Currently, scRNA-seq is being utilized to delineate cellular and molecular processes with response to various biological or environmental perturbations. As a unique study presented herein is our scRNA-seq investigation of single-gene-governed spermatogenesis. Our study defines an unusual RNA regulatory pathway in leading the progressive differentiation trajectory of germ cells. As a framework, scRNA-seq integrated with analytical methodologies, genetic models and molecular approaches will dramatically elevate our ability to resolve the highly ordered dynamics regulation of spermatogenesis.

## Methods

### Animals

*Ddx43* gene is located on mouse chromosome 9 and comprises 17 exons (Fig. 1g). For generating knockout (KO) mice, we designed two guide RNAs (gRNA) targeted exon 4. Briefly, sgRNAs sequences were cloned into pUC57-sgRNA vector (Addgene 51132). sgRNAs were transcribed using the MEGA shortscript T7 Transcription Kit (Life technologies, AM1354) and purified with RNeasy Mini Kit (Qiagen, 74104). sgRNAs (5 ng/μl each) and Cas9 mRNA (20 ng/μl, Thermo Fischer Scientific, A29378) were introduced into C57BL/6J mouse zygotes by electroporation. Founder mice were selected according to Sanger sequencing results and crossed with wild-type C57Bl6/J (Jackson) partners to obtain germline transmission. Mice were selected at random and at least three animal samples were used in each experiment. Using analogous methods, Cas9 mRNA, sgRNA and donor ssDNA mix were injected into C57BL/6J mouse zygotes. And then we obtained knock-in (KI) mice with a missense mutation (DEAD-DQAD) in the DDX43 ATPase motif.

### Research ethics

All animals were handled as stipulated by the Guide for the Care and Use of Laboratory Animals at Nanjing Medical University (ID: IACUC-1808007 and IACUC-1704015), and raised in a suitable environment with moderate light, clean water and food.

### Antibodies

#### Commercial antibodies

The following antibodies were purchased: anti-SYCP3 (Abcam, ab97672, IF: 1:100), anti-SYCP3 (Abcam, ab15093, IF: 1:100), anti-SYCP1 (Abcam, ab15090, IF: 1:100), anti-γH2AX (MerckMillipore, 16-202A, IF: 1:800), anti-TNP2 (Santa Cruz, sc-393843, IF: 1:50), anti-PRM2 (Briar Patch Biosciences, Hup 2B, IF: 1:300), anti-H4K8ac (ABclonal, A7258, IF: 1:500), anti-ELFN2 (Novus Biologicals, 90569, WB: 1: 1000), anti-H3 (Abcam, ab1791, WB: 1: 2000), anti-GAPDH (Abcam, ab8245, WB: 1: 10000), anti-TUBLIN (Sigma, T8328, WB: 1: 2000), anti-FLAG (Sigma, F3165, IP: 10 µg, WB: 1:2000). For immunofluorescence studies, the following secondary antibodies were used: anti-rabbit (Jackson ImmunoResearch 488, 711225152, IF: 1:350), anti-rabbit (Jackson ImmunoResearch 594, 711585152, IF: 1:350), anti-mouse (Jackson ImmunoResearch 488, 715545150, IF: 1:350). For Western blot analyses, the following secondary antibodies conjugated to Horse Radish Peroxidase were used: anti-rabbit IgG HRP-linked (ABclonal, AS014, WB: 1:5000), anti-mouse IgG HRP-linked (ABclonal, AS003, WB: 1:5000).

#### Other antibodies

Anti-ACRV1 antibody is a gift from Eugene Yujun Xu (Nanjing Medical University). Anti-IAP antibody is a gift from B. R. Cullen (Duke University Medical Center, Durham, NC). Anti-L1 ORF1p antibody is a gift from Dónal O’Carroll (European Molecular Biology Laboratory, Italy). Custom-made DDX43 antibody is provided by ABclonal. Briefly, a cDNA fragment of *Ddx43* corresponding to amino acids 630-646 (EMEKKMGRPQGKPQKFY) was cloned into the pGEX-4T-1 vector. Using glutathione-sepharose beads, the GST-DDX43 (630-646) fusion protein was purified from Rosetta bacteria. Purified recombinant DDX43 protein was next administered to two rabbits. DDX43 antiserum was collected and affinity-purified. Finally, we proved the effectiveness of the purified DDX43 antibody in Western blot (1:1000), immunostaining (1:500), and CLIP experiments (10 µg).

### Western blot

Testis tissues were collected, quickly washed twice in cold PBS, and then homogenized with a glass tissue homogenizer in lysis buffer (50 mM Tris pH 7.5, 150 mM NaCl, 1% NP-40, 0.5% sodium deoxycholate) containing protease inhibitors (Roche, 11697498001). Supernatant fractions were collected by centrifugation and boiled with 2× Laemmli sample buffer (Bio-Rad, 1610737) at 95 °C for 8-10 min. The proteins were separated by Sodium Dodecyl Sulfate-Polyacrylamide Gel Electrophoresis (SDS-PAGE, 10% acrylamide running gel), and then electrically transferred from Gel to 0.45 µm polyvinylidene fluoride (PVDF) membranes (Bio-Rad, 1620184) at 90 V for 30 min, plus 120 V for 90 min. After washing membranes with Tris-buffered saline (TBS, 20 mM Tris, 150 mM NaCl, pH 7.6), membranes were blocked with 5% skimmed milk in TBST buffer (TBS with 0.05% Tween20) at room temperature for 1 h and then incubated with antibodies overnight. The next day, membranes were washed with TBST buffer for 5 min and incubated with HRP conjugated secondary antibodies at room temperature for 1 h. Signals were visualized by an enhanced chemiluminescence detection system (Tanon-5200), and analyzed by Image J (NIH).

### Quantitative and semi-quantitative RT-PCR

Total RNA was extracted from tissues or cells with Trizol reagent (Thermo Fisher Scientific, 15596026) and DNaseI (Amp grade 1.5 µ/µl, Invitrogen RNase free). Prime Script RT Master Mix (Takara, RR036A) was used to reverse transcribe 1 µg of total RNA into cDNA. SYBR Green Premix Ex Taq II (Takara, RR820A) was used to analyze the quantitative RT-PCR (qRT-PCR) with 2 µl diluted cDNA as template. *Rplp0* (also known as *36b4*) served as internal control. For semi-quantitative RT-PCR, 1 µl diluted cDNA was used as the template for each reaction with Emerald Amp GT PCR Master Mix (Takara, RR310). After appropriate amplification cycles, half of the reaction products were analyzed by 1% agarose gel electrophoresis. *Actb* and *ACTB* were used as a loading control. All primer sequences are listed in Supplementary Data 1. Images were acquired using Tanon 2500 Fully automatic gel imaging system, and qRT-PCR was performed in Thermofisher Step One Plus Real-Time PCR System.

### Histology and transmission electron microscopy

To prepare paraffin sections, testes, and epididymis were fixed in 5 ml Bouin’s solution (Sigma, HT10132) overnight. The next day, tissues were dehydrated with a graded series of ethanol (70%, 2 h; 80%, 2 h; 95%, 2 h; 100%, 2 h), soaked the tissues in xylene (Biosystems) for 3 h, replaced by paraffin for another incubation at 65 °C overnight, and then embedded into plastic molds with paraffin. Samples were sliced into 5 µm by microtome (Leica, RM2135). The sections were dried at 60 °C and then stored at room temperature. For histological analyses, the sections were deparaffinized, rehydrated, and then stained with Hematoxylin and Eosin (H&E, Sigma-Aldrich) or Periodic acid-Schiff (PAS) reagent. The sections were covered with coverslips immediately after a few drops of Neo-Mount (Merck) were deposited on them. For transmission electron microscopy analysis, we used 5% glutaraldehyde in 0.2 M cacodylate buffer to fix the tissues at 4 °C overnight. The next day, tissues were washed with 0.2 M cacodylate buffer, dehydrated in increasing concentration of ethanol, embedded and polymerized by the automated microwave tissue processor (Leica EMAMW). After that, samples were cut into small slices with the LEICA Ultracut UCT ultramicrotome (Leica Microsystems). Ultrathin sections were stained with uranyl acetate and lead citrate and examined by TEM (JEOL, JEM-1010).

### Immunoprecipitation

For tissue, 80-100 mg testes were put in a clean grinder dounce, lysed in lysis buffer (20 mM Tris- HCl pH 7.4, 150 mM NaCl, 0.5% Triton X-100, 0.5% sodium deoxycholate, 1 mM DTT, protease inhibitor) by grinding 30 times, and left on ice for 10 min. The lysates were diluted with dilution buffer (20 mM Tris-HCl pH 7.4, 150 mM NaCl, 0.5% Triton X-100, 1 mM DTT, protease inhibitor] and rotated at 4 °C for 1 h. The lysates were then centrifuged at 14,000 × *g*, 4 °C for 10 min. The supernatants were pre-cleaned by incubation with Dynabeads Protein A (Invitrogen™) at 4 °C for 1 h, and incubated with antibody at 4 °C overnight. Beads were washed and added to the lysates containing the antibody, and rotated at 4 °C for 3–4 h. The beads were then washed five times with wash buffer (20 mM Tris-HCl pH 7.4, 150 mM NaCl, 0.5% Triton X-100, 1 mM EDTA], resuspended in 2x loading buffer, and boiled for 10 min. Immunoprecipitated proteins were analyzed by Western blot. Five percent of the lysates was collected before the antibody addition and used as an input sample. For cell line, HEK293T cells were grown on 10 cm dish, transfected at a confluence of approximately 60% using the calcium phosphate method, harvested 48 h after transfection and washed with ice-cold PBS. Immunoprecipitation for cell line was performed as the same for tissue.

### Immunofluorescence

To prepare frozen sections for immunofluorescence analysis, testes were fixed in 10 ml 4% paraformaldehyde (PFA) at 4 °C overnight. The next day, tissues were dehydrated with 15% sucrose in 1x PBS until totally sunk to the bottom of the tube, transferred to 30% sucrose for further dehydration at 4 °C overnight. And then, embedded in OCT (Fisher Scientific, 14-373-65) and cut into 5 µm in cryostat (Thermo Scientific Cryotome FSE). Slices were stored at −80 °C. For immunofluorescence analysis, the slices were treated as follows: air dried at room temperature, washed twice with PBS, permeabilized with 0.5% Triton X-100 in PBS at room temperature for 10 min. After washing three times with PBS, samples were blocked with 5% serum in TBS-T (TBS, 20 mM Tris, 150 mM NaCl, pH 7.6, and 0.1% Tween20) at room temperature for 1 h, incubated with primary antibodies in suitable concentration at 4 °C overnight, and then conjugated with secondary antibody and DAPI at 37 °C for 1 h. Chromosome spreads of prophase I spermatocytes were conducted as described previously^45^. All fluorescence images were taken and analyzed by Carl Zeiss LSM800 confocal microscope or Image-Pro Plus 6.0. All statistical results were performed with Graph Pad Prism 6.

### Isolation of spermatogenic cells

Four types of mouse male germ cells were isolated using a BSA gradient method as previously described^68^. Spermatogonia (SG) was collected from P6-8 testes；pachytene spermatocyte (PS), round spermatid (RS), and elongating spermatid (ES) were isolated from P60 testes. After euthanasia, mouse testes were harvested, quickly washed twice in cold PBS, and then digested in 10 ml DMEM (Gibco, 12800017) containing collagenase (1 mg/ml, Gibco, 17100-017) for at 37 °C 15 min with gentle shaking. The dispersed seminiferous tubules were collected by spinning at 300 × *g* at 4 °C for 5 min, and washed twice with DMEM. Tubules were then digested in 45 ml 0.25% Trypsin (Gibco, 25200-072) containing DNase I (1 mg/ml, Qiagen) at 37 °C for 5 min with gentle shaking, filtered through 40 µm Nylon Cell Strainer (BD Falcon, 352340) and resuspended with 25 ml DMEM containing 0.5% BSA. After loading the single-cell suspension into the separation apparatus (ProScience, Canada), germ cell populations were separated by sedimentation at unit gravity for 3 h through a gradient of 2–4% bovine serum albumin (BSA) solution. After that, cell fractions were harvested, and identified by their morphological characteristics. The purity of isolated SG, PS, RS, and ES was approximately 85%, 90%, 90%, and 85%, respectively. Cell purity was analyzed by Zeiss Axio Scope A1.

### Isolation of nuclear and cytoplasmic fractions

Subcellular fractions were extracted as described previously^69^, with minor modifications. Briefly, about 100 mg testes tissues were homogenized in 1 ml Cytoplasmic Extraction Buffer (250 mM sucrose, 10 mM Tris-HCl, pH 8.0, 10 mM MgCl_2_, 1 mM EGTA, 1× protease inhibitor cocktail III) by pestling about 100 times. Nuclei were pelleted by spun down at 300 × *g* at 4 °C for 5 min, and the supernatant was collected as the cytoplasmic fraction. The cytoplasmic fraction needs another high-speed centrifugation at 14000 ×g at 4 °C for 10 min to acquire purified cytoplasmic protein. The nuclear pellet was washed three times in Cytoplasmic Extraction Buffer plus once with PBS, dissolved in Nuclear Extraction Buffer (250 mM sucrose, 10 mM Tris-HCl pH 8.0, 10 mM MgCl_2_, 1 mM EGTA, 0.1% Triton X-100, 0.25% NP-40, and 1× protease inhibitor cocktail III), and then centrifuged at 14000 × *g* for 15 min to collect supernatant as the nuclear protein.

### ASO-based knockdown in testis

We performed the transduction as previously described^62^. Briefly, P24 mice were anesthetized by tri-bromoethanol. Testes on two sides were exteriorized in order through incisions on abdomen. Under a stereoscopic microscope, one testis was injected with 4 µl *Elfn2*-targeting ASO dilutions and the other was injected with 4 µl none-targeting ASO dilutions as control. After recovery, mice were fed as before. Eleven days later, all testes were harvested for the subsequent experiments. ASO oligonucleotide sequences are listed in Supplementary Data 1.

### Plasmid construction and dual luciferase reporter assay

The *Elfn2* (ENSMUST00000088592.6) 5′ UTR region was cloned into psiCHECK™-2 luciferase vector. The wild-type and two mutant *Ddx43* CDS regions (*Ddx43*-WT*, Ddx43*-KO*, Ddx43*-KI) were separately cloned into pRK5-FLAG vector. These plasmids are termed as psi-*Elfn2*−5′ UTR, FLAG-DDX43-WT, FLAG-DDX43-KO and FLAG-DDX43-KI, respectively. The primer sequences for cloning are listed in Supplementary Data 1. We co-expressed psi-*Elfn2*−5′ UTR and each FLAG-DDX43 in HEK293T cells. After transfection for 48 h, the cells were lysed and measured with Luciferase Assay Kit (Promega, E2920). The results were analyzed by BioTek Synergy 2 enzyme-labeled. Firefly luciferase values were normalized to Renilla and measurements of mean ± SD of relative luciferase unit per microgram of protein were taken in triplicates and represented graphically as mean ± SD on a MS-Excel sheet.

### Protein expression and purification

The wild-type and knock-in *Ddx43* CDS regions (*Ddx43* or *Ddx43*-KI) were cloned into pET28a expression vector. The primer sequences for cloning are listed in Supplementary Data 1. These plasmids were transformed into *E. coli* BL21 cells. Expression of DDX43 or DDX43-KI protein was induced with 0.3 mM isopropyl-β-d-thiogalactopyranoside (IPTG) at 15 °C overnight. Cells were harvested at 6000 ×g at 4 °C for 10 min, and lysed by sonication (output power 10%, 30 cycles of 10 s on, 15 s off) in lysis buffer (50 mM Tris-HCl pH 7.0, 0.5 M NaCl, 2 mM MgCl_2_, 10% glycerol, 0.1% Triton X-100, 1 mg/ml lysozyme, 1 mM phenylmethylsulfonyl fluoride (PMSF), 10 mM imidazole, 1× protease inhibitor cocktail III) at 4 °C. The lysates were spun at 5000 g for 30 min, transferred the supernatant to another new tube and incubated with Ni–nitrilotriacetic acid (NTA) agarose beads (Qiagen, 30210) in binding buffer (50 mM Tris-HCl pH 7.0, 0.5 M NaCl, 2 mM MgCl_2_, 10% glycerol, 0.1% Triton X-100, 10 mM imidazole) at 4 °C for 3 h. After washing the Ni-NTA bead–protein complexes with wash buffer (25 mM Tris-HCl pH 8.0, 0.5 M NaCl, 100 μM Tween 20, and 10% glycerol) containing 25 mM imidazole, proteins were eluted with wash buffer containing 250 mM imidazole, evaluated for purity on SDS-PAGE and stored at −80 °C till used for EMSA.

### EMSA

Biotin-labeled RNAs were synthesized from Tsingke Biotech (Beijing, China). The RNA sequences are listed in Supplementary Data 1. Each oligo was diluted to 10 µM in RNase free water and kept at −80 °C for further use. RNA G4 substrates were prepared by heating an ssRNA oligo in the G4 formation buffer (50 mM Tris-HCl pH 7.5, 150 mM KCl, 0.1 mM EDTA) at 95 °C for 5 min followed by slow cooling to room temperature. To form RNA/protein complexes, we incubated the indicated concentrations of purified DDX43 or DDX43-KI proteins with 10 nM of a Biotin-labeled RNA substrate in binding buffer (50 mM Tris-HCl pH 7.5, 100 mM KCl, 2.5% glycerol, 0.05% NP-40, 4 mM EDTA, 2 U of RiboLock RNase Inhibitor, 1 mM DTT) at 37 °C for 30 min. After the incubation, 5X stop buffer (125 mM EDTA, 50% glycerol) was added to each mixture, and samples were resolved on 8% native TBE PAGE gel at 100 V for 1 h at 4 °C, electrically transferred from gel to nylon membrane at 90 mA for 50 min, and visualized by an enhanced chemiluminescence detection system (Tanon-5200).

### Radiolabel detection of piRNA and CLIP-captured RNA

Mouse testicular piRNA was radiolabeled as described previously^50^. Briefly, total RNA (1 µg) extracted from adult mouse testes was treated with alkaline phosphatase (NEB), de-phosphorylated, and 5′ end-labeled using T4 polynucleotide kinase (NEB) and [γ-^32^P] ATP. Then, we ran Urea-PAGE gel to separate the ^32^P labeled RNA. Finally, by exposing the gel to a phosphorimager screen and scanning with a Typhoon scanner (GE Healthcare), radioactive signals were visualized.

We performed DDX43 conventional CLIP as previously for MOV10^69^. Briefly, 100 mg testis tissues were detunicated with 4 ml cold PBS, UV- irradiated on a 10 cm plate at 254 nm three times inside UV cross-linker (UVP CL-10000), centrifuged at 1000 ×g, 4 °C for 1 min to collect the pellet, flash-frozen in liquid nitrogen, and then stored pellet at −80 °C or immediately used. To continue CLIP, pellets were dissolved with 300 µl 1X PMPG buffer (1x PBS no Mg^2+^/Ca^2+^, 2% Empigen) containing protease inhibitors (Roche, 11873580001) and RNasin (Promega), treated with DNase (Promega, M6101) at 37 °C for 10 min in a Thermomixer, and then spun down at 15,000 rpm at 4 °C for 30 min. The supernatant was then immunoprecipitated with anti-DDX43 antibodies using protein A Dynabeads (Invitrogen, 10002D). Beads were washed with 1x and 5x PMPG and treated with alkaline phosphatase (NEB) to de-phosphorylate DDX43-crosslinked RNA on beads. And then, the [γ-^32^P] ATP-labeled RNA linker (RL3) was added to beads for RNA ligation overnight. The next day, after stringent wash steps, crosslinked DDX43 RNPs were boiled with NuPAGE™ LDS (Life Technology, NP0007), separated by NuPAGE™ 10% Bis-Tris (Life Technology, NP0323BOX), and transferred onto nitrocellulose (Invitrogen, LC2001). Membranes were exposed to phosphorimager screen for autoradiography.

### eCLIP-seq libraries

We prepared eCLIP libraries (with biological duplicates) as previously described^57,58^. Briefly, testicular seminiferous tubules were harvested and UV-irradiated as described for conventional CLIP, lysed and sonicated with 1 ml fresh eCLIP lysis (50 mM Tris-HCl pH 7.4, 100 mM NaCl, 1% NP-40 (Igepal, CA630), 0.1% SDS, 0.5% sodium deoxycholate (protect from light), 1:200 Protease Inhibitor Cocktail III), and then, incubated with RNase I (Life Technology, AM2295) and DNase (Promega, M6101) in a Thermomixer at 1200 rpm to fragment RNA at 37 °C for 10 min. Then, DDX43-RNA complexes were immunoprecipitated with anti-DDX43 antibodies, followed by dephosphorylation of RNA fragments and ligation of 3′ RNA adapter. After stringent wash with low- and high-salt buffers, eCLIP samples, as well as SMInput samples (as control library to subtract noise from eCLIP-seq library), were boiled from beads, run on an SDS-PAGE gel and transferred to nitrocellulose membranes. In parallel, a small proportion of samples was reserved for Western blot analysis, which validates specific enrichment of DDX43 protein and identifies cutting territory on membrane. And then, RNA from both eCLIP and SMInput were harvested from the membrane by digesting the protein with proteinase K and Urea. The harvested RNA was reverse transcribed with Affinity Script (Agilent,600107), purified by Exo-Sap-IT (Affymetrix, 78201) to remove unincorporated primer, and further ligated with 3′ DNA adaptor. Finally, we used Q5 PCR mix (NEB, M0492L) to amplify the libraries for high-throughput sequencing in Illumina platform.

### RNA-seq libraries

We used TruSeq Stranded Total RNA kit (Illumina) for library preparation. Sequencing of 9 RNA-seq libraries (with triplicate samples for each genotype) was performed. Libraries were assessed with the Qubit and Tapestation for molarity and quality before submitting to the Illumina Hiseq X ten system.

### scRNA-seq libraries

We performed testis scRNA-seq from adult *Ddx43*^+/+^, *Ddx43*^–/–^ and *Ddx43*^KI/KI^ mice (with biological duplicates) as previously described^26^. Briefly, testes were digested into single cell suspensions as the performance in isolation of spermatogenic cells. Cell concentration was counted by Cellometer Mimi (Nexcelom Bioscience). Mixed the cell suspension with Trypan Blue (Life Technology), and then counted the viability of cells by the introverted microscope. Only samples with >80% viability were loaded into Single Cell A Chip (10X Genomics, Chromium) and recovered about 5000 cells. Chromium Single Cell Controller (10X Genomics) was used to acquire single cell Gel Bead-In-EMusions (GEMs). Finally, libraries were constructed with Single Cell 3′ Reagent Kits v2 before sequencing by Illumina Novaseq.

### Bioinformatics analyses

#### Processing of RNA-seq data

The reads were mapped to the GENCODE GRCm38 v23 transcript set using Bowtie2 and the gene expression level was estimated using RSEM.TMM (trimmed mean of *M* values) was used to normalize the gene expression. Differentially expressed genes were identified using the edgeR program. Genes showing altered expression with FDR adjusted *P* value <0.05 and absolute log 2 (fold change) > 1 were considered differentially expressed.

#### Processing of eCLIP-seq data

Reads were adaptor trimmed through cutadapt, and mapped to mouse genome (with repetitive elements-mapping reads discarded) with STAR. PCR duplicate reads were removed. We then used CLIPper to perform peak-calling. Input normalization of peaks was performed by counting reads mapping to CLIPper-identified peaks in eCLIP and paired SMInput dataset, with significance thresholds of FDR adjusted *P* value ≤ 10^−5^ and fold enrichment ≥ 4. The binding affinity of each peak is defined by counting reads mapping to peaks and perform TPM normalization.

#### eCLIP-seq motif identification and G4 prediction

For the peaks identified in each replicate, we ranked the peaks according to the significance. The top 50% most significant (ranking according to FDR adjusted *P* value) peaks were used to perform MEME analysis (The MEME Suite). We set the maximum length of identified motif equal to 12. We used G4Hunter (Re-evaluation of G4 propensity with G4Hunter) to evaluate the G4 score of each peak and its flanking sequence with the same length.

### Processing of 10X genomics scRNA-seq data

#### Read filtering and alignment

Raw sequencing data were converted to fastq format using cellranger mkfastq (10x Genomics, v3.0.0). scRNA-seq reads were alighed to the GRCm38 (mm10) reference genome and quantified using cellranger count (10x Genomics, v.3.0.0). After collecting digital gene expression count matrix, we performed quality control to the cells based on the distribution of genes detected and UMIs, mitochondrial transcripts percent of each cell for all experiments. On the basis of these distribution, we filtered out the cells with detected genes ≤ 2.5 quantile or ≥ 97.5 quantile regarded as the outlier cells for each experiment, and we also filtered out cells with ≥ 10% of transcripts corresponding to mitochondria-encoded genes. Furthermore, we computed pearson residuals of our expression dataset through *SCTransforms* functions in Seurat, with regarding cell cycling score, mitochondria-encoded genes transcripts level, and the total number of UMIs for each cell as the covariates.

#### Module activity matrix construction and cell type annotation

We used previously developed algorithms ICAnet^26^ to convert normalized gene expression matrix to the module activity matrix. ICAnet used both independent component and protein-protein interaction network to identify gene co-expression modules, using those modules could facilitate cell clustering and batch integrations. For WT samples, we used JADE algorithms to compute independent components combine with STRING^70^ network to identify modules through random walk trapping. Using identified module lists, we could quantify those modules activity on both WT and KO samples through AUCell^51^. We finally identified 1085 modules in our data, and used AUCell induced module activity matrix to perform downstream analyses. To define high-quality reference atlas, we first analysis WT samples, and used their module activity matrix to infer top 20 principal components to calculate Euclidean distance among all pairs of cells and for each, identified its 30 nearest neighbors to define cell *k*-nearest neighborhood (KNN) graph. It then used the Louvain method of clustering using Jaccard distance among cells as weights, where the Jaccard distance between ant two cells was defined as the degree of overlap of their 30 nearest neighbors, then using UMAP^71^ to perform visualization. We then annotate each cluster according to known spermatogenesis marker genes expression pattern within each cluster.

#### Mutant to reference mapping and label transferring

After defining the reference map. We then using anchors base integration methods provided in Seurat. Using defined new assay, module activity matrix, we projected cells from mutant samples on reference samples through looking the mutual nearest neighbor cell in reference samples for each cell in mutant sample, and defined as anchor, we then transferred the labels (cell type annotations) in reference samples to the cells in mutant samples through those anchors, and only preserve those cells which prediction score ≥ 0.95 to get a high confidence prediction.

#### Cell differentiation potency quantification

To measure the cell differentiation potency, we used a signaling entropy model names single cell entropy (SCENT)^52^ to estimate each cell’s plasticity. SCENT could quantify the degree of uncertainty of a cell’s gene expression levels in the context of a cellular interaction network through constructing a stochastic matrix base on PPI network for each cell. However, the original signaling entropy score need to infer an invariant measure Π for each cell, which rely on singular value decomposition and it need to cost tremendous time for computation. Therefore, we used a global mean field approximation of the signaling entropy, that is, the Pearson correlation of the cell’s transcriptome and the connectome from the PPI network.

#### Single cell clustering evaluation

To optimize STids (spermatids) cell clustering. We used two metrics to evaluating different clustering methods and parameter setting.

##### Inverse normalized condition number (INCN) score

To measure the transcriptomic distinctness of each cluster. Suppose we have a clustering label, we aggregated the cells according to the label to get the average gene expression value of each cluster. Then we calculated the matrix 2-norm condition number, which is defined as the ratio of the largest to the smallest non-zero singular value of the matrix, and could evaluate the collinearity among different cell states expression. Invert the value of matrix condition number to get Inverse Condition Number (ICN) Score, we expected the resulted score should be large in optimal clustering results, as the collinearity of the expression among different cell state is small. To normalize this score, we shuffled the initial cluster label, and repeat to calculate the ICN score under randomization, calculated the average value and use this value to normalize the raw ICN score, and finally we could get the INCN score.

##### Explained variance (EV) score

A clustering results should recover the most of biological variation. To evaluate this aspect, we used FACS annotation results (select spermatids phase cell states) of our reference single cell gene expression dataset to aggregate reference cell expression matrix into average gene expression matrix of annotated cell types. We also aggregated our cell gene expression matrix into cell state gene expression matrix according to clustering result, and perform regression analysis of each annotated cell state with our aggregate gene expression dataset. And calculated the average explained variance. We expected that an optimal clustering result should explain more variance.

#### Single cell gene expression imputation

Previous researches have shown that using imputed gene expression could identify latent cell state structure. To show the latent cell states in STids cells. We smoothed gene expression through Laplacian regularization. This method is a simple technique that targets signal smoothness via the optimization

$${{{{{\boldsymbol{y}}}}}}=argmi{n}_{{{{{{\boldsymbol{z}}}}}}}{|{{{{{\boldsymbol{x}}}}}}-{{{{{\boldsymbol{z}}}}}}|}_{2}^{2}+\beta {{{{{{\boldsymbol{z}}}}}}}^{T} {\mathcal L} {{{{{\boldsymbol{z}}}}}}$$

Note that this optimization has two terms. The first term called a reconstruction penalty, aims to keep the density estimate (${{{{{\boldsymbol{z}}}}}}$) similar to the input sample information, which is, the gene expression (${{{{{\boldsymbol{x}}}}}}$). The second term ensures smoothness of the imputed signal. Balancing these terms adjusts the amount of smoothness performed by the filter. The Laplacian matrix (${{{{{\mathscr{L}}}}}}$) defined as follows:

$${{{{{\mathscr{L}}}}}}{{{{{\mathscr{=}}}}}}D-W$$

where *D* is the degree matrix, which is diagonal matrix with ${D}_{{ii}}=d\left(i\right)={\sum }_{j}{W}_{{ij}}$ containing the degrees of the vertices of the graph defined by W. In our analysis, we used Shared Nearest Neighborhood graph to define Laplacian matrix. We performed Laplacian regularization to each gene with parameter $\beta=10$. We noted that we only used the imputed gene expression for visualization but not for any other analysis.

#### RNA velocity and trajectory analysis

To infer future states of individual cells, we used spliced and unspliced transcript ratio to predict cell differentiation trajectory. This combinational analysis was performed using a combination of the Seurat and scVelo^27^ code. First, we used cellranger output aligned bam files as input for Velocyto to derive the counts of unspliced and spliced reads in loom format. Next, the non-spermatogenesis cells were excluded from the datasets, and we saved previously annotated cell type label, computed principal components and UMAP embedding (generated from “*integrate*” assay) and the spliced and unspliced read count matrix as new Seurat object, and converted the cleaned Seurat object into a.h5ad file for downstream analysis using python package scVelo. Each sample (*Ddx43*^+/+^, *Ddx43*^KI/KI^ and *Ddx43*^–/–^) loom files were normalized and log transformed using scVelo functions normalize_per_cell() and log1p(), then we used to calculate first and second-order moments for each cell across its nearest neighbors (scvelo.pp.moments(n_pcs = 20, n_neighbors = 30)). Next, the velocities were estimated and the velocity graph constructed using the scvelo.tl.velocity() with the mode set to ‘stochastic’ and scvelo.tl.velocity_graph() functions. Velocities were visualized on top of the previously calculated UMAP coordinates with the scvelo.tl.velocity_embedding() function. To compute the terminal state likelihood, the function scvelo.tl.terminal_states() with default parameters was used.

#### Evaluating the confidence of DEG identification results

For each genotype, *Ddx43*^KI/KI^ and *Ddx43*^–/–^, we have two biological replicates. Normally we performed DEG analysis through merging the cells from both samples, and using Wilcoxon rank-sum test to perform differential expression analysis for each gene. However, we need to account for the individual differences or batch effects may lead different DEG results. To assess the effects, we performed DEG analysis for each biological replicate to wild-type samples, and calculated the correlation of two gene expression FoldChange vector, which is derived from two biological replicate.

#### Reclustering of STids and building PAGA graph

To identify the latent cell state within spermatids (STids). We extracted the cells from STids of integrated samples (includes all *Ddx43* genotypic samples), and re-computed the principal components (PCs). We used top 10 PCs to build cell KNN graph, and using Louvain clustering algorithm with resolution parameter set as 0.25 to identify cell sub-clusters. Using this pipeline, we found four sub-clusters within STids, denoted as sub-cluster 0-4. To assess the global connectivity topology between the Louvain clusters we appliced Partition-based graph abstraction (PAGA)^56^. We applied the tl.paga() function integrated in the Scanpy package to calculate connectivities and used the predefined Louvain clusters as partitions. The weighted edges represent a statistical measure of connectivity between the partitions. The orientation of the edges between each Sub-cluster is given by previous RNA velocity analysis.

##### Identification of DEGs

To identify the DEGs between *Ddx43*^KI/KI^ and *Ddx43*^+/+^ samples within each sub-cluster, we only extracted the normalized spliced RNA expression matrix and used Wilcoxon rank-sum test to perform DEG analysis. We further used Benjamini & Hochberg method to perform multiple-test correction. Meanwhile, we also calculated each gene expression difference between *Ddx43*^KI/KI^ and *Ddx43*^+/+^ samples using following equation.

$${{ExpressinDifference}}_{i}=\frac{1}{{n}_{{ko}}}\mathop{\sum }\limits_{j=1}^{{n}_{{ko}}}{X}_{{ij}}-\frac{1}{{n}_{{wt}}}\mathop{\sum }\limits_{j=1}^{{n}_{{wt}}}{X}_{{ij}}$$

##### Weighted Gene Co-expression Network Analysis (WGCNA)

To prevent the influence of batch effects between *Ddx43*^KI/KI^ samples and *Ddx43*^+/+^ samples. We only extract the cells of STids from *Ddx43*^+/+^ samples and used the downregulated genes in sub-cluster 0 and sub-cluster 1 to perform WGCNA^59^ analysis. Unsigned pearson correlation was first used to calculate pairwise correlations between genes. Next, pairwise topological overlap was calculated with a power of 2 based on a fit to scale-free topology. Co-expression modules comprised of correlated genes with high topological overlap were then identified using the cutreeDynamic function in the dynamicTreeCut R package, with the following parameters: deepSplit = 2, pamRespectsDendro = TRUE, minClusterSize = 50. We applied AUCell to quantify the activity of each module in each cell from *Ddx43*^*+/+*^ and *Ddx43* mutant samples. To visualize the gene expression pattern of modules, we extract the top 20 gene with the highest gene connectivity computed through *intramodularConnectivity* function, and visualize their expression pattern through a heatmap with the cells are ordered according to the velocity time.

#### Driver Gene Identification Using Random Walk with Restart algorithm

Guilt-by-association analysis have been widely used to identify possible driver candidates relate to the phenotype of interests. Specifically, we hypothesize that the genes proximal to the four co-expression modules probably could be regarded as the key genes. Therefore, we need to define a topological distance metric to assess the distance between all genes and the four modules respectively, and aggregate distance information to derive a ranking list which represents the global distance of each genes to the four modules. We used Random walk with restart (RWR)^72^, a network diffusion algorithm to measure the distance. Different from conventional random walk methods, RWR introduces a pre-defined restart probability at the initial genes for every iteration, which can take into consideration both local and global topological connectivity pattern within the network to fully exploit the underlying direct or indirect relations between nodes. First, we converted the gene topological overlapped matrix (TOM) derived from WGCNA analysis into gene level transition matrix as follow.

$${P}_{{ij}}=\frac{{{TOM}}_{{ij}}}{{\sum }_{j}{{TOM}}_{{ij}}}$$

Formally, TOM denote the topological overlapped matrix which represents the adjacency matrix of a gene co-expression network with m genes. The matrix P describes the probability of a transition from gene i to gene j. Next, let ${s}_{k}^{t}$ be an m-dimensional distribution vector in which each element stores the probability of each node being visited after t iterations in the random walk process, with the starting points set from the *k*th module genes. Then RWR from node *i* can be defined as

$${s}_{k}^{t+1}=\left(1-{p}_{r}\right){s}_{k}^{t}P+{p}_{r}{e}_{k}$$

Where ${e}_{k}$ stands for an m-dimensional standard basis vector with ${\left({e}_{k}\right)}_{i}\left(i\in {{module}}_{k}\right)=1$, indicate whether the genes belong to the specific module (e.g., brown module). And ${p}_{r}$ stands for the pre-defined restart probability, which actually controls the relative influence between module local and global topological information in the diffusion process. We used following metric to stop the iteration process.

$${{{{{\rm{cor}}}}}}\left({s}_{k}^{t+1},{s}_{k}^{t}\right) \, > \, 0.999$$

That means once the ${s}_{k}^{t}$ reach a stationary distribution, we stop the iterations. We referred the stationary distribution as the “steady states”, and each element of the steady states vector can be regarded as the inverse of topological distance to the modules. We then aggregated steady states probability vector into the joint association vector defined as follow:

$${{{{{\rm{a}}}}}}={\prod }_{k}{s}_{k}$$

Using the association vector $a$, we could rank all genes and selected the top 30 genes regarded as the proximal genes to all four modules.

### Reporting summary

Further information on research design is available in the Nature Portfolio Reporting Summary linked to this article.

## Supplementary information

Supplementary Information

Description of Additional Supplementary Files

Supplementary Data 1

Reporting Summary

## Data Availability

The raw scRNA-seq datasets of mouse whole testis (fastq format) have been deposited to NCBI-SRA with the accession number PRJNA650016. The raw eCLIP-seq and RNA-seq datasets of mouse whole testis (fastq format) have been deposited to NCBI-SRA with the accession number PRJNA838233. Source data are provided with this paper.

## Source data

Source Data

## Code availability

The code to reproduce the main results in this manuscript is available from the Github repository: https://github.com/WWXkenmo/MouseGerm_DDX43. Processed datasets are available at Zenodo with the access code 10.5281/zenodo.7479750.
