# Supplementary material for: Single-cell RNA-seq uncovers dynamic processes orchestrated by RNA-binding protein DDX43 in chromatin remodeling during spermiogenesis
Source: Nat Commun. 2023 Apr 29;14:2499. doi: 10.1038/s41467-023-38199-w (PMC10294715; doi:10.1038/s41467-023-38199-w)
Supplement: Supplementary file 1 — Supplementary Information [file 41467_2023_38199_MOESM1_ESM.pdf]

Supplementary Figures and Legends S1 to S19

Single-Cell RNA-Seq Uncovers Dynamic Processes Orchestrated by RNA-Binding Protein DDX43 in Chromatin Remodeling during Spermiogenesis

Huanhuan Tan[1,^,†], Weixu Wang[2,#,†], Congjin Zhou[1,†], Yanfeng Wang[1,†], Shu Zhang[1], Pinglan Yang[1], Rui Guo[1], Wei Chen[2], Jinwen Zhang[1], Lan Ye[1], Yiqiang Cui[1,*], Ting Ni[2,*], Ke Zheng[1,*]

[1]State Key Laboratory of Reproductive Medicine, Nanjing Medical University, Nanjing 211166, China.

[2]State Key Laboratory of Genetic Engineering, Collaborative Innovation Center of Genetics and Development, Human Phenome Institute, Shanghai Engineering Research Center of Industrial Microorganisms, School of Life Sciences and Huashan Hospital, Fudan University, Shanghai 200438, China.

[^]Present affiliation: Reproductive Medicine Center, The First Affiliated Hospital of Chongqing Medical University, No. 1 Youyi Road, Yuzhong District, Chongqing 400016, China.

[#]Present affiliation: Institute of Computational Biology, Helmholtz Center Munich, Munich, Germany.

† These authors contributed equally to this work

*Correspondence: cuiyiqiang@126.com, tingni@fudan.edu.cn, kezheng@njmu.edu.cn

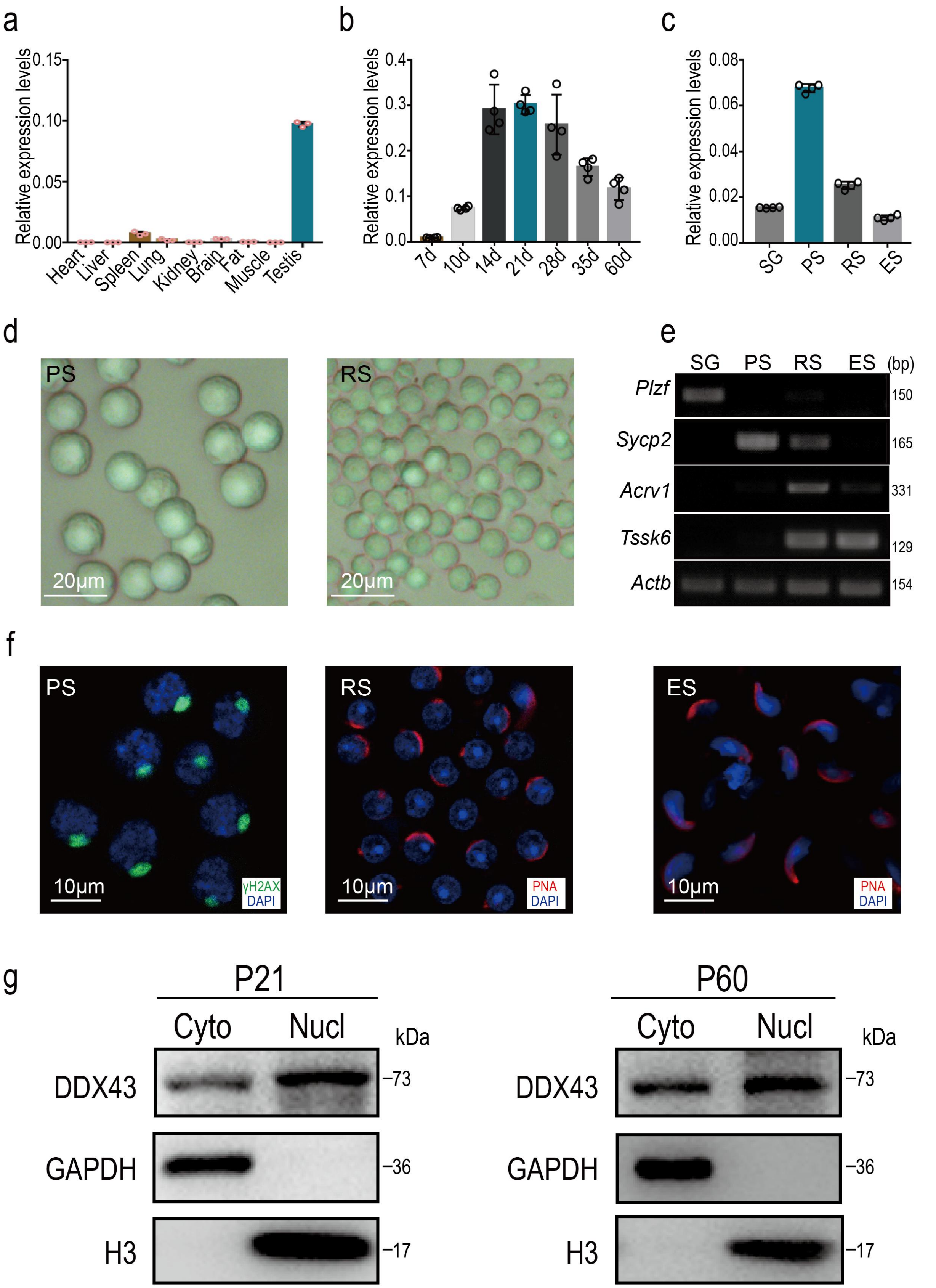

Supplementary Figure 1. ***Ddx43*** **expression and purified germline populations.**

Related to Figure 1 and 7.

(a-c) Quantitative RT-PCR (qRT-PCR) analyses of *Ddx43* mRNA transcripts from multiple adult mouse tissues (a), testis tissues collected from mice at different ages indicated (b), and various types of isolated spermatogenic cell populations (c). SG, spermatogonia (approximately 85% purity); PS, pachytene spermatocytes (approximately 90% purity); RS, round spermatids (approximately 90% purity); ES, elongating spermatids (approximately 85% purity). Results are normalized to *Rplp0* (*36b4*). Data presented are mean ± SD from three independent experiments. **(d)** Morphology analyses of purified pachytene spermatocytes (PS) and round spermatids (RS). Scale bar is indicated. **(e)** RT-PCR analyses of key marker genes further confirm the identity of cell populations, including *Plzf* for spermatogonia, *Sycp2* for meiotic spermatocytes, *Acrv1* for round spermatids, *Tssk6* for post-meiotic spermatids. *Actb* serves as a control. **(f)** Immunofluorescence staining of specific marker protein γ-H2AX (green) and PNA (red) for isolated pachytene spermatocytes (PS), round spermatids (RS) and elongating spermatids (ES). DNA was counterstained with DAPI. Scale bar is indicated. **(g)** Protein levels of DDX43 in cytoplasmic (Cyto) and nuclear (Nucl) fractions of mouse testes at ages indicated. GAPDH and histone H3 are cytoplasmic and nuclear positive markers, respectively. Each experiment was repeated three times with similar results.

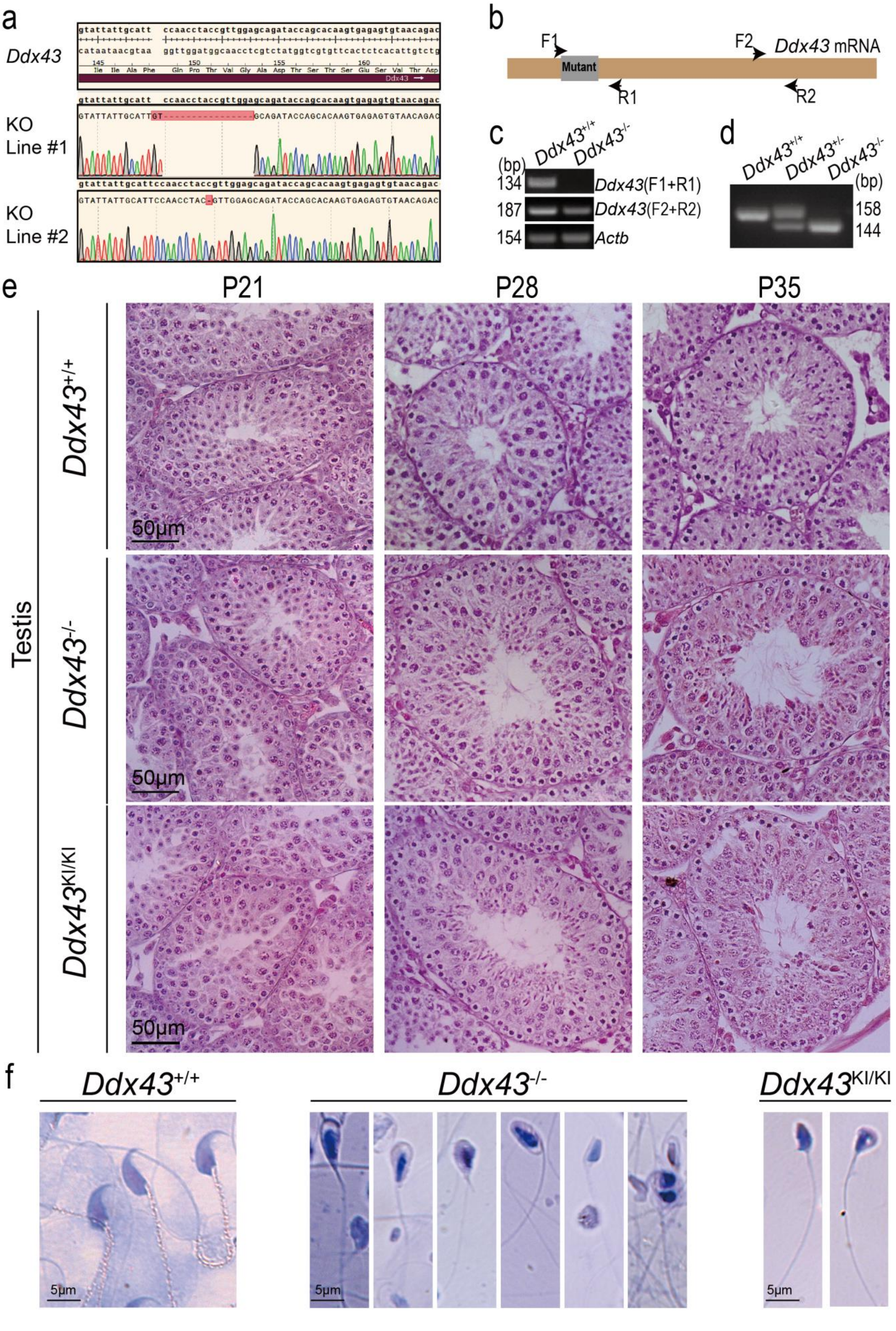

Supplementary Figure 2. Sequence validation and phenotypic analyses in *Ddx43* mutant mice. Related to Figure 1 and 2.

(a) Chromatogram of Sanger sequencing in *Ddx43*$^{+/+}$ and *Ddx43*$^{-/-}$ mice, showing the successful generation of *Ddx43* mutant. **(b)** Primer design of RT-PCR for identifying wild-type and mutant *Ddx43* transcripts. Primers F1/R1 and F2/R2 are positioned relative to sgRNA-1 and -2's targeting sites. **(c)** Based on the RT-PCR and primer pairs shown in panel (b), full-length transcripts are present in wild-type testes, but not in those of *Ddx43*$^{-/-}$ mice, which only contain truncated *Ddx43* mRNA. *Actb* serves as a control. **(d)** Genotyping PCR products of *Ddx43*$^{+/+}$, *Ddx43*$^{+/-}$ and *Ddx43*$^{-/-}$ mice, respectively. PCR products were confirmed by Sanger sequencing, shown identical in panel (a). **(e)** Hematoxylin and Eosin (H&E) staining of testes sections from *Ddx43*$^{+/+}$, *Ddx43*$^{KI/KI}$ and *Ddx43*$^{-/-}$ mice at indicated postnatal time points. Note the germ cell defects emerged in P28, when the nuclei of spermatids become flattened and start to elongate along with condensation of chromatin. Scale bar are indicated. **(h)** Acidic Aniline staining of epididymal sperm from adult mice. In contrast to the canonical hook-shaped appearance of sperm heads in *Ddx43*$^{+/+}$ mice, all sperm heads in *Ddx43*$^{-/-}$ and *Ddx43*$^{KI/KI}$ mice are amorphous with a smaller and more rounded shape. Scale bars are indicated. Each experiment was repeated three times with similar results.

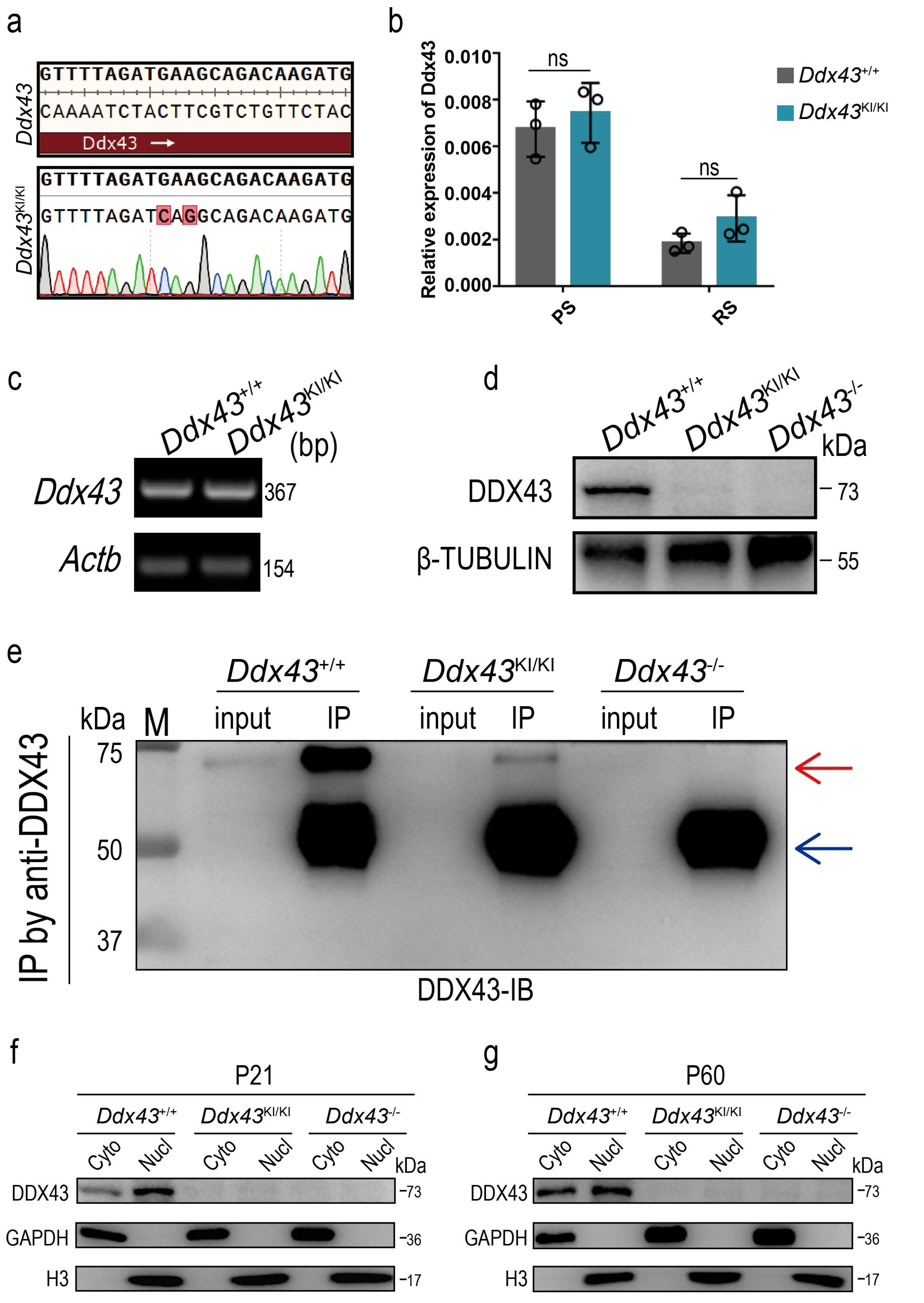

Supplementary Figure 3. DDX43 expression in *Ddx43*$^{KI/KI}$ testis. Related to Figure 2.

(a) Chromatogram of Sanger sequencing validates the successful generation of *Ddx43*$^{KI/KI}$ mutant mice. **(b)** qRT-PCR analysis of *Ddx43* mRNA transcripts in germ cells isolated from adult *Ddx43*$^{+/+}$ and *Ddx43*$^{KI/KI}$ testes. PS, pachytene spermatocytes; RS, round spermatids. Results are normalized to *Rplp0* (*36b4*) as an internal control. Data presented are mean ± SD from three independent experiments. ns, not significant; P = 0.1910 (PS), P = 0.0949 (RS); two-tailed paired Student's *t*-test. **(c)** RT-PCR gel analysis of *Ddx43* mRNA transcripts from adult *Ddx43*$^{+/+}$ and *Ddx43*$^{KI/KI}$ testes. *Actb* serves as a control. **(d)** Western blot analysis of DDX43 protein in lysates from adult *Ddx43*$^{+/+}$, *Ddx43*$^{KI/KI}$ and *Ddx43*$^{-/-}$ testes. β-TUBULIN serves as a loading control. **(e)** Western blot analyses of DDX43 protein in its immunoprecipitation (IP) lysates from testes of indicated genotypes. The *Ddx43*$^{KI/KI}$ mutant protein is detectable at a greatly reduced level after IP enrichment. Red and blue arrows point to DDX43 protein and Heavy chain. **(f, g)** Western blot analyses of DDX43 protein in cytoplasmic (Cyto) and nuclear (Nucl) fractions from P21 and P60 testes of indicated genotypes. GAPDH and histone H3 serve as markers for cytoplasm and nucleus, respectively. Each experiment was repeated three times with similar results.

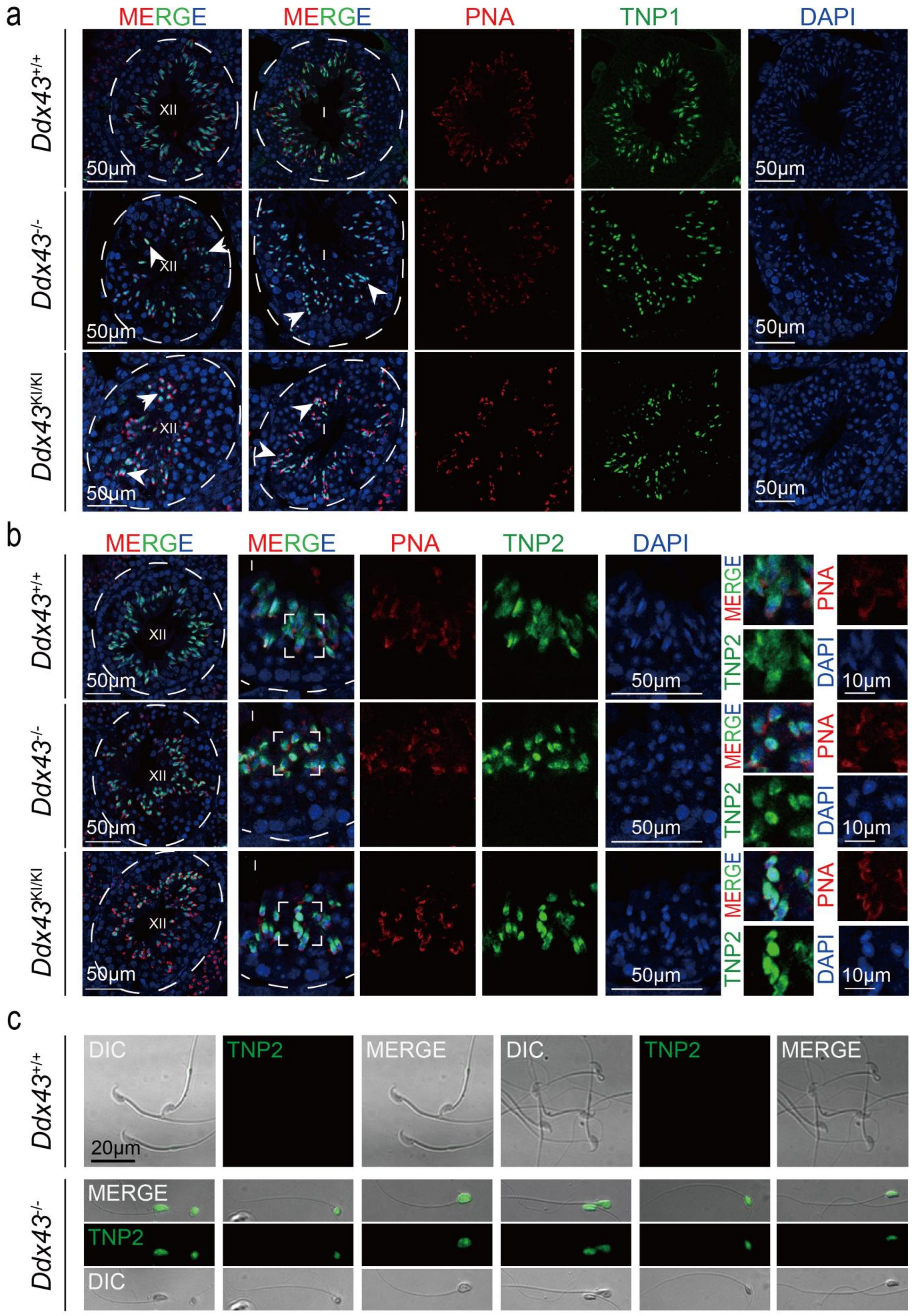

Supplementary Figure 4. Localization of TNPs in adult testis. Related to Figure 3.

(a) Co-immunofluorescence staining of TNP1 (green) and PNA (red) in testis sections from adult mice of indicated genotypes at stages XII-I, showing comparable expression abundance between *Ddx43*$^{+/+}$, *Ddx43*$^{KI/KI}$ and *Ddx43*$^{-/-}$ mice. The elongating spermatids nuclei in *Ddx43*$^{+/+}$ mice were canonical hook-shaped and thinner along with differentiation; yet, the nuclei of spermatids were deformed as rod-shaped or round-like in *Ddx43*$^{KI/KI}$ and *Ddx43*$^{-/-}$ mice. White arrows mark abnormal spermatids. DNA was counterstained with DAPI. Scale bars are indicated. **(b)** Co-immunostaining of TNP2 (green) and PNA (red) in *Ddx43*$^{KI/KI}$ and *Ddx43*$^{-/-}$ mice revealed anomalous morphological structures similar to panel (a). DNA was counterstained with DAPI. Scale bars are indicated. **(c)** Representative immunostaining images of TNP2(green) and PNA (red) in sperm from indicated genotypes. Note the abnormal head shape and TNP2 localization, indicative of less condensed mutant sperm. Scale bar is indicated for all panels. Each experiment was repeated three times with similar results.

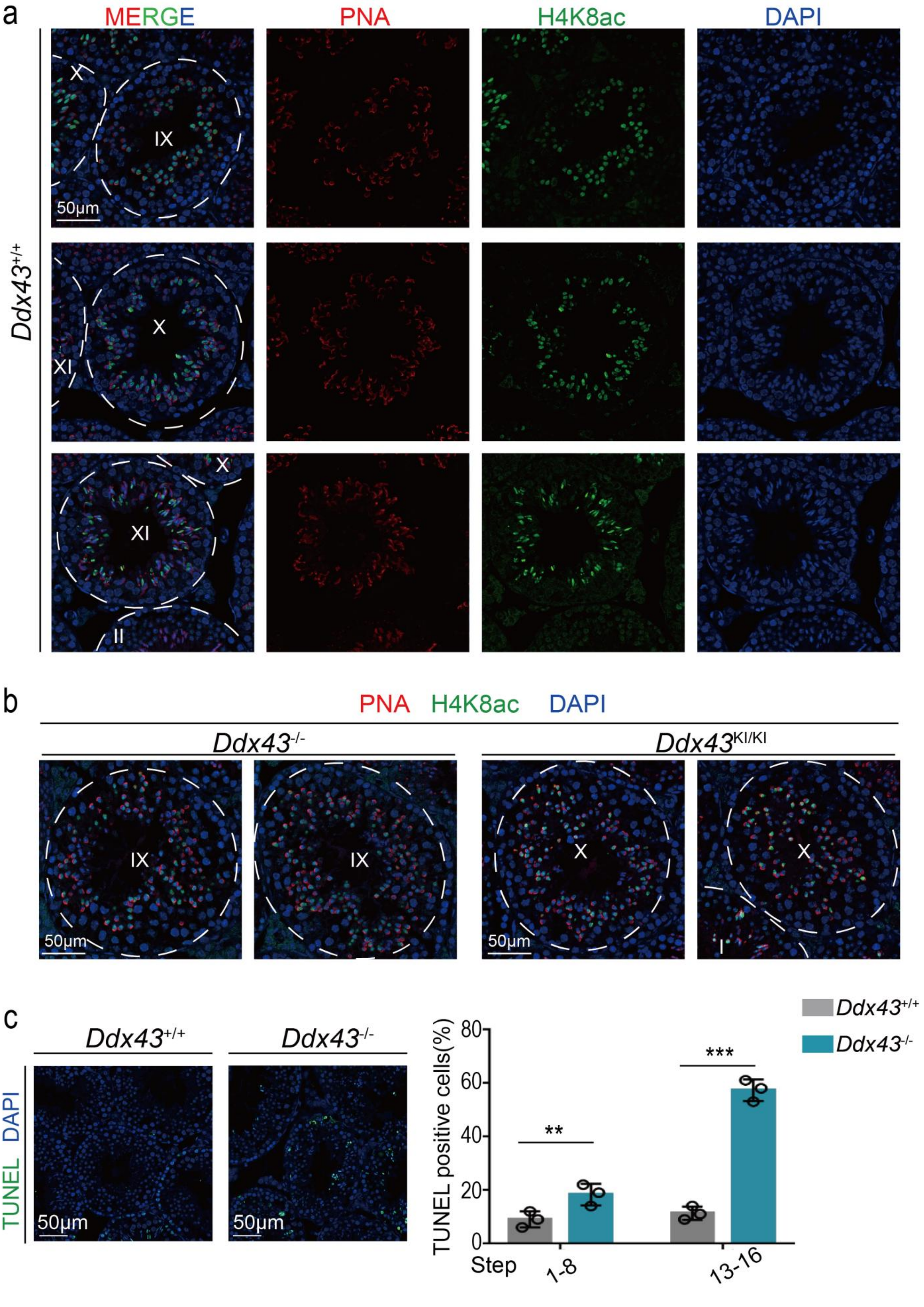

Supplementary Figure 5. Localization of H4K8ac in adult testis. Related to Figure 3.

(a, b) Immunofluorescence analyses of H4K8ac in adult *Ddx43*$^{+/+}$, *Ddx43*$^{KI/KI}$ and *Ddx43*$^{–/–}$ seminiferous tubules. In *Ddx43*$^{+/+}$ sections, H4K8ac emerges in step 9 spermatids at stage IX, peaks in step 10-11 spermatids at stage X-XI, disappears from step 12 onward. And the comparable expression intensity in mutant spermatids indicates DDX43 protein is dispensable for H4 acetylation during spermiogenesis. Stage numbers and scale bars are indicated. **(c)** Representative images (left) and statistics of positive cells (right) in TUNEL assay on adult *Ddx43*$^{+/+}$ and *Ddx43*$^{–/–}$ sections. Note the increased signals from the mutant elongating spermatids, indicative of increased cell death. Data are presented as mean ± SD from three independent experiments. *** P = 0.0005, ** P = 0.0051; two-tailed paired Student's *t*-test. Scale bar is indicated.

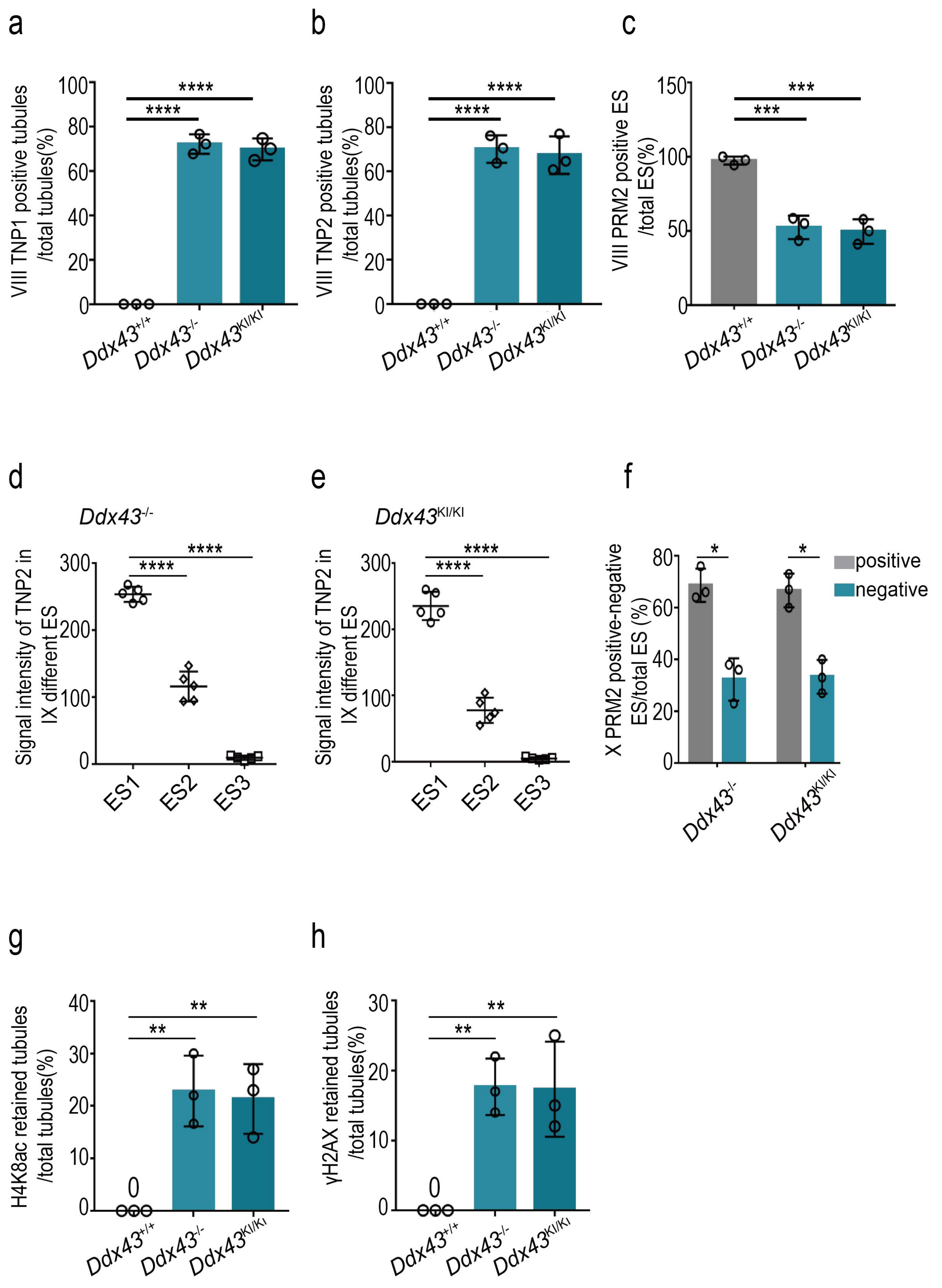

Supplementary Figure 6. Statistics of immunofluorescence analysis on

chromatin remodeling factors. Related to Figure 3.

(a, b) Percentage of TNP1 (a)- and TNP2 (b)-positive stage-VIII seminiferous tubules in all stage-XIII tubules from P60 *Ddx43*$^{+/+}$, *Ddx43*$^{KI/KI}$, and *Ddx43*$^{–/–}$ testes. Each bar represents the mean ± SD from biological triplicates. *P* values were calculated by oneway ANOVA (*****P* < 0.0001). **(c)** Percentage of PRM2-positive stage-VIII elongating spermatids (ES) in all stage-VIII ES from P60 *Ddx43*$^{+/+}$, *Ddx43*$^{KI/KI}$ and *Ddx43*$^{–/–}$seminiferous tubules. Each bar represents the mean ± SD from biological triplicates. *P* values were calculated by oneway ANOVA. *** *P* = 0.0005 (*Ddx43*$^{+/+}$ vs *Ddx43*$^{–/–}$), *** *P* = 0.0003 (*Ddx43*$^{+/+}$ vs *Ddx43*$^{KI/KI}$). **(d, e)** Quantification of TNP2 signal intensity in stage-IX tubules from P60 *Ddx43*$^{–/–}$(d) and *Ddx43*$^{KI/KI}$ (e) testes. Signal intensity was compared between the TNP2-positive cells described in the main text as conspicuous (ES1), weak (ES2), and absent (ES3). Each bar represents the mean ± SD from biological triplicates. *P* values were calculated by oneway ANOVA (*****P* < 0.0001). **(f)** Percentage of PRM2-positive and -negative stage-X ES in all stage-X ES from P60 *Ddx43*$^{–/–}$ and *Ddx43*$^{KI/KI}$ seminiferous tubules. Each bar represents the mean ± SD from biological triplicates. *P* values were calculated by two-tailed paired Student's t-test. **P*=0.0488 (*Ddx43*$^{–/–}$), **P*=0.0472 (*Ddx43*$^{KI/KI}$). **(g, h)** Percentage of seminiferous tubules with retained ES that were H4K8ac (g)-positive after step 11 and γH2AX (h)-positive after step 12, respectively, in all tubules from P60 *Ddx43*$^{+/+}$, *Ddx43*$^{KI/KI}$ and *Ddx43*$^{–/–}$ testes. Each bar represents the mean ± SD from biological triplicates. *P* values were calculated by oneway ANOVA. ***P* = 0.0052 (*Ddx43*$^{+/+}$ vs *Ddx43*$^{–/–}$ for H4K8ac), ***P* = 0.0073 (*Ddx43*$^{+/+}$ vs *Ddx43*$^{KI/KI}$ for H4K8ac), ***P* = 0.0077 (*Ddx43*$^{+/+}$ vs *Ddx43*$^{–/–}$ for γH2AX), ***P* = 0.0084 (*Ddx43*$^{+/+}$ vs *Ddx43*$^{KI/KI}$ for γH2AX).

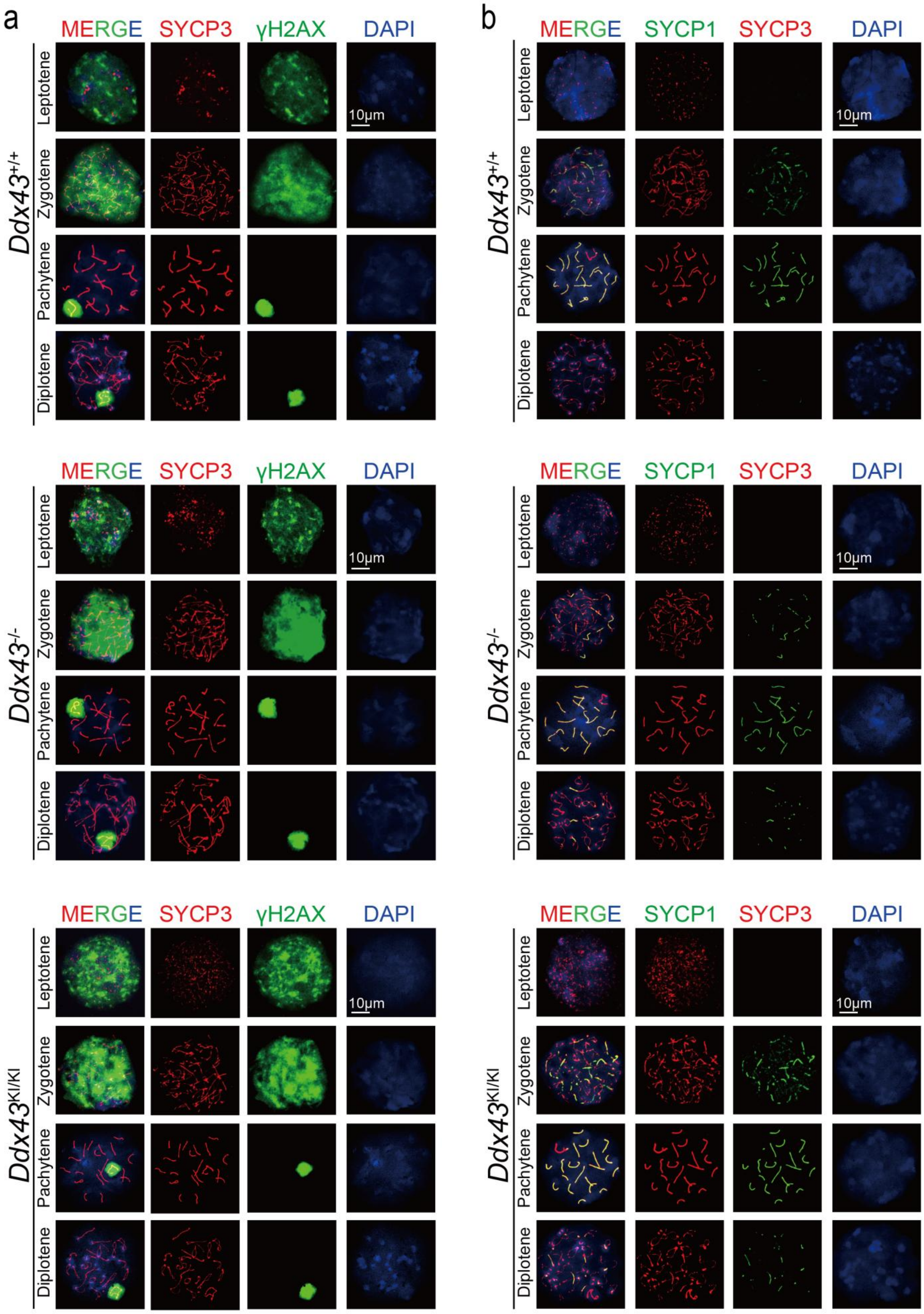

Supplementary Figure 7. Analyses of spermatocyte chromosome spread.

(a, b) Representative images of co-immunostaining of spermatocyte spread nuclei from *Ddx43*$^{+/+}$, *Ddx43*$^{KI/KI}$ and *Ddx43*$^{-/-}$ adult testes across four different stages (leptotene, zygotene, pachytene and diplotene) of meiotic prophase I. **(a)** γH2AX (green) and SYCP3 (red) show dynamic patterns of meiotic DNA double strand breaks, which emerge and spread over the leptotene spermatocytes, persist into zygotene stage, reduce in early pachytene stage, and are confined to the XY body in mid-late pachynema and diplonema. DNA was counterstained with DAPI. Scale bar are indicated. **(b)** Co-immunostaining of SYCP1 (green) and SYCP3 (red) shows the developmental process of homologous chromosome synapsis, which initiates at the zygotene stage and completes at the onset of the pachytene stage. Thus, all autosome regions can be immunolabeled by SYCP1 and SYCP3, which are otherwise confined within the short pseudoautosomal region of the sex chromosomes. DNA was counterstained with DAPI. Scale bar are indicated. Each experiment was repeated three times with similar results.

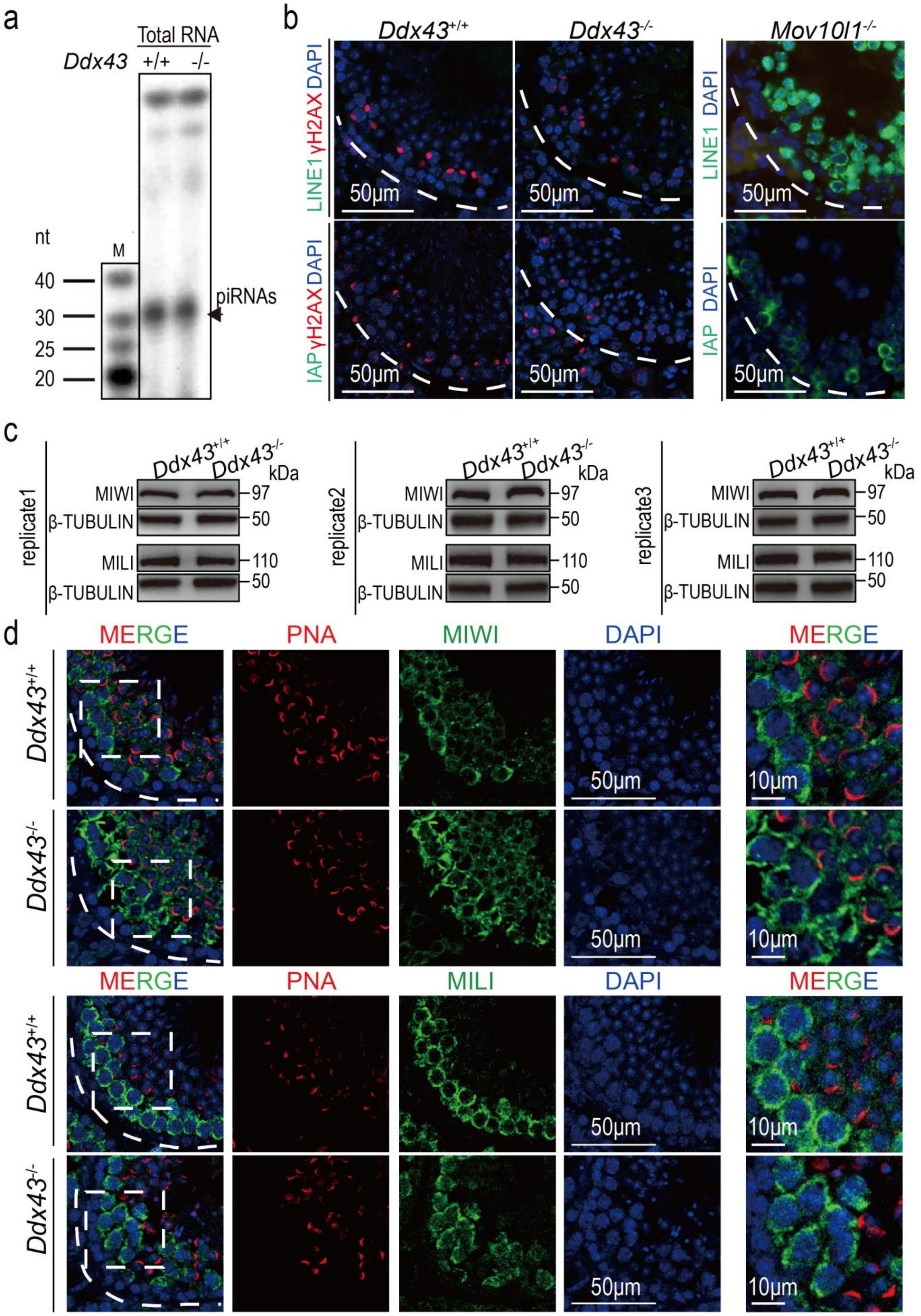

Supplementary Figure 8. Inspection of the piRNA pathway in testis.

(a) Pachytene piRNA abundance is not reduced in mutant testes from adult *Ddx43*$^{-/-}$ mice. ^{32}P-end-labelled total RNAs are separated for autoradiography by denaturing polyacrylamide gel electrophoresis. **(b)** Immunofluorescence detection of LINE1 (anti-L1 ORF1p antibody) and IAP (anti-IAP antibody) from *Ddx43*$^{+/+}$ and *Ddx43*$^{-/-}$ P60 testes. *Mov10l1*$^{-/-}$ testis sections were immunostained as a positive control. Green, LINE or IAP; red, γH2AX. DNA was counterstained with DAPI. Scale bars are indicated. **(c)** Western blot analyses of MIWI and MILI in lysates from P60 *Ddx43*$^{+/+}$ and *Ddx43*$^{-/-}$testes. β-TUBULIN serves as a loading control. **(d)** Immunofluorescence analyses of piRNA pathway components MIWI and MILI in *Ddx43*$^{+/+}$ and *Ddx43*$^{-/-}$ testes. Green, MIWI or MILI; red, PNA. DNA was counterstained with DAPI. Scale bars are indicated. Each experiment was repeated three times with similar results.

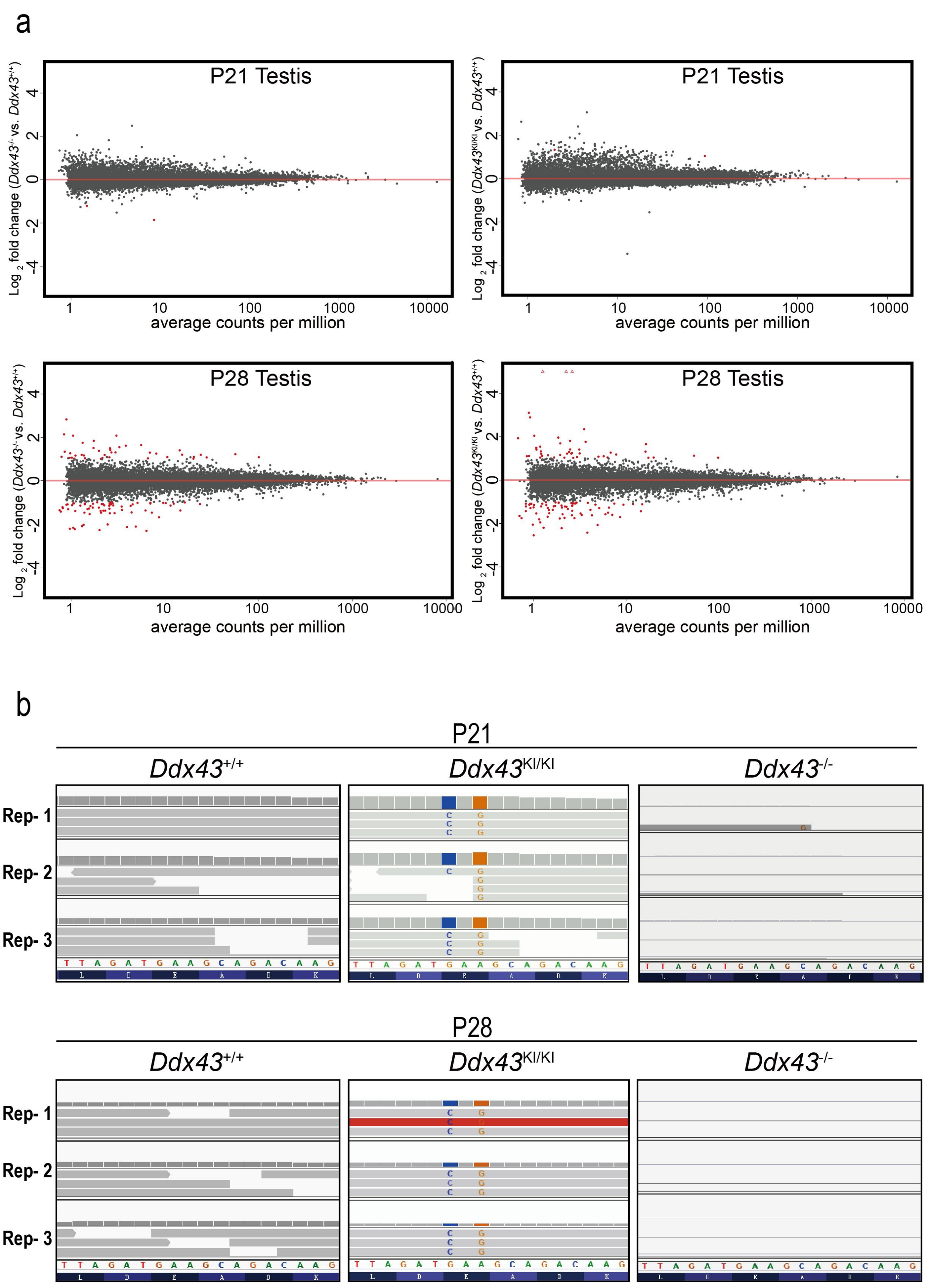

Supplementary Figure 9. RNA-seq analyses of testicular transcriptome.

(a) Scatter plot of differentially expressed transcripts in *Ddx43* mutant testes at P21 (upper) and P28 (lower) compared with age-matched *Ddx43*$^{+/+}$ testes. Genes showing altered expression with FDR adjusted *P* value < 0.05 (two-sided test) and absolute log 2 (fold change) > 1 changes are colored red. **(b)** Interactive Genome Viewer screenshot of a representative sample of reads mapping at the *Ddx43* gene locus.

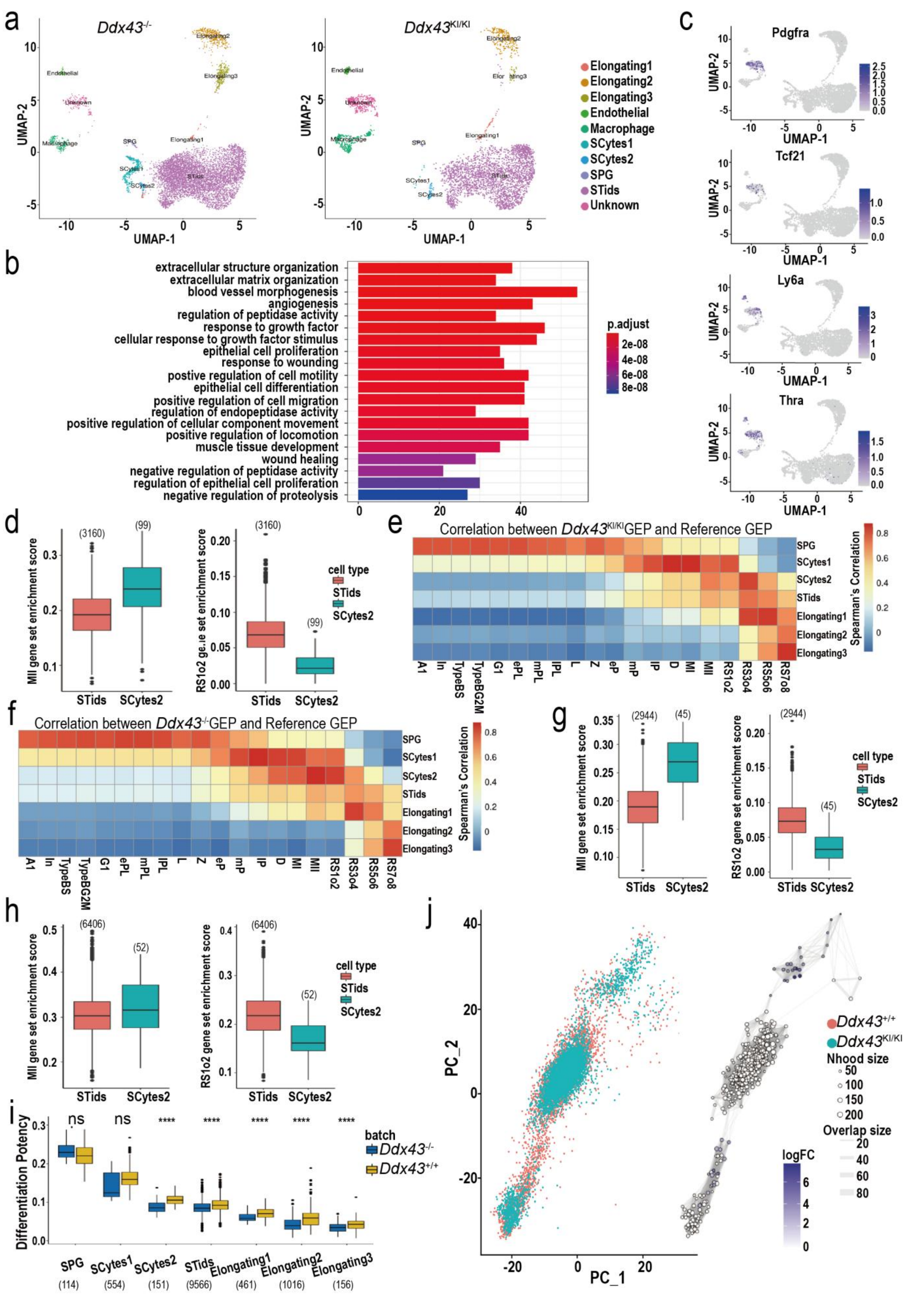

Supplementary Figure 10. Other characteristic analyses of scRNA-Seq in *Ddx43*$^{+/+}$ and *Ddx43* mutant testes. Related to Figure 4.

(a) UMAP and clustering analysis of combined single-cell transcriptome from mice *Ddx43*$^{–/–}$ and *Ddx43*$^{KI/KI}$ testes. Each dot represents a single cell and is colored according to its cluster identity as indicated on the figure key. **(b)** GO enrichment results of highly expressed gene in “Unknown” cell type. One-sided Fisher’s exact test. **(c)** Marker genes of “Unknown” cell type. **(d, g, h)** Box plot of metagene expression in SCytes2 and STids in *Ddx43*$^{+/+}$ (d), *Ddx43*$^{KI/KI}$ (g) and *Ddx43*$^{–/–}$ (h). The gene sets are curated from previous differential expression study between MII and Spermatids stage1 to stage 2[1]. The metagene expression values are determined through AUCell[2]. In the boxplots, the center line, box limits and whiskers denote the median, upper and lower quartiles and 1.5 × interquartile range, respectively. **(e, f)** The heatmap of our annotated *Ddx43*$^{KI/KI}$ (e) and *Ddx43*$^{–/–}$ (f) single-cell expression profile and the published flow-sorted single cell gene expression profile in cell type gene expression spearman correlation. **(i)** The boxplot of cell differentiation potency for each differentiation stages during spermatogenesis of both *Ddx43*$^{+/+}$ and *Ddx43*$^{–/–}$ mice. Asterisks indicate statistical significance: **** denotes *P* value ≤ 0.0001; ns, not significant; two-sided Wilcoxon rank-sum test. In the boxplots, the center line, box limits and whiskers denote the median, upper and lower quartiles and 1.5 × interquartile range, respectively. **(j)** A neighborhood graph of the results from Milo differential abundance testing (right panel). Nodes are neighborhoods, colored by their log fold change across genotypes. Non-differential abundance neighborhoods (*P* value > 0.05) are colored white, and sizes correspond to the number of cells in each neighborhood. Graph edges depict the number of cells shared between neighborhoods. The layout of nodes is determined by the position of the neighborhood index cell in the PCA (left panel).

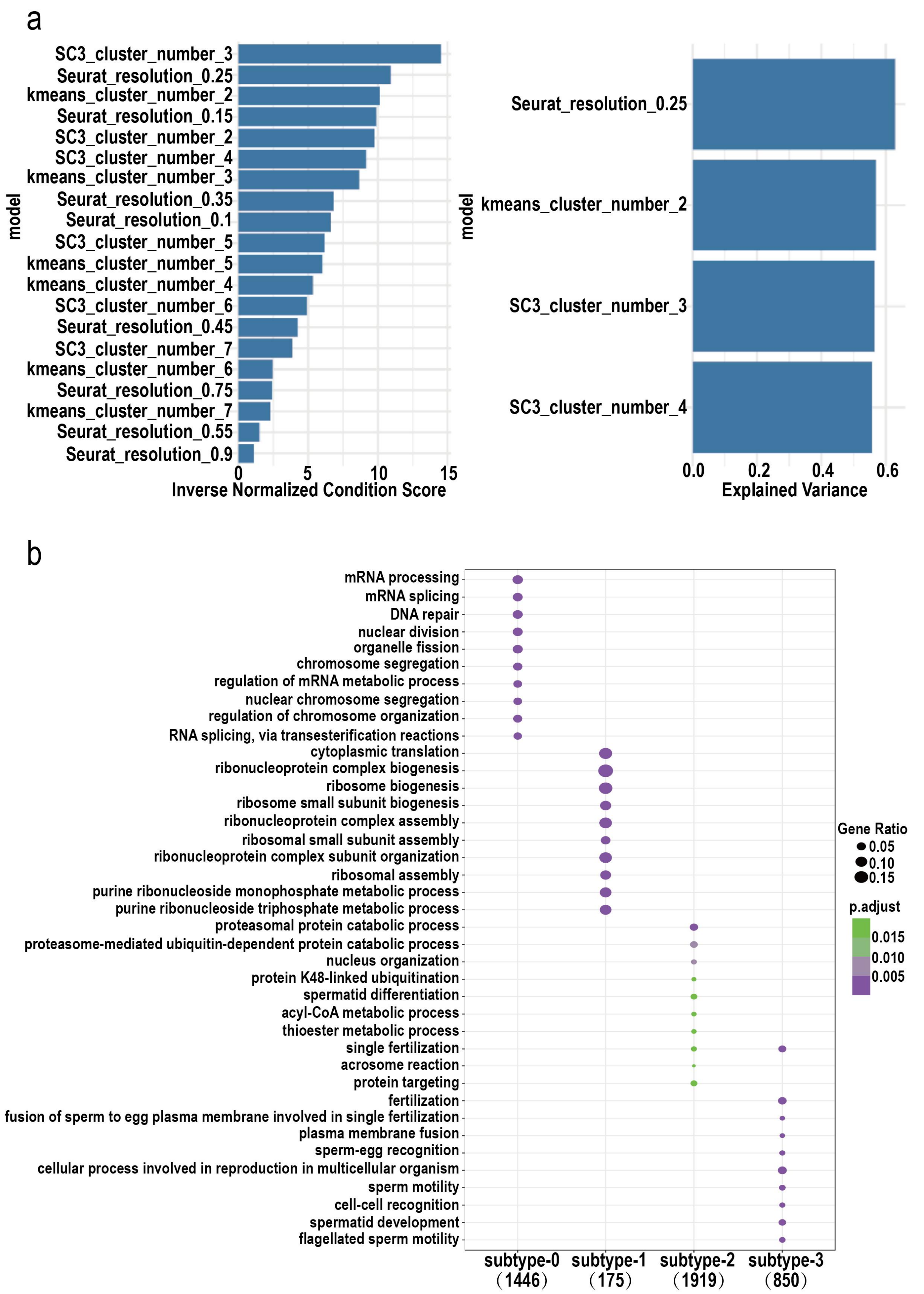

Supplementary Figure 11. Characterization of gene expression pattern of four

cellular states in STids. Related to Figure 5.

(a) Benchmark different clustering methods using Inverse Normalized Condition Number Score (left panel) and Explained Variance Score (right panel). **(b)** GO enrichment of the marker genes of four cellular states of Stids (one-sided Fisher's exact test).

a

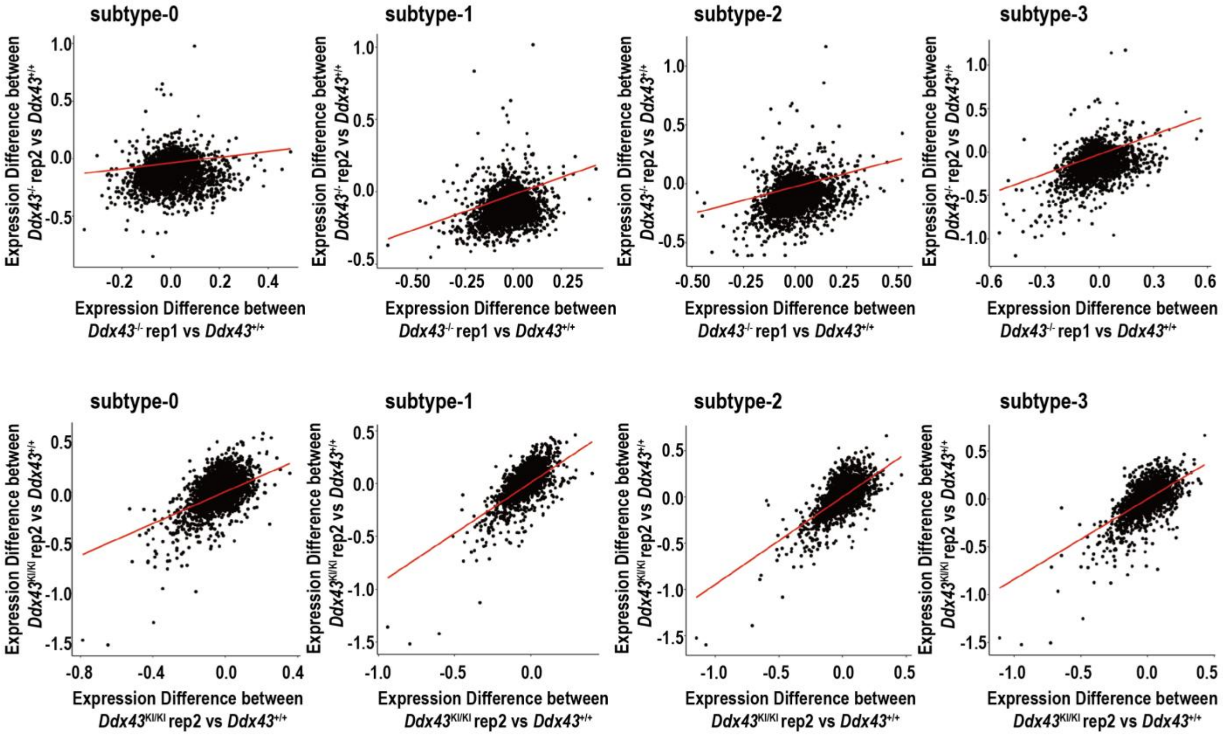

b

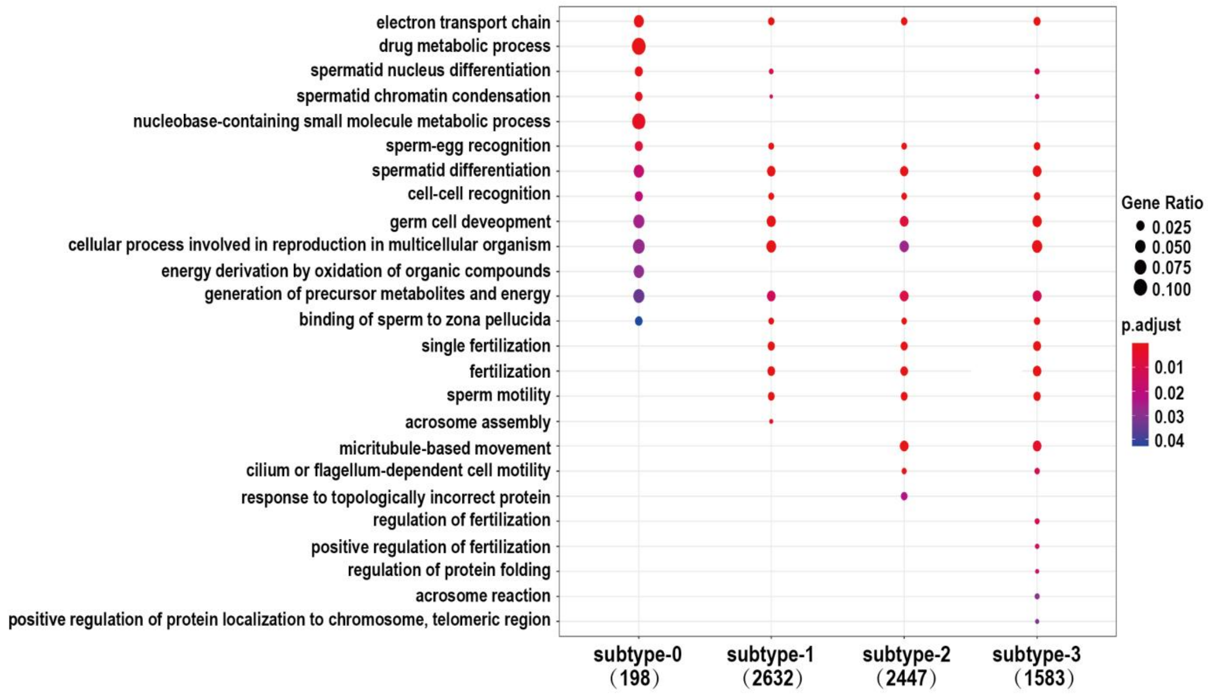

Supplementary Figure 12. DEG analysis between *Ddx43*$^{+/+}$ and *Ddx43* mutants.

(a) Scatter plot of the Fold Change values of all genes in four sub-clusters. For each *Ddx43* mutant genotype we have two replicates. For each replicate, we performed DEG analysis with the wild-type in four sub-clusters respectively, calculated the fold change values, and then assessed the consistency of the Fold Change values between two replicates. **(b)** GO enrichment of the differential expression genes of four cellular states of STids (one-sided Fisher's exact test).

a

eCLIP sequencing library processing summary metrics

Sample		Raw reads	short reads	pcr_dup	clean reads	Total clean reads	Total mapped	Total Uniquely mapped	Total Multiple mapped
SMInput1	50999456	1684654 (3.30%)	1684654 (3.30%)	11220851 (22.00%)	38093951 (74.69%)	75818467	61133433 (80.63%)	40448651 (66.16%)	20684782 (33.84%)
	50638651	2410635 (4.76%)	2410635 (4.76%)	10503500 (20.74%)	37724516 (74.50%)				
DDX43 IP1	56004349	2746478 (4.90%)	2746478 (4.90%)	13709401 (24.48%)	39548470 (70.62%)	75452592	50062926 (66.35%)	41894388 (83.68%)	8168538 (16.32%)
	55210178	4103965 (7.43%)	4103965 (7.43%)	12659743 (22.93%)	38446470 (69.64%)				
SMInput2	50868017	1706389 (3.35%)	1706389 (3.35%)	10584828 (20.81%)	38576800 (75.84%)	77994940	61447833 (78.78%)	40243788 (65.49%)	21204045 (34.51%)
	50770778	4029196 (7.94%)	4029196 (7.94%)	9865790 (19.43%)	36875792 (72.63%)				
DDX43 IP2	56964723	1504015 (2.64%)	1504015 (2.64%)	18107359 (31.79%)	37353349 (65.57%)	69684187	48439019 (69.51%)	39981788 (82.54%)	8457231 (17.46%)
	56825817	3833629 (6.75%)	3833629 (6.75%)	11981027 (21.08%)	41011161 (72.17%)				

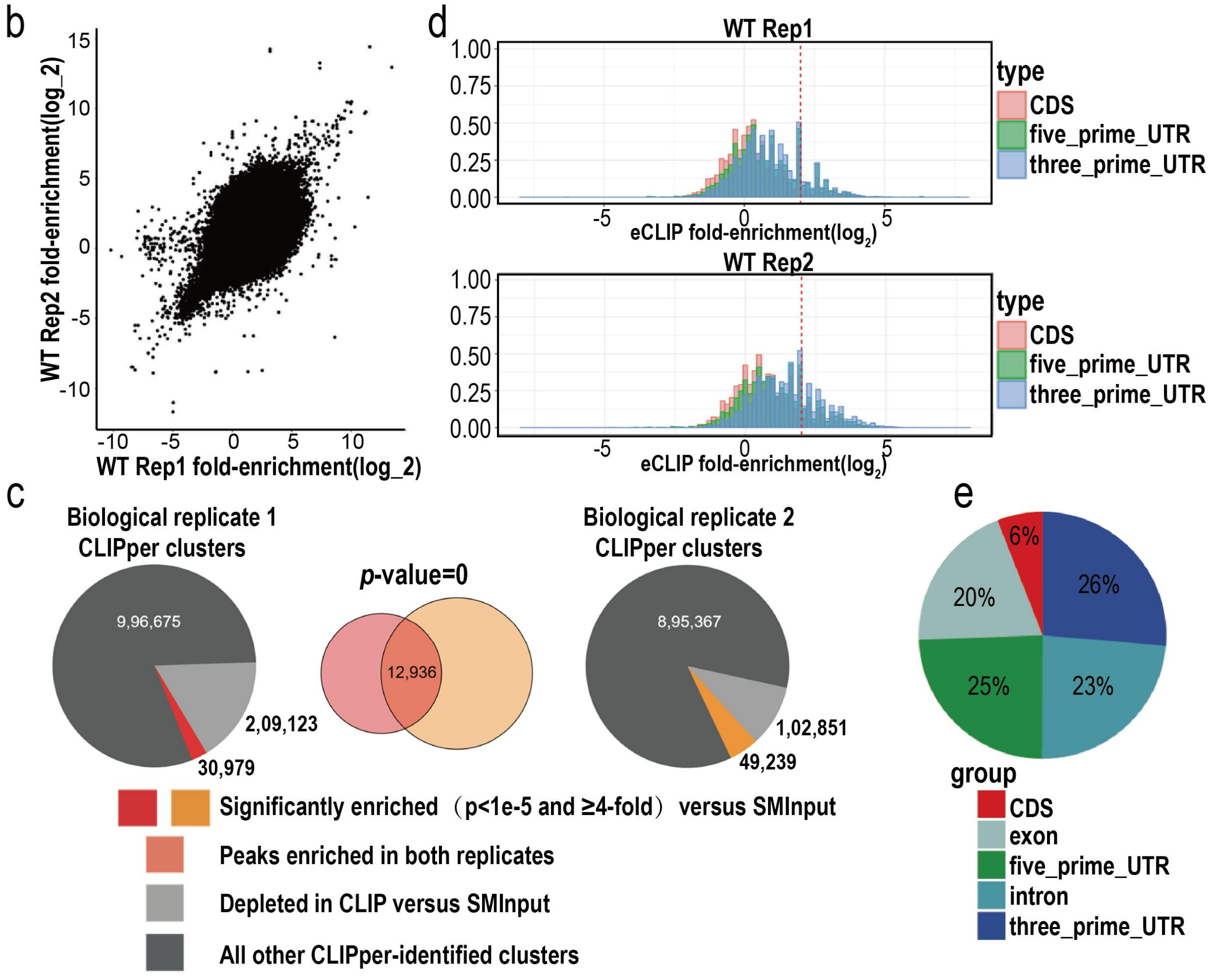

Supplementary Figure 13. Genome-wide mapping of DDX43 eCLIP-seq. Related

to Figure 5.

(a) A table related to eCLIP-seq library processing summary metrics. **(b)** Scatter plot indicates correlation between DDX43 biological replicates based on the fold enrichment. **(c)** For each biological replicate, CLIPper identifies clusters of enriched read density within DDX43 eCLIPs. Based on cluster read density comparisons between eCLIP and paired SMInput, we identified subsets of clusters enriched above SMInput (red/orange), which display high overlap between replicates (center). The significance of overlapping is tested through two-sided Fisher's exact test. **(d)** Histogram of region-based fold enrichment for DDX43 with paired SMInput as comparison. **(e)** Pie chart showing the distribution of significantly enriched replicate peaks for DDX43 eCLIP-seq data.

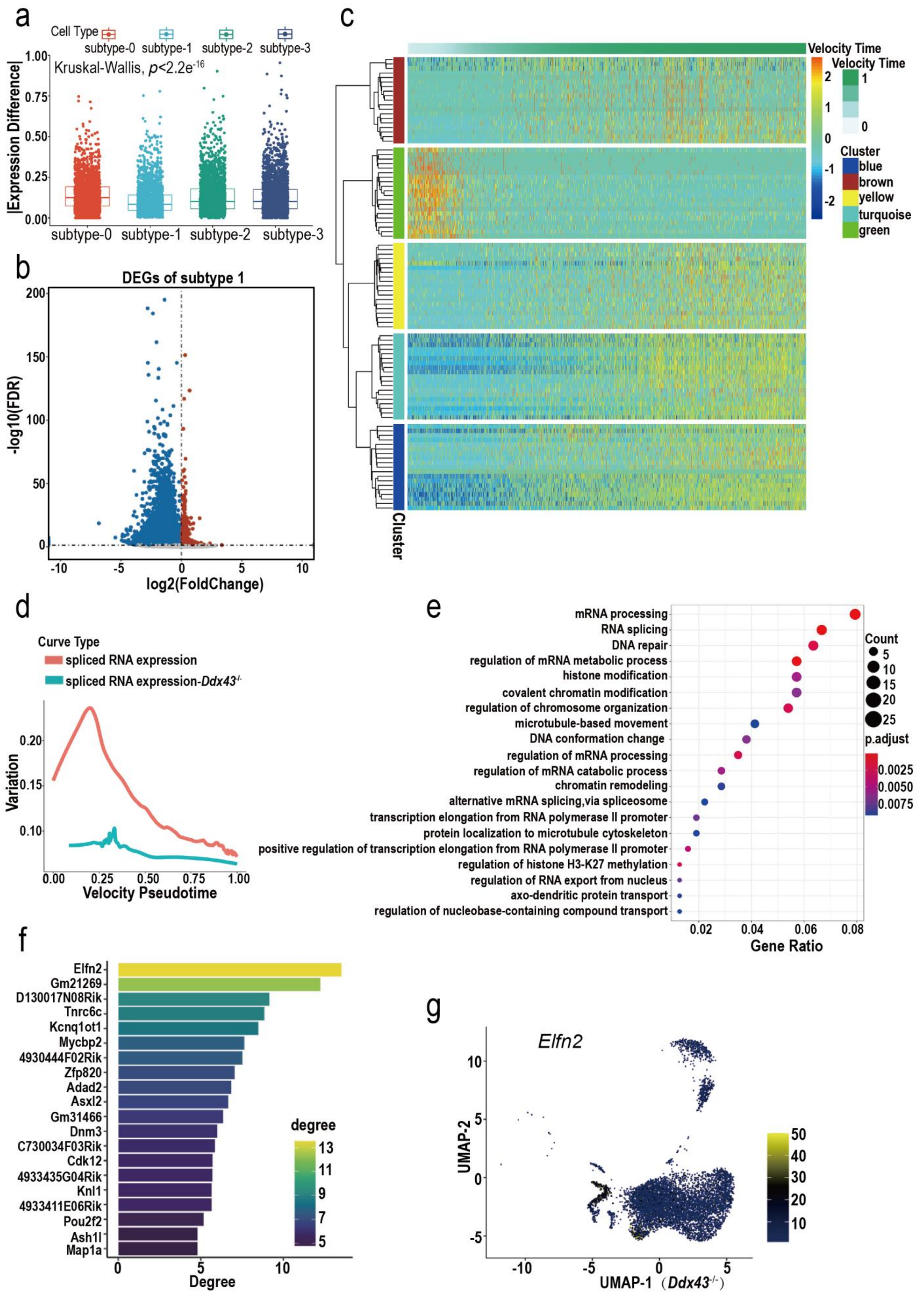

Supplementary Figure 14. Dynamic network analyses using *Ddx43*$^{-/-}$ and

wild-type samples. Related to Figure 5 and Figure 6.

(a) The boxplot for absolute expression difference value of DDX43 differential expression targets (DETs) in four cellular states of STids of *Ddx43*$^{-/-}$ samples. In the boxplots, the center line, box limits and whiskers denote the median, upper and lower quartiles and 1.5 × interquartile range, respectively. **(b)** Volcano plot showing gene differential expression (*Ddx43*$^{+/+}$ vs *Ddx43*$^{-/-}$) of subtype-1 (two-sided Wilcoxon rank-sum test). **(c)** WGCNA clustering of genes exhibiting down-regulated expression in subtype-1 of *Ddx43*$^{-/-}$ samples. Each row represents a gene, and each column represents a single cell, with columns/cells placed in velocity pseudotime order and depicted by a thick colored line (top). Gene expression levels utilize a Z score transformation. **(d)** Line plot of green module activity along with velocity pseudotime, module activity is calculated using AUCell. **(e)** GO analysis of the genes in green module of *Ddx43*$^{-/-}$ samples (one-sided Fisher's exact test). **(f)** Barplot of the network connectivity of driver candidates. Each bar is colored according to each gene connectivity in the network, which reflects the gene's importance to the biological systems. **(g)** Expression patterns of *Elfn2* in *Ddx43*$^{-/-}$ mutant.

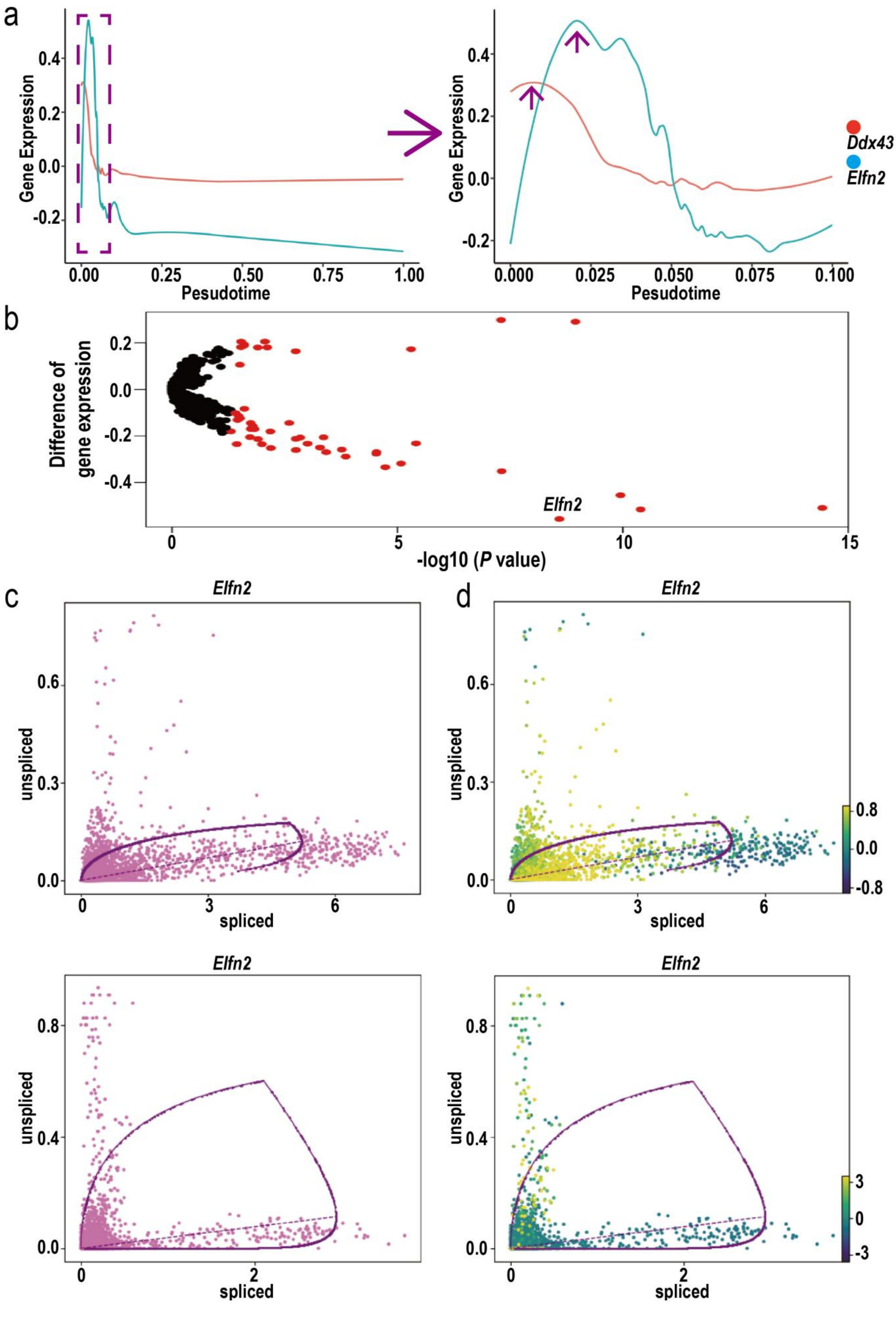

Supplementary Figure 15. Identifying *Elfn2* as the key target of DDX43. Related to

Figure 6.

(a) Line plot of *Elfn2* and *Ddx43* expression along the velocity pseudotime. Zooming in the early stage (left panel), we found *Ddx43* expression earlier than *Elfn2* (right panel). **(b)** Volcano plot showing DDX43 differential expression targets (DETs) in subtype-0. Red dots indicate the DETs that test significantly differential expressed in subtype-0 (two-sided Wilcoxon rank-sum test, FDR adjusted *P* value < 0.05). **(c, d)** The gene life cycle of *Elfn2* in *Ddx43*$^{+/+}$ (c) and *Ddx43*$^{KI/KI}$ (d) cells. Each dot represents a cell colored according to the cell types (c) or velocity values (d). Here we only extracted STids for visualization. The x-axis represents the spliced RNA expression level； the y-axis represents the unspliced RNA expression level.

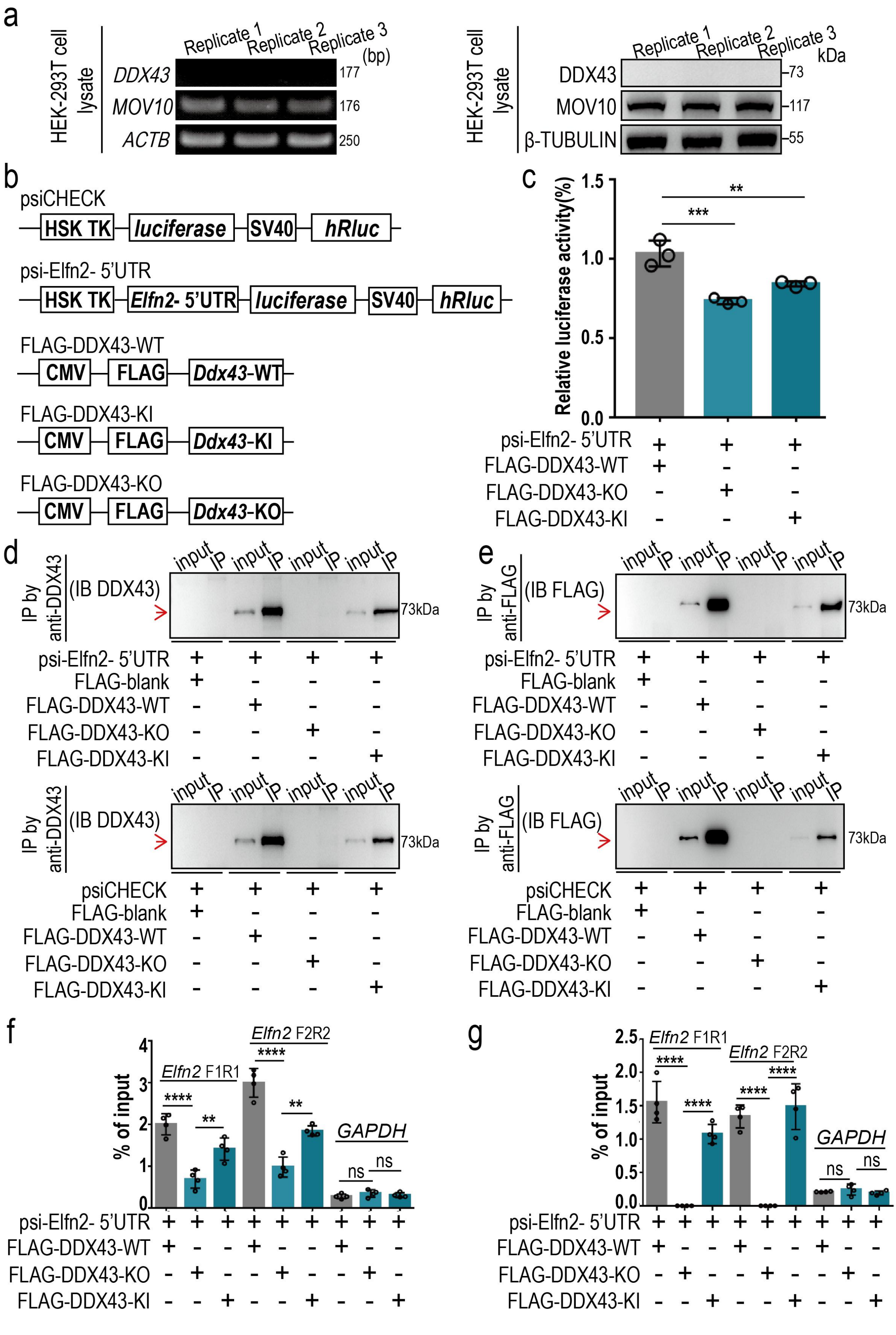

Supplementary Figure 16. DDX43 protein acts on *Elfn2* 5' UTR.

(a) The assessment of endogenous *DDX43* mRNA (left) and DDX43 protein (right) expression in HEK293 cells. *ACTB* and β-TUBULIN serve as controls. **(b)** Schematic representation of plasmids used for co-transfection in HEK293T cells. **(c)** HEK293T cells were co-transfected with the plasmids shown in panel (b) for Dual luciferase reporter assay. The ratio of Firefly and Renilla luciferase activities were calculated. Cells transfected with psiCHECK and FLAG-blank were used for normalization. Each bar represents the mean ± SD for biological triplicates. *P* values were calculated by oneway ANOVA. ****P* = 0.0008, ***P* = 0.0076. **(d, e)** DDX43-RNA complexes were isolated from lysates of co-transfected cells by immunoprecipitation (IP) using anti-DDX43 (d) or anti-FLAG (e) antibodies. DDX43 proteins in the immunoprecipitated complexes were examined by immunoblotting (IB) using anti-DDX43 (d) or anti-FLAG (e) antibodies. Each experiment was repeated three times with similar results. **(f, g)** DDX43-bound *Elfn2* transcripts in the immunoprecipitated complexes using anti-DDX43 (f) or anti-FLAG (g) antibodies were examined by qRT-PCR analyses. Two pairs of PCR primers (F1R1, F2R2) were used to test *Elfn2* 5' UTR. Percentage of input is used to calculate binding. *P* values were calculated by oneway ANOVA. *****P* < 0.0001, ***P* = 0.0067 (*Elfn2* F1R1), ***P* = 0.0024 (*Elfn2* F2R2), ns, not significant. Each bar represents the mean ± SD for biological triplicates.

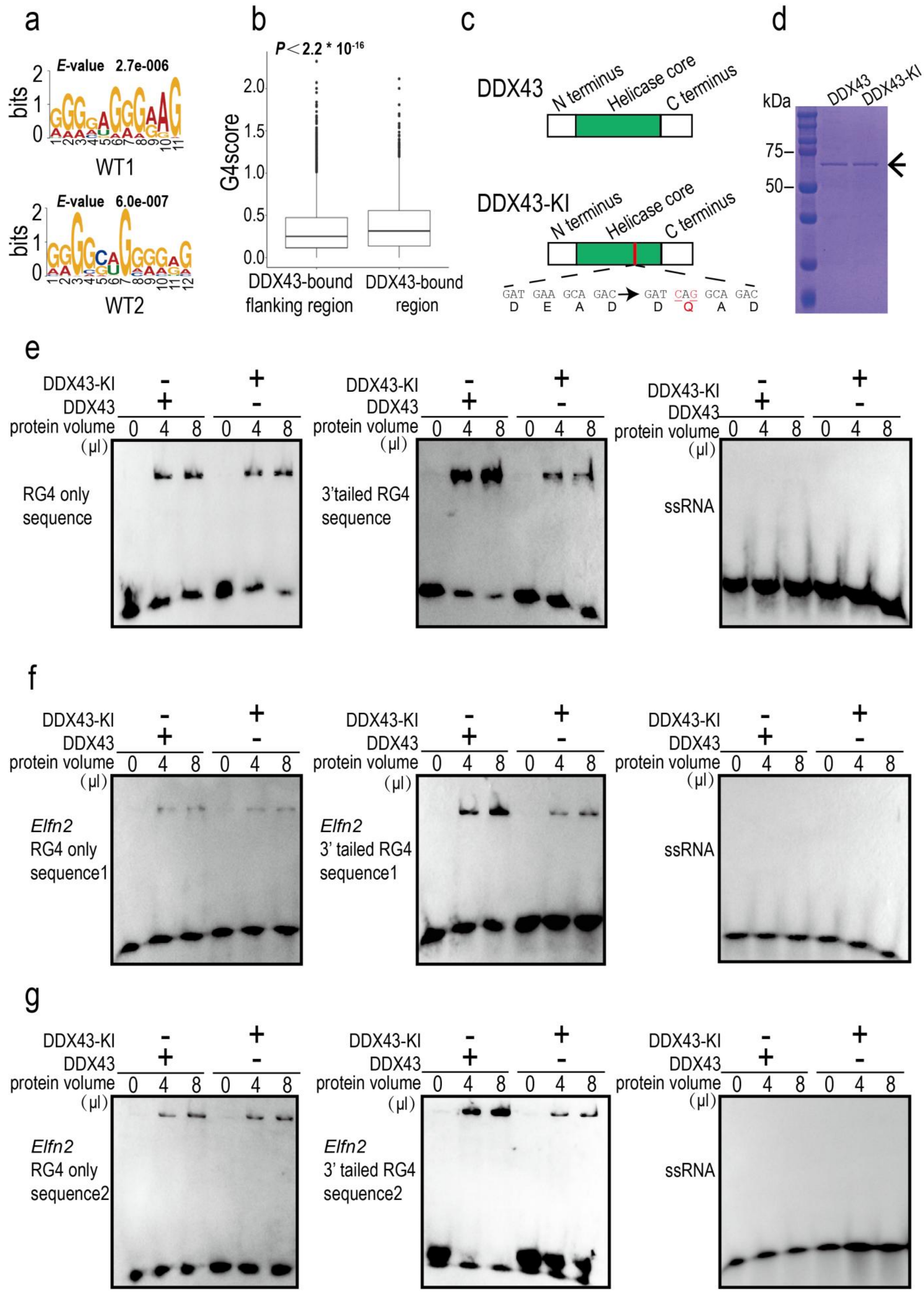

Supplementary Figure 17. DDX43 protein binds RNA G-quadruplex (RG4)

(a) DDX43-binding motifs identified by MEME analysis from the top 50% of DDX43 eCLIP peaks. The two biological replicates showed similar results. The *E* value is an estimation of the expected number of motifs with the given log likelihood ratio (or higher), and with the same width and site count. **(b)** Box plot showing the RG4 preference in DDX43-bound targets from eCLIP-seq. In the boxplot, the center line, box limits and whiskers denote the median, upper and lower quartiles and 1.5 × interquartile range, respectively. Two-sided Wilcoxon rank-sum test is used to test significance. **(c)** Schematic of DDX43 and DDX43-KI proteins used for the experiments in vitro. **(d)** SDS-PAGE analysis of purified proteins of DDX43 and DDX43-KI. **(e-g)** Electrophoretic mobility shift assay (EMSA) of DDX43 and DDX43-KI proteins with one RG4 sequence (with or without 3' overhang) (e) and two RG4 sequence (with or without 3' overhang) identified within *Elfn2* 5' UTR (f, g). Single-stranded RNA (ssRNA) serves as a comparison with RG4. Each experiment was repeated three times with similar results.

Fig. 1a

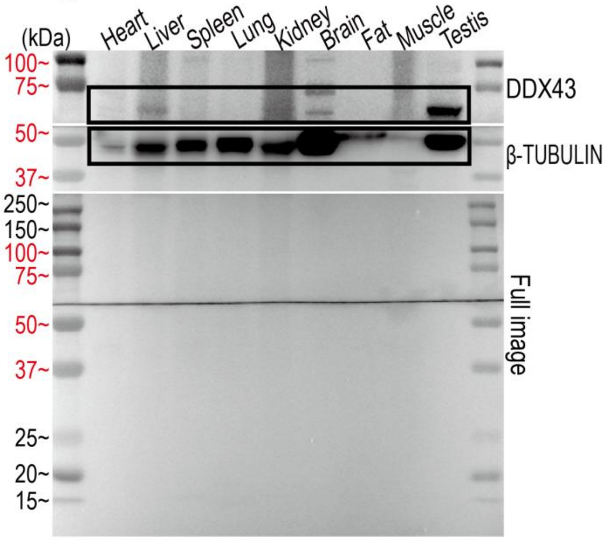

Fig. 1b

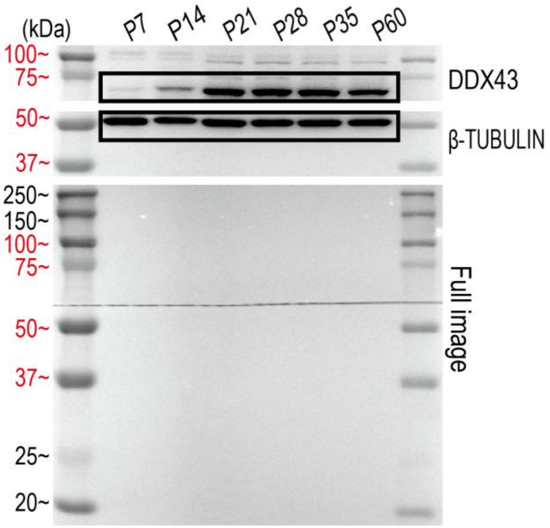

Fig. 1c

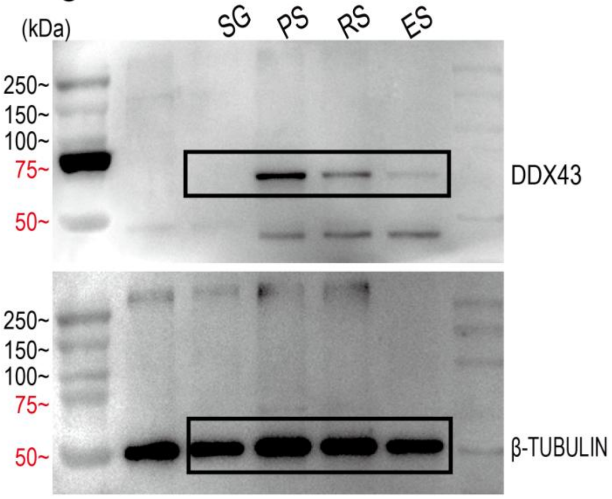

Fig. 1h

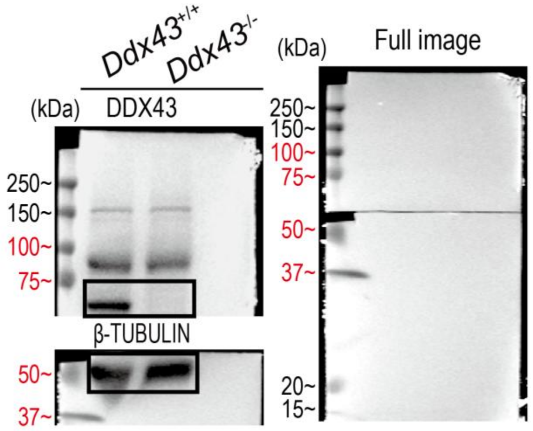

Fig. 7a

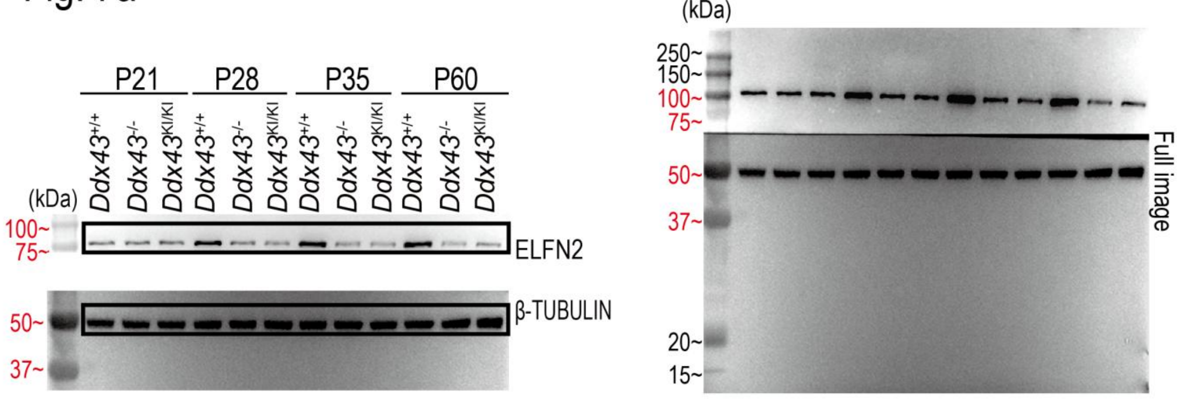

Fig. 7b

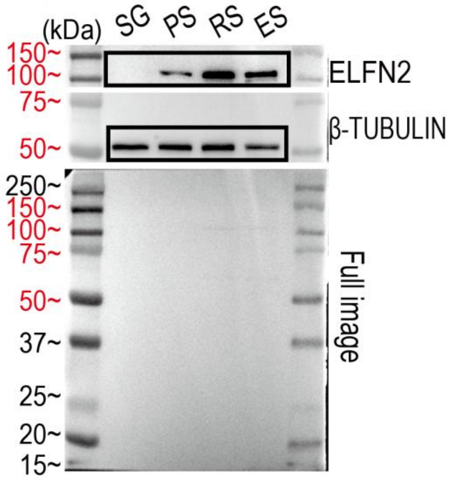

Fig. 7e

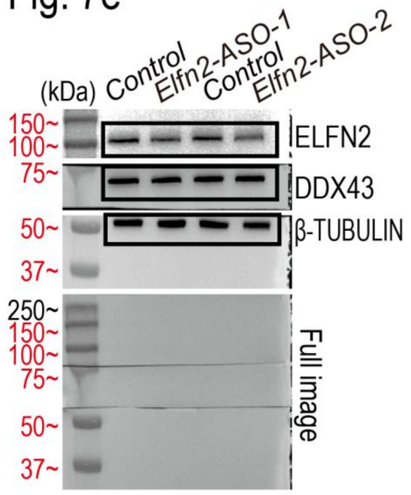

Supplementary Fig. 1g

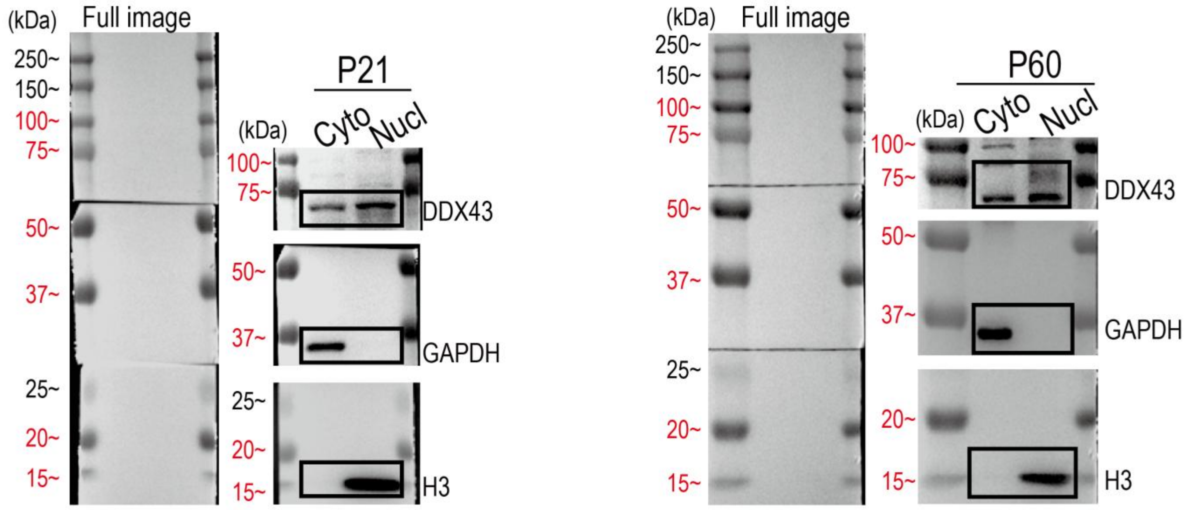

Supplementary Fig. 3d

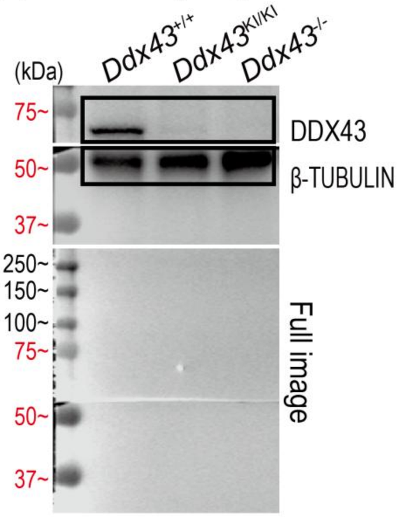

Supplementary Fig. 3f

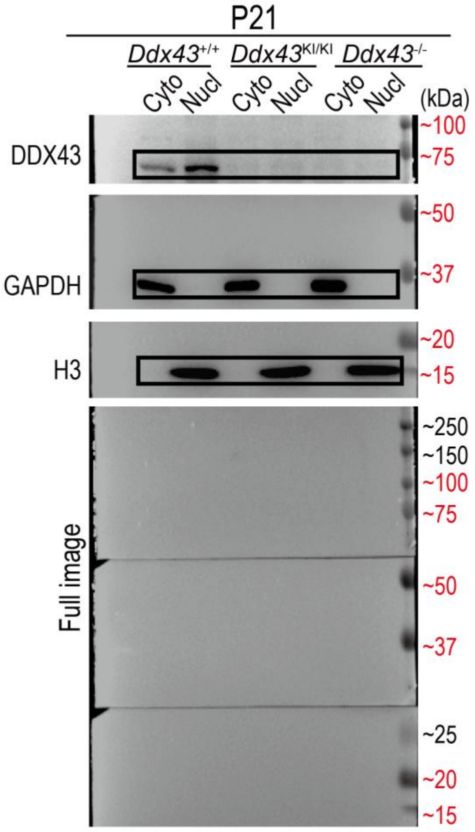

Supplementary Fig. 3g

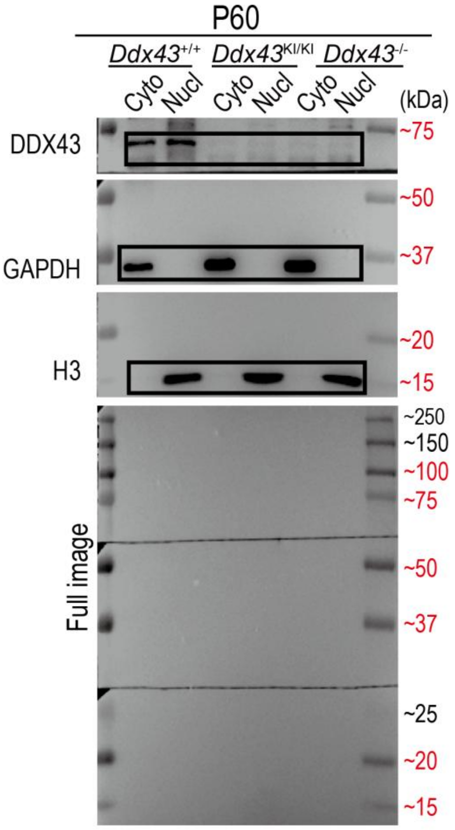

Supplementary Fig. 8c

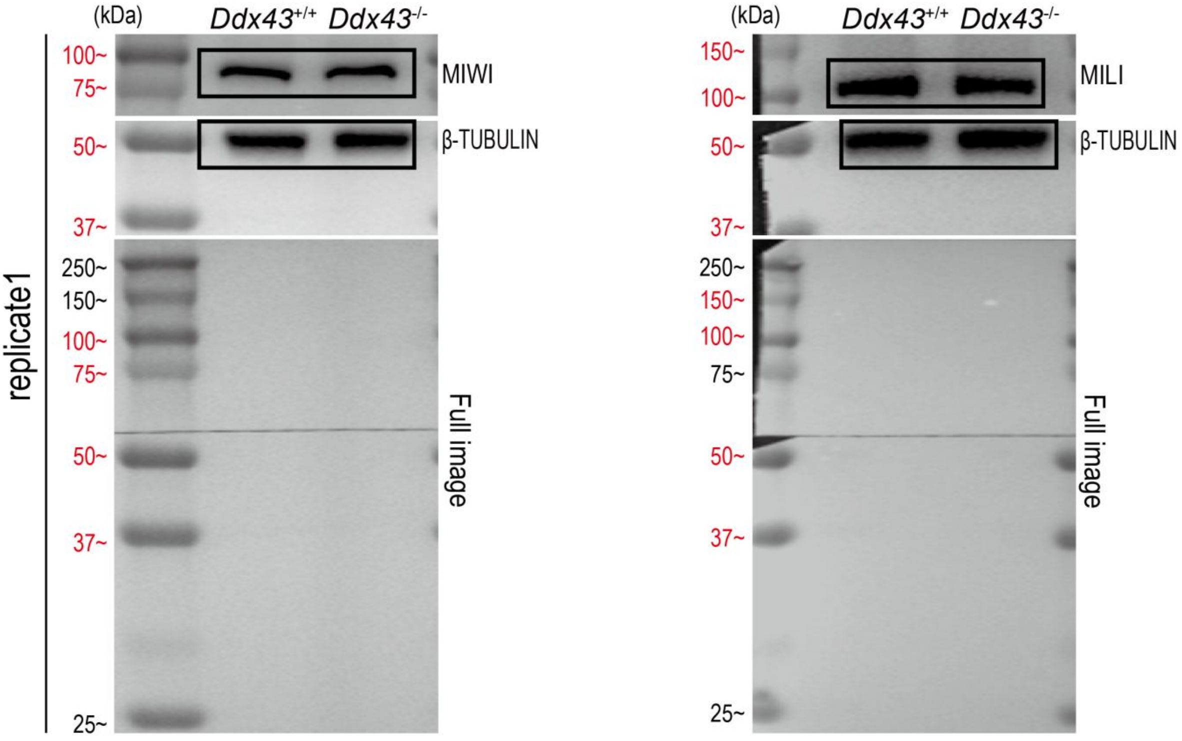

Supplementary Fig. 8c

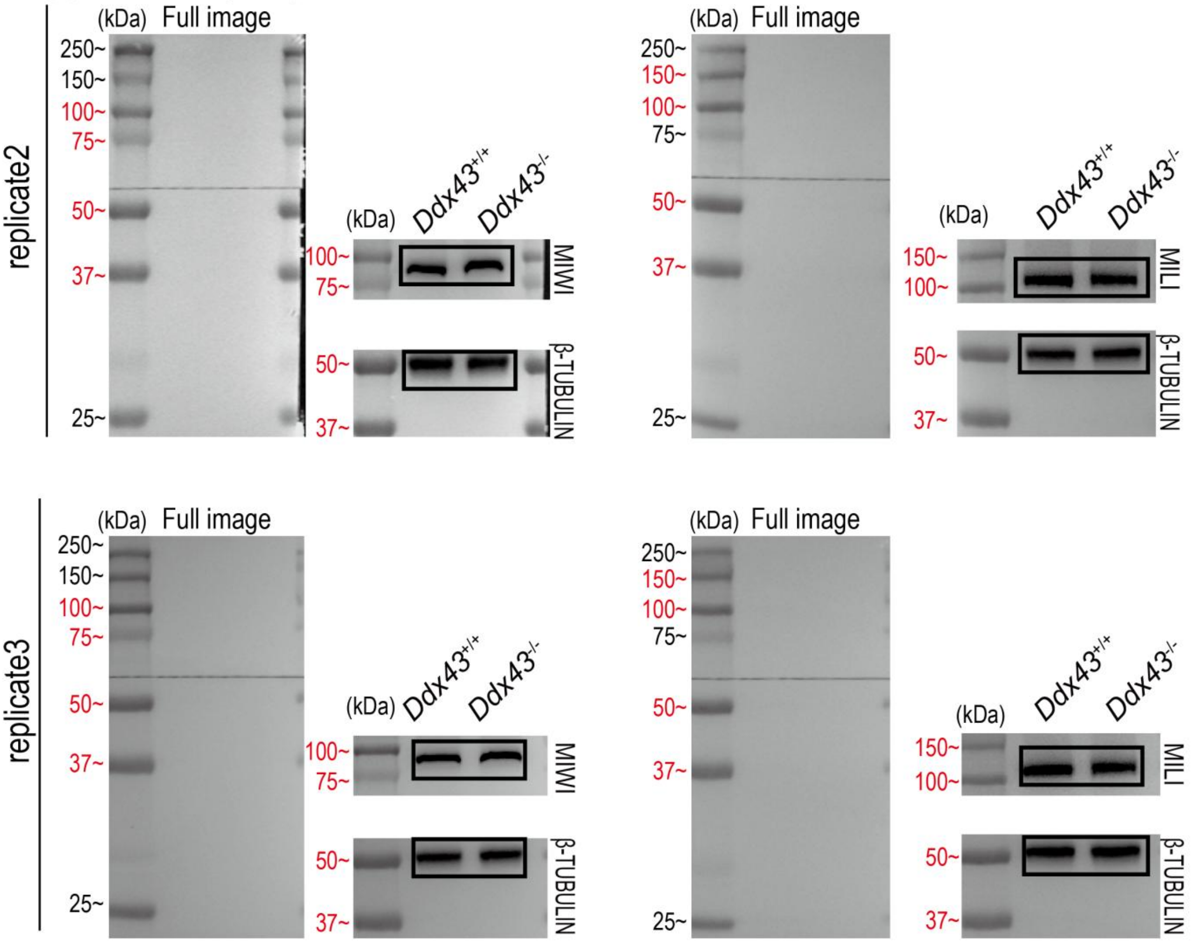

Supplementary Fig. 16a

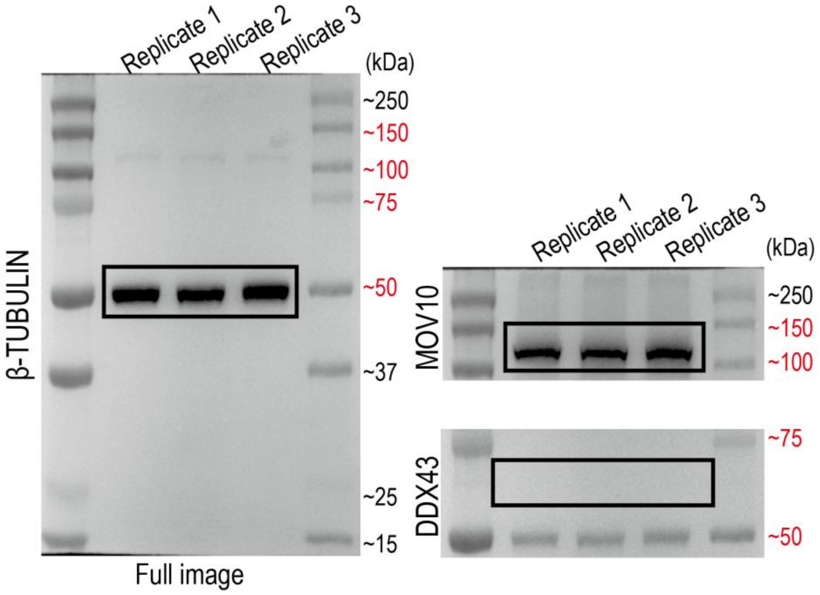

Supplementary Figure 18. Unprocessed images of immunoblotting

Immunoblotting was performed on PVDF membranes cut into various pieces for incubation with various antibodies. Black boxes indicate images showed in relevant figures.

Supplementary Fig. 1e

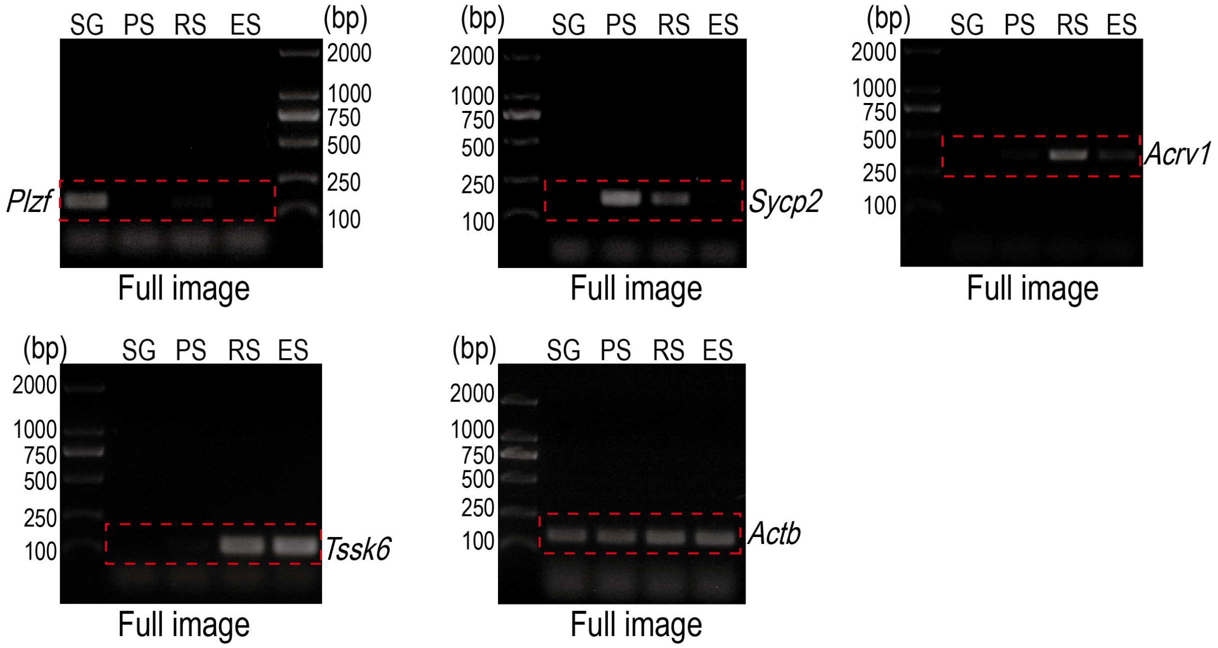

Supplementary Fig. 2c

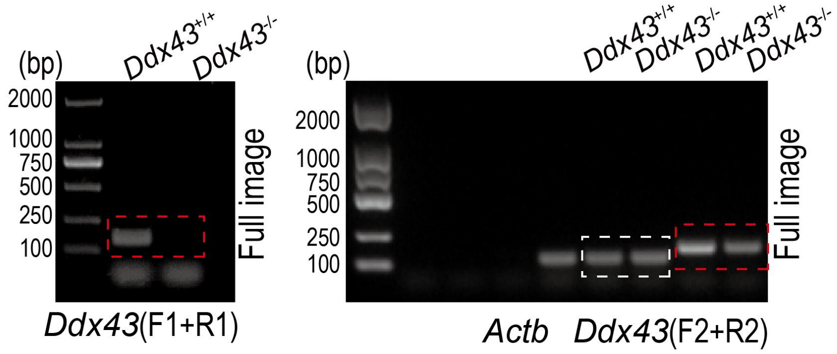

Supplementary Fig. 2d

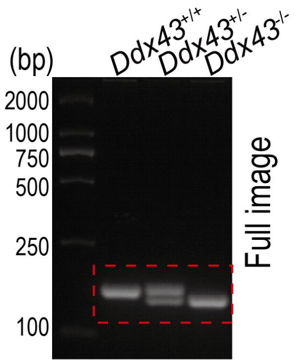

Supplementary Fig. 3c

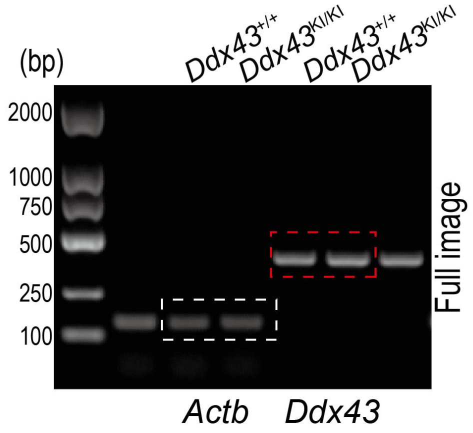

Supplementary Fig. 16a

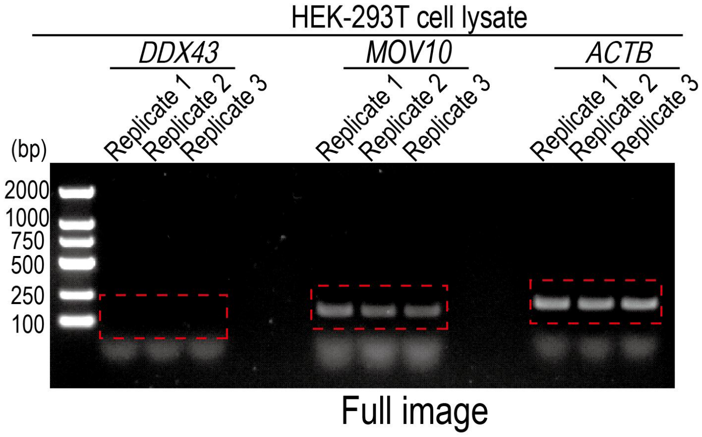

Supplementary Figure 19. Uncropped gels for the figures.

Uncropped gels for RT-PCR analyses. Red boxes and white boxes indicate images of target genes showed in relevant figures, and white boxes indicate images of *Actb.*

Supplementary References

1. Chen, Y. *et al.* Single-cell RNA-seq uncovers dynamic processes and critical regulators in mouse spermatogenesis. *Cell Res* **28**, 879-896 (2018).
2. Aibar, S. *et al.* SCENIC: single-cell regulatory network inference and clustering. *Nat Methods* **14**, 1083-1086 (2017).
