# Supplementary material for: Single-cell RNA-seq uncovers dynamic processes orchestrated by RNA-binding protein DDX43 in chromatin remodeling during spermiogenesis
Source: Nat Commun. 2023 Apr 29;14:2499. doi: 10.1038/s41467-023-38199-w (PMC10294715; doi:10.1038/s41467-023-38199-w)
Supplement: Supplementary file 2 — Description of Additional Supplementary Files [file 41467_2023_38199_MOESM2_ESM.pdf]

Description of Additional Supplementary Files

File Name: Supplementary Data 1
Description: All oligonucleotide sequences used in this study.
