# Supplementary material for: Single-cell RNA-seq uncovers dynamic processes orchestrated by RNA-binding protein DDX43 in chromatin remodeling during spermiogenesis
Source: Nat Commun. 2023 Apr 29;14:2499. doi: 10.1038/s41467-023-38199-w (PMC10294715; doi:10.1038/s41467-023-38199-w)
Supplement: Supplementary file 4 — Reporting Summary [file 41467_2023_38199_MOESM4_ESM.pdf]

nature portfolio

Corresponding author(s): Yiqiang Cui, Ting Ni, Ke Zheng

Last updated by author(s): 3/29/2023

Reporting Summary

Nature Portfolio wishes to improve the reproducibility of the work that we publish. This form provides structure for consistency and transparency in reporting. For further information on Nature Portfolio policies, see our Editorial Policies and the Editorial Policy Checklist.

Please do not complete any field with "not applicable" or n/a. Refer to the help text for what text to use if an item is not relevant to your study.
For final submission: please carefully check your responses for accuracy; you will not be able to make changes later.

Statistics

For all statistical analyses, confirm that the following items are present in the figure legend, table legend, main text, or Methods section.

n/a	Confirmed	
☐	☒	The exact sample size (*n*) for each experimental group/condition, given as a discrete number and unit of measurement
☐	☒	A statement on whether measurements were taken from distinct samples or whether the same sample was measured repeatedly
☐	☒	The statistical test(s) used AND whether they are one- or two-sided<br>*Only common tests should be described solely by name; describe more complex techniques in the Methods section.*
☒	☐	A description of all covariates tested
☐	☒	A description of any assumptions or corrections, such as tests of normality and adjustment for multiple comparisons
☐	☒	A full description of the statistical parameters including central tendency (e.g. means) or other basic estimates (e.g. regression coefficient) AND variation (e.g. standard deviation) or associated estimates of uncertainty (e.g. confidence intervals)
☐	☒	For null hypothesis testing, the test statistic (e.g. *F*, *t*, *r*) with confidence intervals, effect sizes, degrees of freedom and *P* value noted<br>*Give P values as exact values whenever suitable.*
☒	☐	For Bayesian analysis, information on the choice of priors and Markov chain Monte Carlo settings
☒	☐	For hierarchical and complex designs, identification of the appropriate level for tests and full reporting of outcomes
☐	☒	Estimates of effect sizes (e.g. Cohen's *d*, Pearson's *r*), indicating how they were calculated

Our web collection on statistics for biologists contains articles on many of the points above.

Software and code

Policy information about availability of computer code

Data collection

1. Cellranger
2. CLIPper
3. Tanon FLICapture (5200 chemiluminescent imaging sstem).
4. Carl Zeiss LSM800 confocal microscope
5. Image-Pro Plus 6.0
6. Zeiss Axio Scope A1
7. Tanon 2500 Fully automatic gel imaging system
8. BioTek Synergy 2 enzyme-labeled

Data analysis

The source code to reproduce main results in our study could be downloaded from http
MouseGerm_DDX43.
1. R 4.0.2
2. Package Seurat v4.1.0,
3. scVelo v0.2.5
4. package Scanpy v1.8.2.
5. ICAnet v0.0.0.9
6. SCENT v1.0.2

8. ggplot2 v3.3.6
9. WGCNA v1.70.3

10. Excel (Microsoft 2016)
11. ImageJ (NIH)
12. GraphPad Prism 6
13. Adobe Photoshop CC 2017
14. Illustrator CC 2017
15. QuantStudio™ Design & Analysis Software v14 (ABI Laboratories).

For manuscripts utilizing custom algorithms or software that are central to the research but not yet described in published literature, software must be made available to editors and reviewers. We strongly encourage code deposition in a community repository (e.g. GitHub). See the Nature Portfolio guidelines for submitting code & software for further information.

Data

Policy information about availability of data

All manuscripts must include a data availability statement. This statement should provide the following information, where applicable:

- Accession codes, unique identifiers, or web links for publicly available datasets
- A description of any restrictions on data availability
- For clinical datasets or third party data, please ensure that the statement adheres to our policy

The raw scRNA-seq datasets of mouse whole testis (fastq format) have been uploaded to NCBI-SRA with the accession number PRJNA650016. The raw eCLIP-seq and RNA-seq datasets of mouse whole testis (fastq format) have been uploaded to NCBI-SRA with the accession number PRJNA838233. Processed datasets are available at Zenodo with the accesss code DOI: https://doi.org/10.5281/zenodo.7479750.

Human research participants

Policy information about studies involving human research participants and Sex and Gender in Rese

Reporting on sex and gender	N/A
Population characteristics	N/A
Recruitment	N/A
Ethics oversight	N/A

Note that full information on the approval of the study protocol must also be provided in the manuscript.

Field-specific reporting

Please select the one below that is the best fit for your research. If you are not sure, read the appropriate sections before making your selection.

- [x] Life sciences
- [ ] Behavioural & social sciences
- [ ] Ecological, evolutionary & environmental sciences

For a reference copy of the document with all sections, see nature.com/documents/nr-reporting-summary-flat.pdf

Life sciences study design

All studies must disclose on these points even when the disclosure is negative.

Sample size	eriments was stated in the figure legends and/or supporting information files.
Data exclusions	No data were excluded from the
Replication	Unless explicitly stated, Regardless of the experiment, A minimum of three animal samp
Randomization	d body size were divided into experimental groups.
Blinding	Blinding was not relevant in this study, because there was no subjective analyses perform unlikely to affect the results

Behavioural & social sciences study design

All studies must disclose on these points even when the disclosure is negative.

Study description	
Research sample	
Sampling strategy	
Data collection	
Timing	
Data exclusions	
Non-participation	

Randomization

Ecological, evolutionary & environmental sciences study design

All studies must disclose on these points even when the disclosure is negative.

Study description

Research sample

Sampling strategy

Data collection

Timing and spatial scale

Data exclusions

Reproducibility

Randomization

Blinding

Did the study involve field work? ☐ Yes ☒ No

Field work, collection and transport

Field conditions

Location

Access & import/export

Disturbance

Reporting for specific materials, systems and methods

We require information from authors about some types of materials, experimental systems and methods used in many studies. Here, indicate whether each material, system or method listed is relevant to your study. If you are not sure if a list item applies to your research, read the appropriate section before selecting a response.

Materials & experimental systems

n/a	Involved in the study	
☐	☒	Antibodies
☐	☒	Eukaryotic cell lines
☒	☐	Palaeontology and archaeology
☐	☒	Animals and other organisms
☒	☐	Clinical data
☒	☐	Dual use research of concern

Methods

n/a	Involved in the study	
☒	☐	ChIP-seq
☒	☐	Flow cytometry
☒	☐	MRI-based neuroimaging

Antibodies

Antibodies used

1. anti-SYCP3 (Abcam, ab97672, IF:1:100)
2. anti-SYCP3 (Abcam, ab15093, IF:1:100)
3. anti-SYCP1 (Abcam, ab15090, IF:1:100)
4. anti-γH2AX (MerckMillipore, 16-202A, IF:1:800)
5. anti-TNP2 (Santa Cruz, sc-393843, IF:1:50)
6. anti-PRM2 (Briar Patch Biosciences, Hup 2B, IF:1:300)
7. anti-H4K8ac (ABclonal, A7258, IF:1:500)
8. anti-ELFN2 (Novus Biologicals, 90569, WB: 1:1000)
9. anti-H3 (Abcam, ab1791, WB: 1:2000)
10. anti-GAPDH (Abcam, ab8245, WB: 1:10000)
11. anti-TUBLIN (Sigma, T8328, WB: 1:2000)
12. anti-FLAG (Sigma, F3165, IP:10ug, WB:1:2000)
13. anti-rabbit IgG HRP-linked (ABclonal, AS014, WB: 1:5000)
14. anti-mouse IgG HRP-linked (ABclonal, AS003, WB: 1:5000)

Validation: Antibody validation information can be found on manufacturers' websites or published

Eukaryotic cell lines

Policy information about cell lines and Sex and Gender in Research

Cell line source(s): HEK293T was purchased from American Type Culture Collection (ATCC).

Authentication: Authentication was not performed as none of the cells we used have been listed in t

Mycoplasma contamination: Before conducting the experiments, mycoplasma was routinely checked on th

Commonly misidentified lines
(See ICLAC register) | None

Palaeontology and Archaeology

Specimen provenance | Provide provenance information for specimens and describe permits that were ob

Specimen deposition | Indicate where the specimens have been deposited to permit free access by othe

Dating methods | If new dates are provided, describe how they were obtained (e.g. collection, storag

Tick this box to confirm that the raw and calibrated dates are available in the paper or in Supplementary Information. ☐

Ethics oversight | Identify the organization(s) that approved or provided guidance on the study prot

Note that full information on the approval of the study protocol must also be provided in the manuscript.

Animals and other research organisms

Policy information about studies involving animals; ARRIVE guidelines recommended for reporting a

Laboratory animals | c57BL/6J mice were housed in an SPF animal facility with a light and dark cycle

Wild animals | No wild animals were used in this

Reporting on sex | All mice used in this study were male. Our study aimed to examine the functions of DDX43 in sperm

Field-collected samples | No field-collected samples were

Ethics oversight | The animal study protocols were approved by the Institutional Animal Care and Use

Note that full information on the approval of the study protocol must also be provided in the manuscript.

Clinical data

Policy information about clinical studies

All manuscripts should comply with the ICMJE guidelines for publication of clinical research and a completed CONSORT checklist must be included with all submissions.

Clinical trial registration | Provide the trial registration number from ClinicalTrials.gov or an eq

Study protocol | Note where the full trial protocol can be accessed OR if not available, explain

Data collection | Describe the settings and locales of data collection, noting the time periods of r

Outcomes | Describe how you pre-defined primary and secondary outcome measures and how you assessed

Dual use research of concern

Policy information about dual use research of concern

Hazards

Could the accidental, deliberate or reckless misuse of agents or technologies generated in the work, or the application of information presented in the manuscript, pose a threat to:

No	Yes	
☒	☐	Public health
☒	☐	National security
☒	☐	Crops and/or livestock
☒	☐	Ecosystems
☒	☐	Any other significant area

Experiments of concern

Does the work involve any of these experiments of concern:

No	Yes	
☒	☐	Demonstrate how to render a vaccine ineffective
☒	☐	Confer resistance to therapeutically useful antibiotics or antiviral agents
☒	☐	Enhance the virulence of a pathogen or render a nonpathogen virulent
☒	☐	Increase transmissibility of a pathogen
☒	☐	Alter the host range of a pathogen
☒	☐	Enable evasion of diagnostic/detection modalities
☒	☐	Enable the weaponization of a biological agent or toxin
☒	☐	Any other potentially harmful combination of experiments and agents

ChIP-seq

Data deposition

Confirm that both raw and final processed data have been deposited in a public database such as GEO. ☐

Confirm that you have deposited or provided access to graph files (e.g. BED files) for the called peaks. ☐

Data access links
May remain private before publication. | For "Initial submission" or "Revised version" documents, provide revie data

Files in database submission | Provide a list of all files available in the database

Genome browser session
(e.g. UCSC) | *Provide a link to an anonymized genome browser session for "Initial submission" and "Revised version" documents only, to enable peer review. Write "no longer applicable" for "Final submission" documents.*

Methodology

Replicates Describe the experimental replicates, specifying number, type and replicate

Sequencing depth Describe the sequencing depth for each experiment, providing the total number of

Antibodies Describe the antibodies used for the ChIP-seq experiments; as applicable, provide suppl

Peak calling parameters Specify the command line program and parameters used for read mapping and

Data quality Describe the methods used to ensure data quality in full detail, including how many p

Software Describe the software used to collect and analyze the ChIP-seq data. For custom code that

Flow Cytometry

Plots

Confirm that:

The axis labels state the marker and fluorochrome used (e.g. CD4-FITC). ☐

The axis scales are clearly visible. Include numbers along axes only for bottom left plot of group (a 'group' is an analysis of identical markers).

All plots are contour plots with outliers or pseudocolor plots. ☐

A numerical value for number of cells or percentage (with statistics) is provided. ☐

Methodology

Sample preparation Describe the sample preparation, detailing the biological source of the cells a

Instrument Identify the instrument used for data collection, specifying make and model

Software Describe the software used to collect and analyze the flow cytometry data. For custom coc

Cell population abundance Describe the abundance of the relevant cell populations within post-sort

Gating strategy Describe the gating strategy used for all relevant experiments, specifying the pre

Tick this box to confirm that a figure exemplifying the gating strategy is provided in the Supplementary Information. ☐

Magnetic resonance imaging

Experimental design

Design type Indicate task or resting state; event-related or block

Design specifications Specify the number of blocks, trials or experimental units per session and/o

Behavioral performance measures State number and/or type of variables recorded (e.g. correct butto

Acquisition

Imaging type(s) Specify: functional, structural, diffusion,

Field strength Specify in

Sequence & imaging parameters Specify the pulse sequence type (gradient echo, spin echo, etc.), in

Area of acquisition State whether a whole brain scan was used OR define the area of acquisition, c

Diffusion MRI ☐ Used ☐ Not used

Preprocessing

Preprocessing software Provide detail on software version and revision number and on specific para

Normalization If data were normalized/standardized, describe the approach(es): specify linear or r

Normalization template Describe the template used for normalization/transformation, specifying sub

Noise and artifact removal Describe your procedure(s) for artifact and structured noise removal, s

Volume censoring Define your software and/or method and criteria for volume censoring, and state t

Statistical modeling & inference

Model type and settings Specify type (mass univariate, multivariate, RSA, predictive, etc.) and de

Effect(s) tested Define precise effect in terms of the task or stimulus conditions instead of psyc

Specify type of analysis: ☐ Whole brain ☐ ROI-based ☐ Both

Statistic type for inference (See Eklund et al. 2016) — Specify voxel-wise or cluster-wise and report all relevant parameters for cluster-wise methods.

Correction Describe the type of correction and how it is obtained for multiple comparisons (e.g. F

Models & analysis

n/a	Involved in the study
☐	☐ Functional and/or effective connectivity
☐	☐ Graph analysis
☐	☐ Multivariate modeling or predictive analysis

Functional and/or effective connectivity Report the measures of dependence used and the model deta

Graph analysis Report the dependent variable and connectivity measure, specifying weighted graph c

Multivariate modeling and predictive analysis Specify independent variables, features extraction a

This checklist template is licensed under a Creative Commons Attribution 4.0 International License, which permits use, sharing, adaptation, distribution and reproduction in any medium or format, as long as you give appropriate credit to the original author(s) and the source, provide a link to the Creative Commons license, and indicate if changes were made. The images or other third party material in this article are included in the article's Creative Commons license, unless indicated otherwise in a credit line to the material. If material is not included in the article's Creative Commons license and your intended use is not permitted by statutory regulation or exceeds the permitted use, you will need to obtain permission directly from the copyright holder. To view a copy of this license, visit http://creativecommons.org/licenses/by/4.0/
